# Supplementary material for: Morphological characterization of antennae and antennal sensilla of Diaphorina citri Kuwayama (Hemiptera: Liviidae) nymphs
Source: PLoS One. 2020 Jun 3;15(6):e0234030. doi: 10.1371/journal.pone.0234030 (PMC7269239; doi:10.1371/journal.pone.0234030)
Supplement: S1 Fig — (DOC) [file pone.0234030.s001.doc]

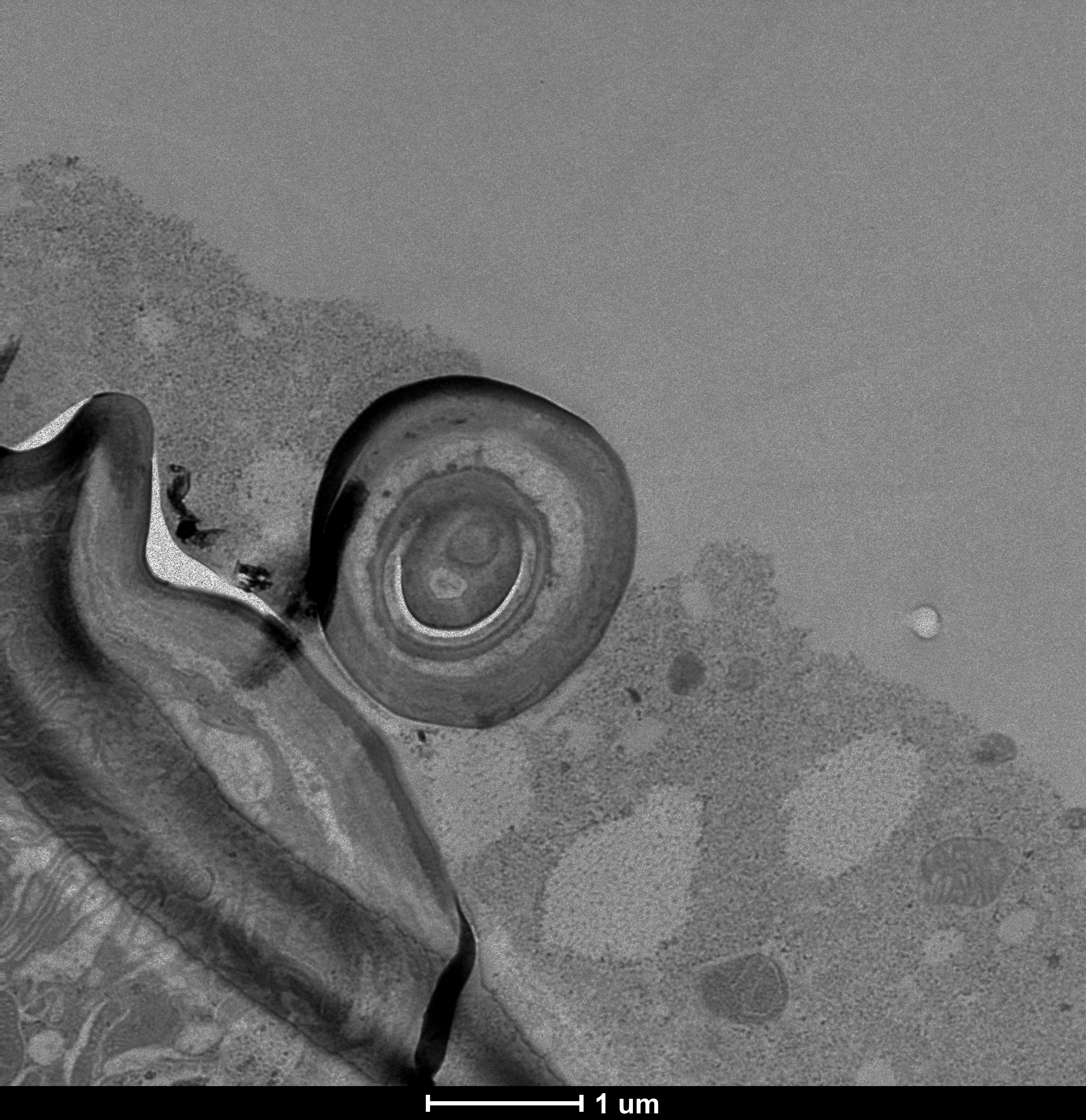


**1 μm**

**S1 Fig. Transmission electron microscopy micrograph of the transect through the cavity sensillum 1 of the first-instar *Diaphorina citri*, showing the two sensilla in the cavity (arrows).**
