# Supplementary material for: Morphological characterization of antennae and antennal sensilla of Diaphorina citri Kuwayama (Hemiptera: Liviidae) nymphs
Source: PLoS One. 2020 Jun 3;15(6):e0234030. doi: 10.1371/journal.pone.0234030 (PMC7269239; doi:10.1371/journal.pone.0234030)
Supplement: S2 Fig — (DOCX) [file pone.0234030.s002.docx]

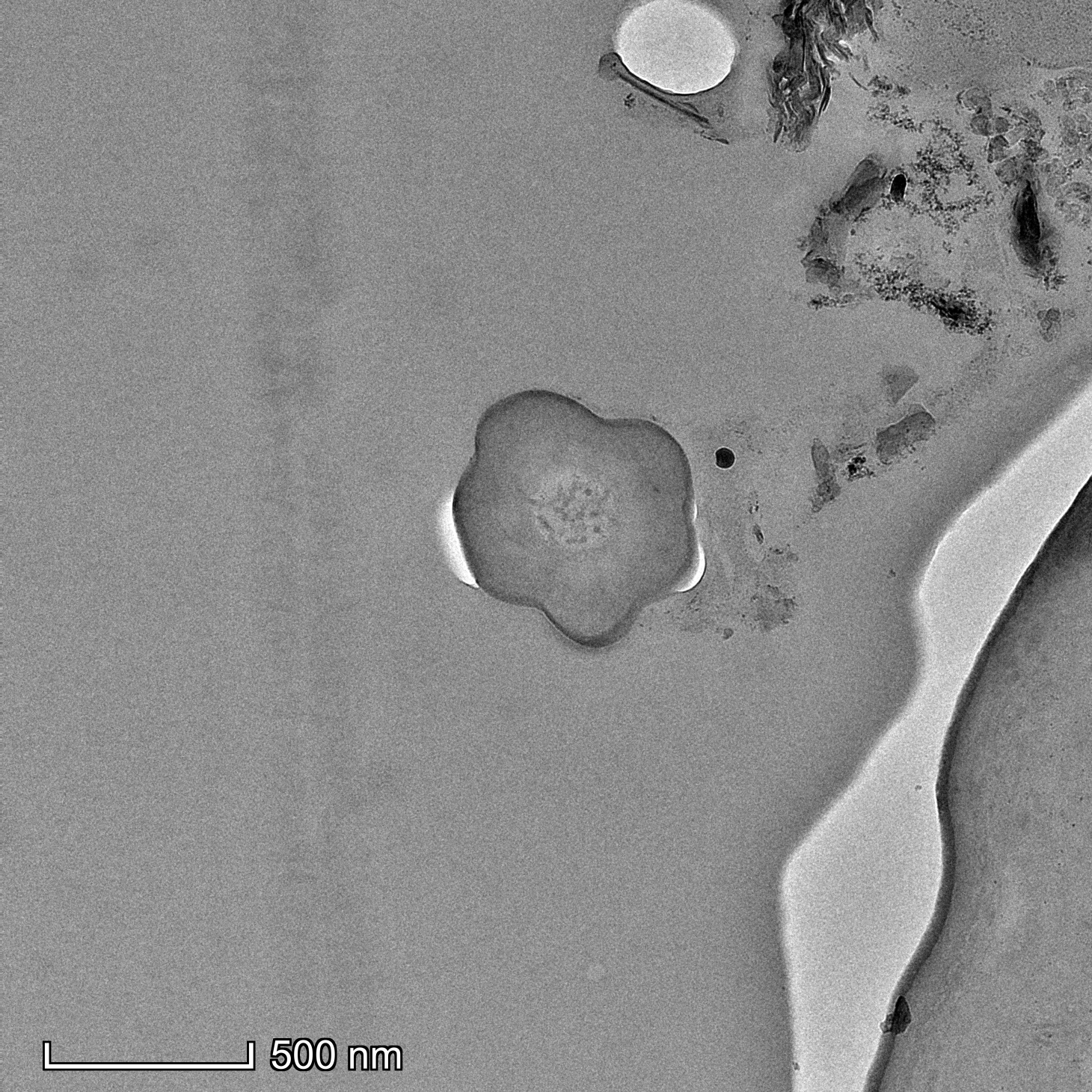


**500 nm**

**S2 Fig. Transmission electron microscopy micrograph of the sensilla trichoidea positioned below the base of the TH1 in the third-instar *Diaphorina citri* nymphs.**
