# Supplementary material for: Morphological characterization of antennae and antennal sensilla of Diaphorina citri Kuwayama (Hemiptera: Liviidae) nymphs
Source: PLoS One. 2020 Jun 3;15(6):e0234030. doi: 10.1371/journal.pone.0234030 (PMC7269239; doi:10.1371/journal.pone.0234030)
Supplement: S1 Table — (DOCX) [file pone.0234030.s003.docx]

**Table 1. Abundance and distribution of sensilla on the antennae of adult *Diaphorina citri***

| **Sensilla** | **Scape** | | **Pedicel** | | **Flagellum** | | | | | | | | |
| --- | --- | --- | --- | --- | --- | --- | --- | --- | --- | --- | --- | --- | --- |
|  | **Female** | **Male** | **Female** | **Male** | **F1** | **F2** | **F3** | **F4** | **F5** | **F6** | **F7** | **F8** | |
|  |  |  |  |  |  |  |  |  |  |  |  | **Female** | **Male** |
| ST1 | 12.50± 0.96a | 9.1±0.74b | 12.00±0.58a | 12.30±0.87a | — | — | — | — | — | — | — | — | — |
| ST2 | — | — | — | — | — | — | — | — | — | — | — | 1 | 1 |
| ST3 | — | — | — | — | 2 | 2 | 1 | 1 | 1 | 1 | 1 | 2 | 1 |
| ST4 | — | — | — | — | — | 1 | — | 1 | — | 1 | 1 | — | — |
| SCA | — | — | 1 | 1 | — | — | — | — | — | — | — | — | — |
| AR | — | — | — | — | — | 1 | — | 1 | — | 1 | 1 | — | — |
| TH1 | — | — | — | — | — | — | — | — | — | — | — | 1 | 1 |
| TH2 | — | — | — | — | — | — | — | — | — | — | — | 1 | 1 |
| Total | 12.5 | 9.1 | 13 | 13.3 | 2 | 4 | 1 | 3 | 1 | 3 | 3 | 5 | 4 |
| Number and location of the various sensilla observed on the antennal of adult *D. citri*. F1-F8, antennal flagellomeres 1-8; ST1, ST2, ST3 and ST4 are sensilla trichoidea 1, 2, 3 and 4, respectively; SCA, sensilla campaniform; SCO, sensilla coeloconia; TH1 and TH2 are long terminal hair and short terminal hair at the tip of the antennae. ‘—’, indicates sensilla absent. | | | | | | | | | | | | | |
