# Supplementary material for: Morphological characterization of antennae and antennal sensilla of Diaphorina citri Kuwayama (Hemiptera: Liviidae) nymphs
Source: PLoS One. 2020 Jun 3;15(6):e0234030. doi: 10.1371/journal.pone.0234030 (PMC7269239; doi:10.1371/journal.pone.0234030)
Supplement: S1 File — (DOC) [file pone.0234030.s004.doc]

**General analysis of the antennae and antennal sensilla of adult *Diaphorian citri*:** There was no sexually dimorphic found in the adult *D. citri* antennae (Fig 1). The female and male antennae were filiform in shape and composed of a scape, a pedicel, an eight-segmented flagellum, measuring 452.76 ± 23.77 μm and 460.99 ± 18.43 μm in length, respectively. There was no difference observed in the length of each segment between sexes. The width of the fifth flagellum in males was significantly bigger than that in females, but no difference in other flagella. The shortest antennal segments in females and males were the eighth flagellum, showing 31.56 ± 1.39 μm and 27.94 ± 2.24 μm, respectively. However, the longest segments were the scape (55.60 ± 3.35 μm) in females and the first flagellum (60.23 ± 3.68 μm) in males (Table 1). The entire surface of the flagellum was covered with annular scales. Eight morphologically different types of sensilla were observed on the antennae of both sexes. They were the long terminal hair (TH1) (Fig 2), short terminal hair (TH2) (Fig 2 and Fig 3), sensilla trichoidea 1 (ST1) (Fig 4A and Fig 4B), sensilla trichoidea 2 (ST2) (Fig C), sensilla trichoidea 3 (ST3) (Fig 4D), sensilla trichoidea 4 (ST4) (Fig 4E), sensilla campaniform (SCA) (Fig 5) and antennal rhinarium (AR) (Fig 6).


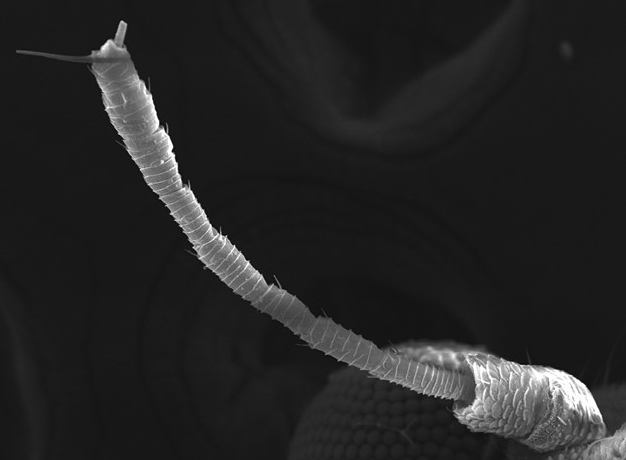


**100 μm**

**A**

**F1**

**F2**

**F3**

**F4**

**F5**

**F6**

**F7**

**F8**

**Flagellum**

**Pedicel**


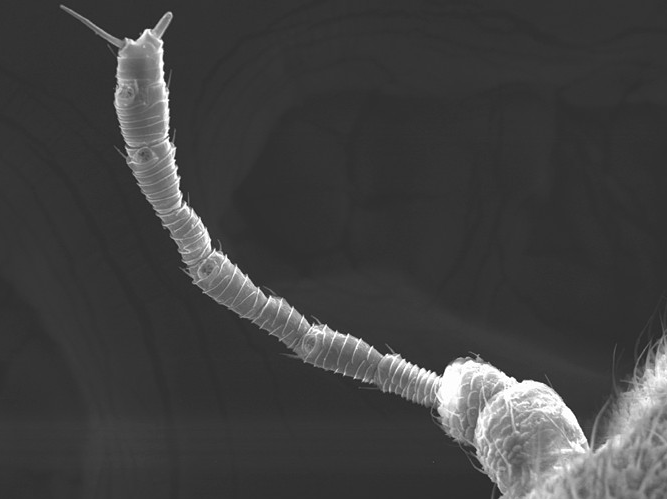


**100 μm**

**B**

**AR**

**TH2**

**TH1**

**Scape**

**Fig 1. General morphology of the antennae of adult *Diaphorina citri*.** (A) SEM photograph of the dorsal view of female *D. citri* antennae, showing the scape, pedicel, and flagellum. (B) SEM photograph of the ventral view of male *D. citri* antennae. F1, F2, F3, F4, F5, F6, F7, and F8 mean the eight flagella subsegments comprised of the flagellum. AR, antennal rhinarium; TH1, the long terminal hair; TH2, the short terminal hair.


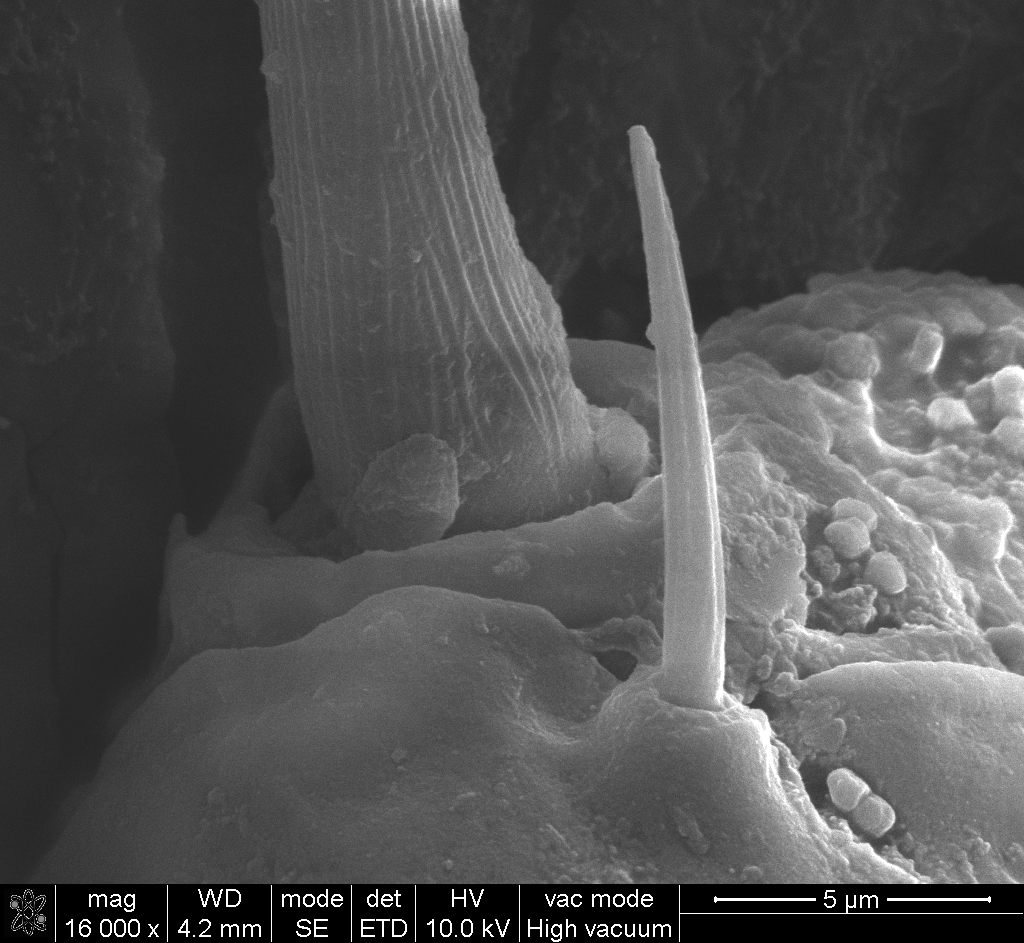


**C**

**Pores**

**5 μm**


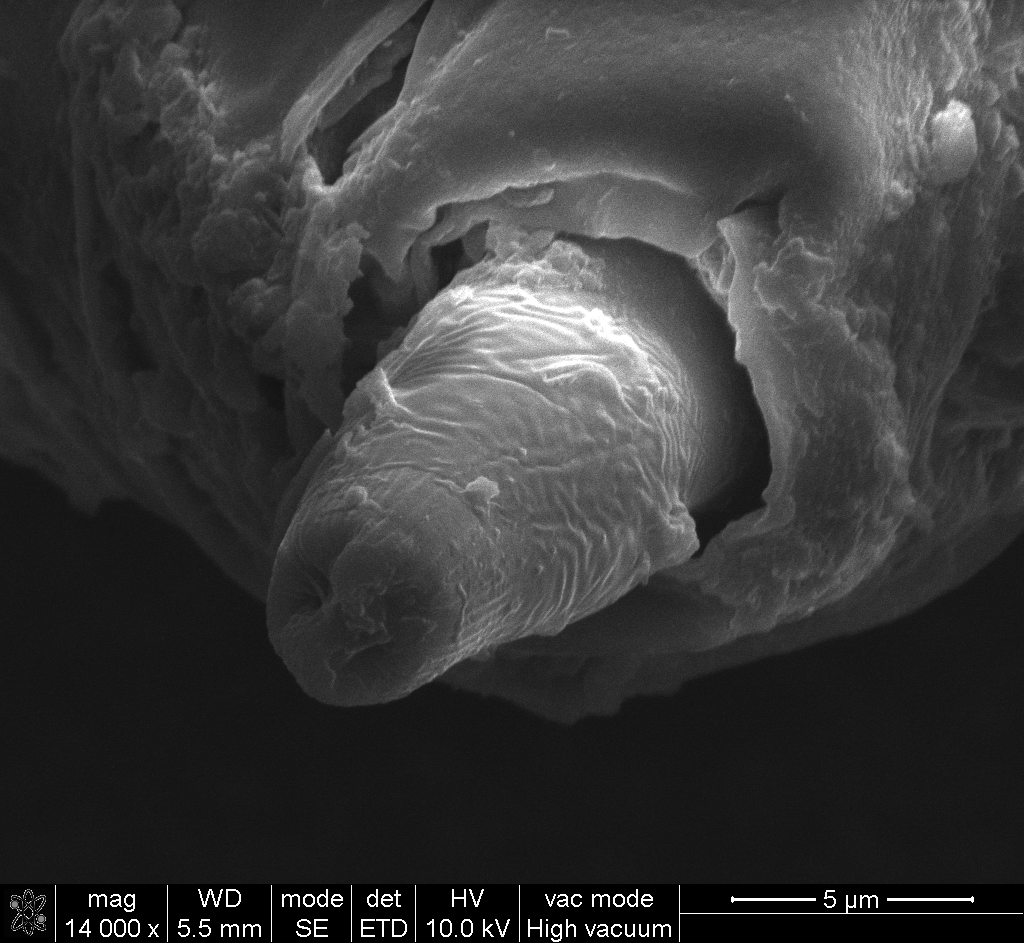


**B**

**Pores**

**5μm**


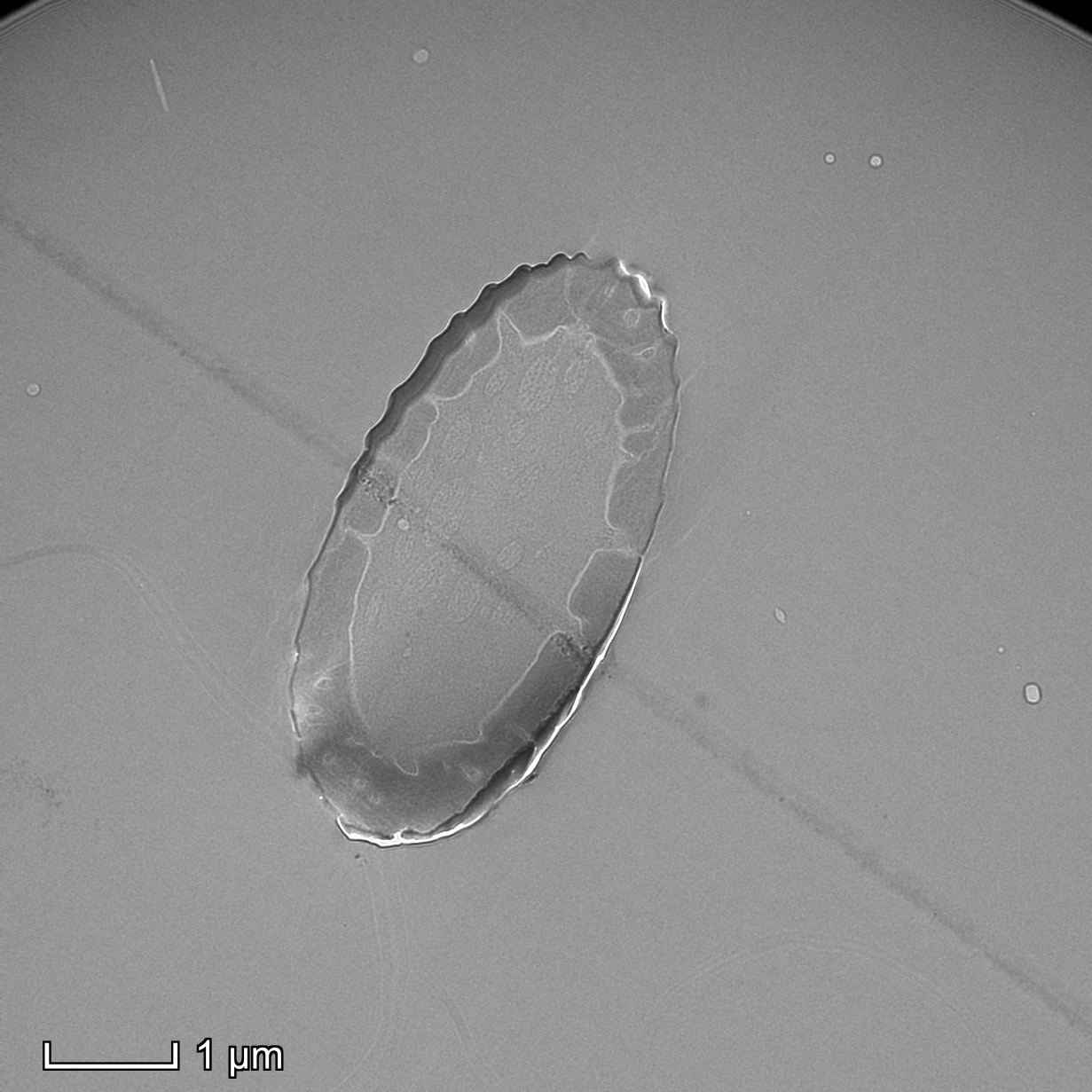


**1 μm**

**Pores**

**E**


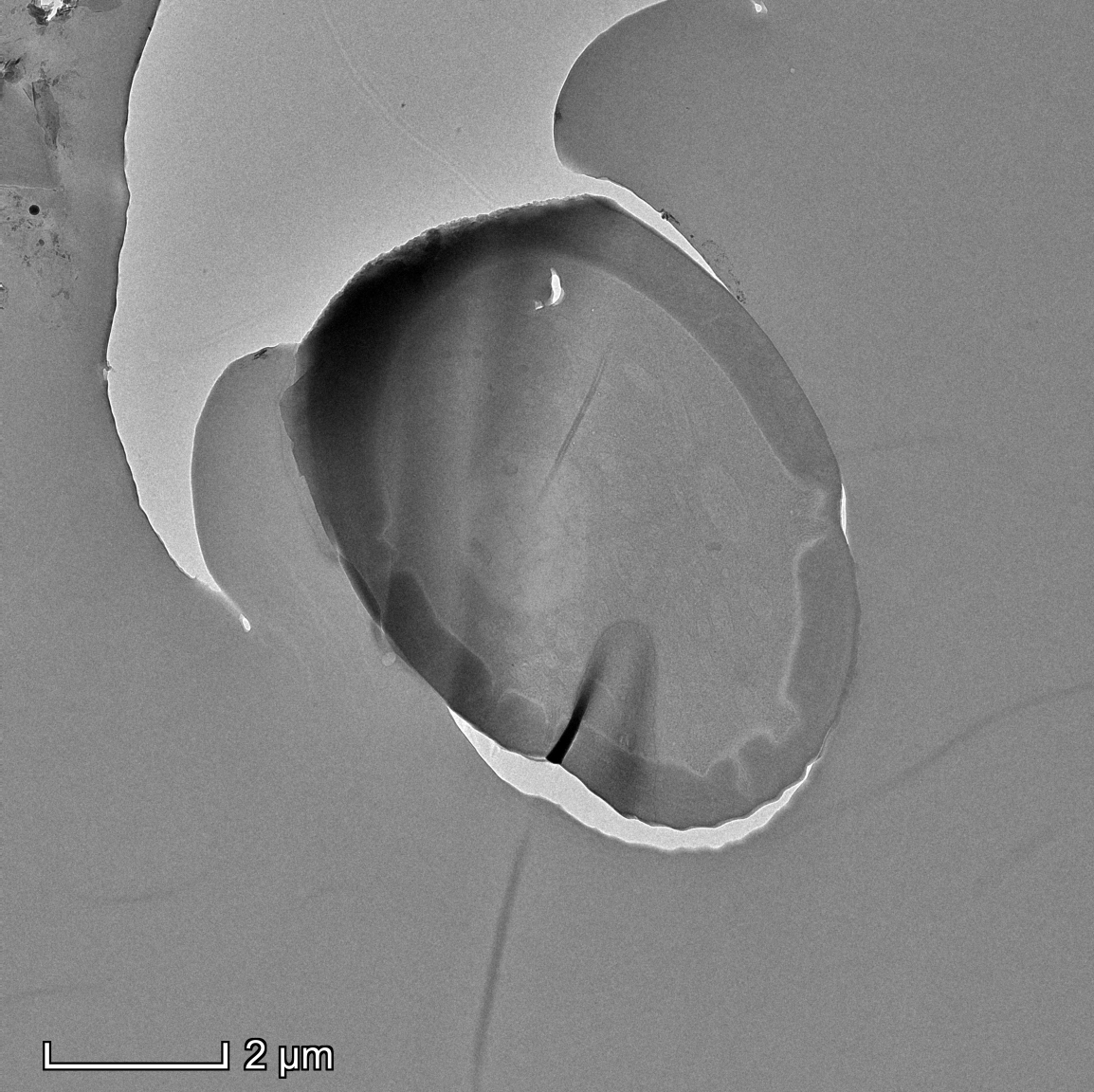


**D**

**2 μm**

**Pores**


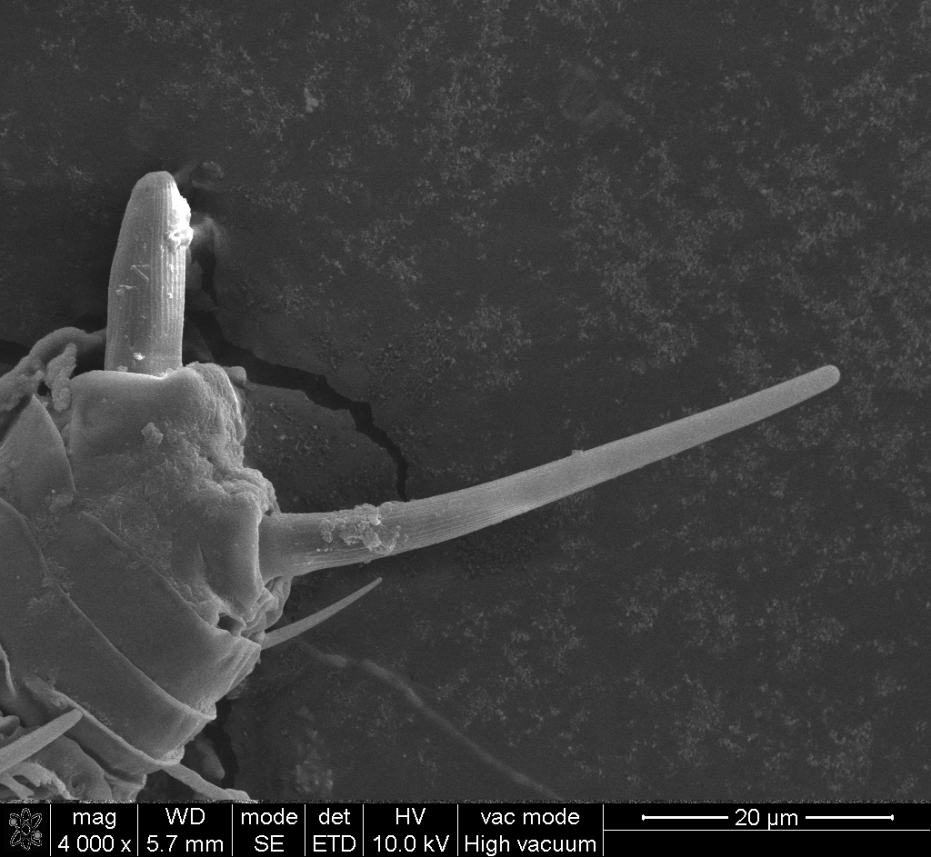


**A**

**20 μm**

**ST2**

**Fig 2.** **Two terminal hairs on the female antennae.** (A) SEM micrograph of the long and short terminal hairs. (B) The high magnification picture of the short terminal hair, showing the pores. (C) The high magnification picture of the long terminal hair, showing the pores. (D-E) TEM micrographs of the long and short terminal hair, respectively. ST2, sensilla trichoidea 2.


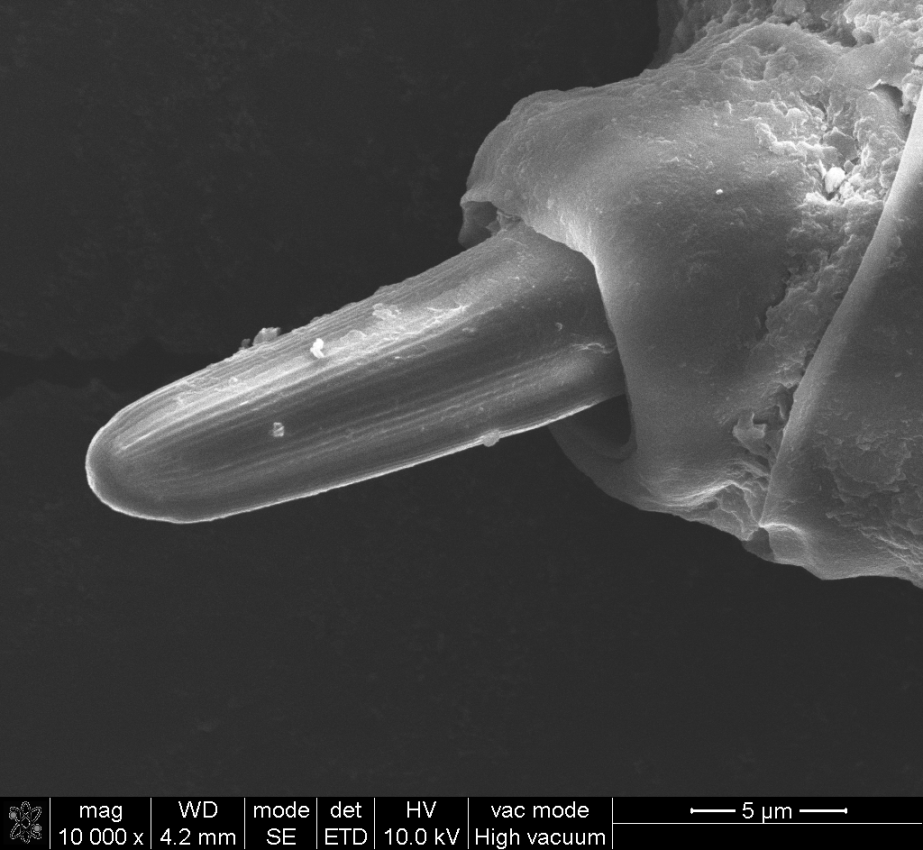


**A**

**5 μm**


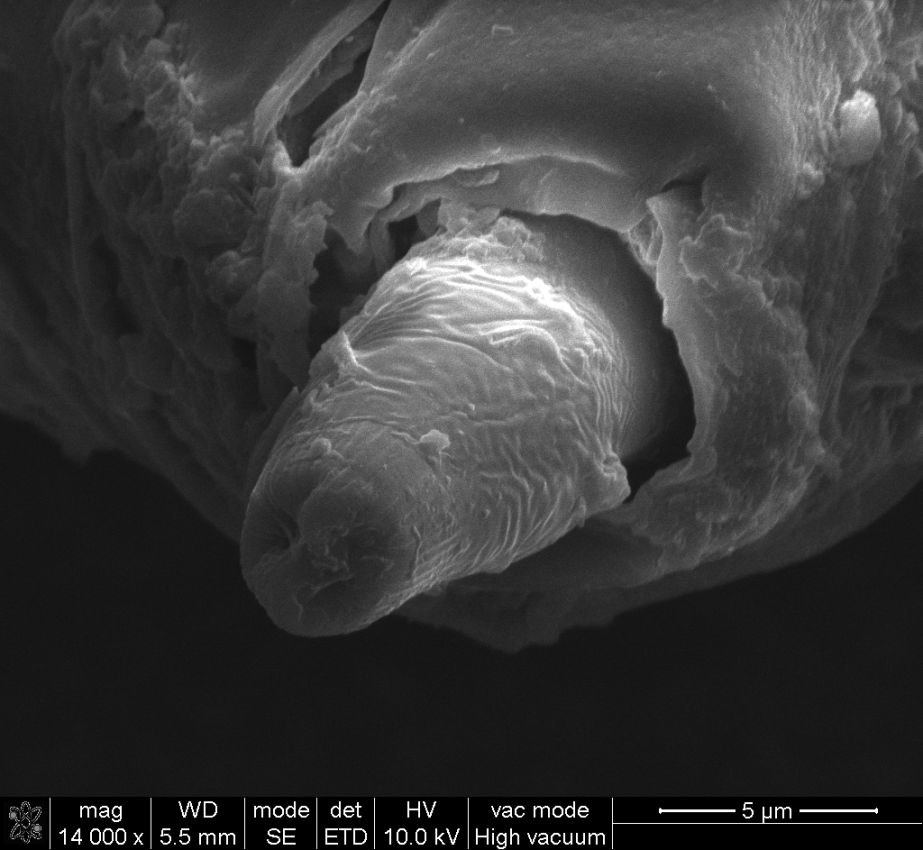


**B**

**5 μm**

**Fig 3.** **Sexual dimorphism of the short terminal hair (TH2) in male and female** ***Diaphorina citri*.** (A) TH2 in males with a blunt, un-recessed tip. (B) TH2 in females with a recessed tip.


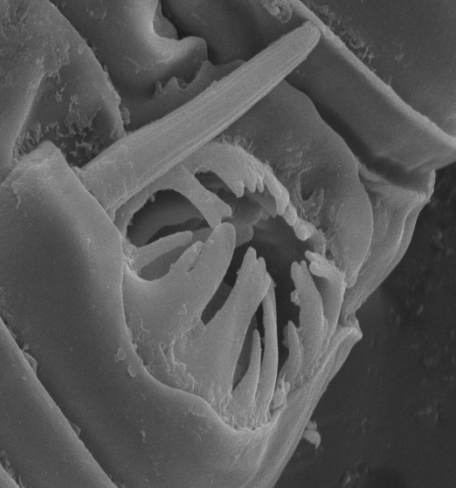


**E**

**5 μm**


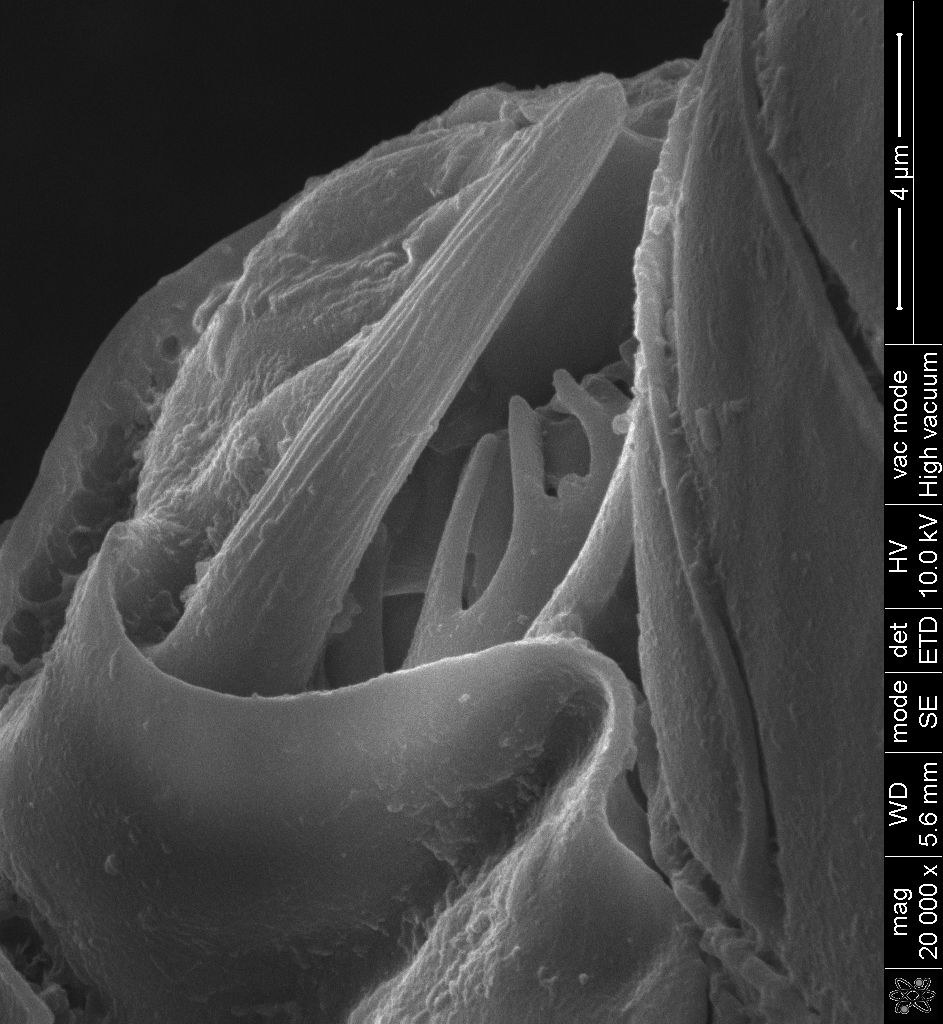


**4 μm**

**Pores**

**F**


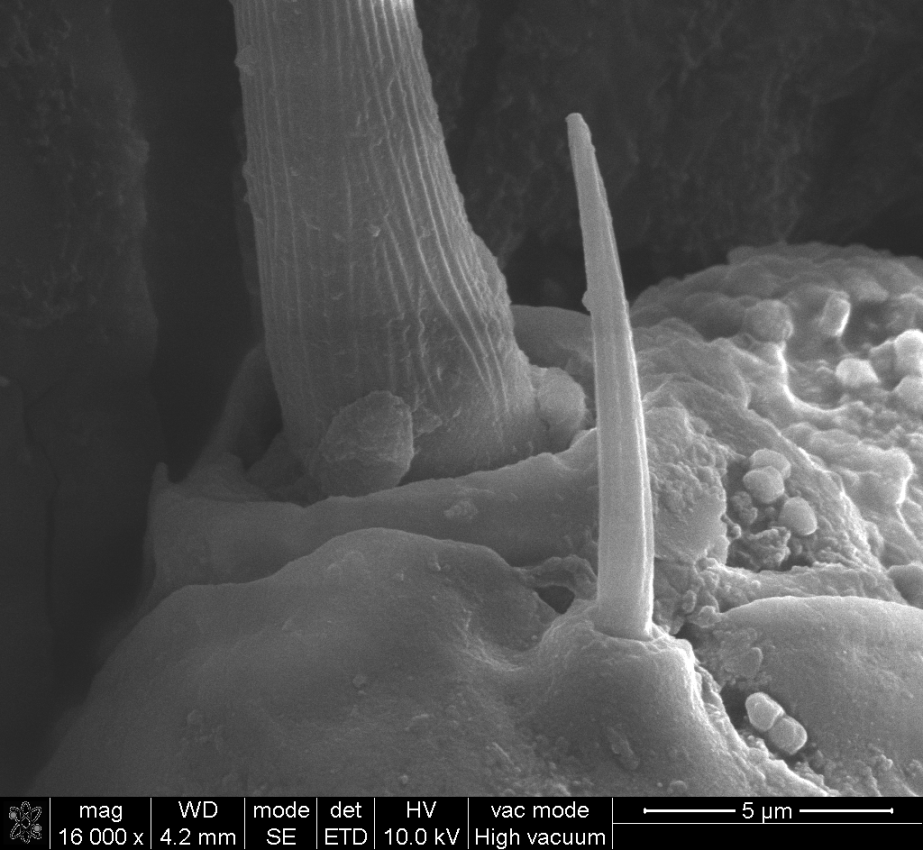


**C**

**5 μm**


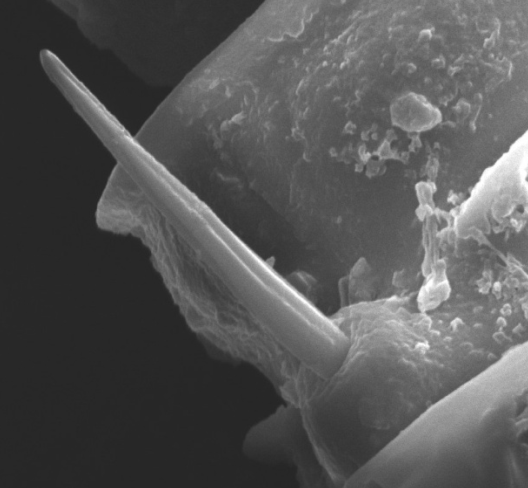


**D**

**4 μm**


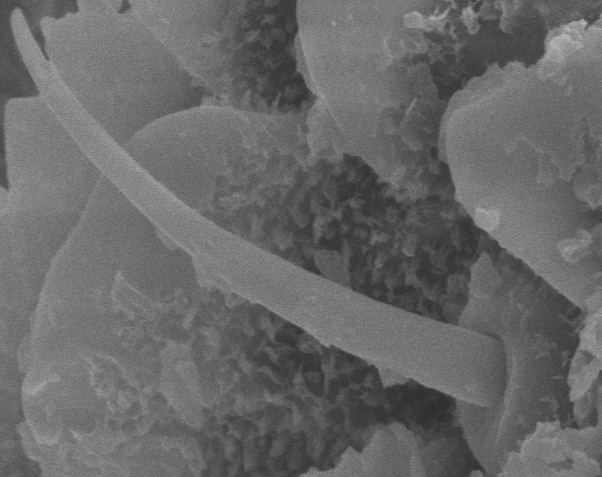


**A**

**4 μm**


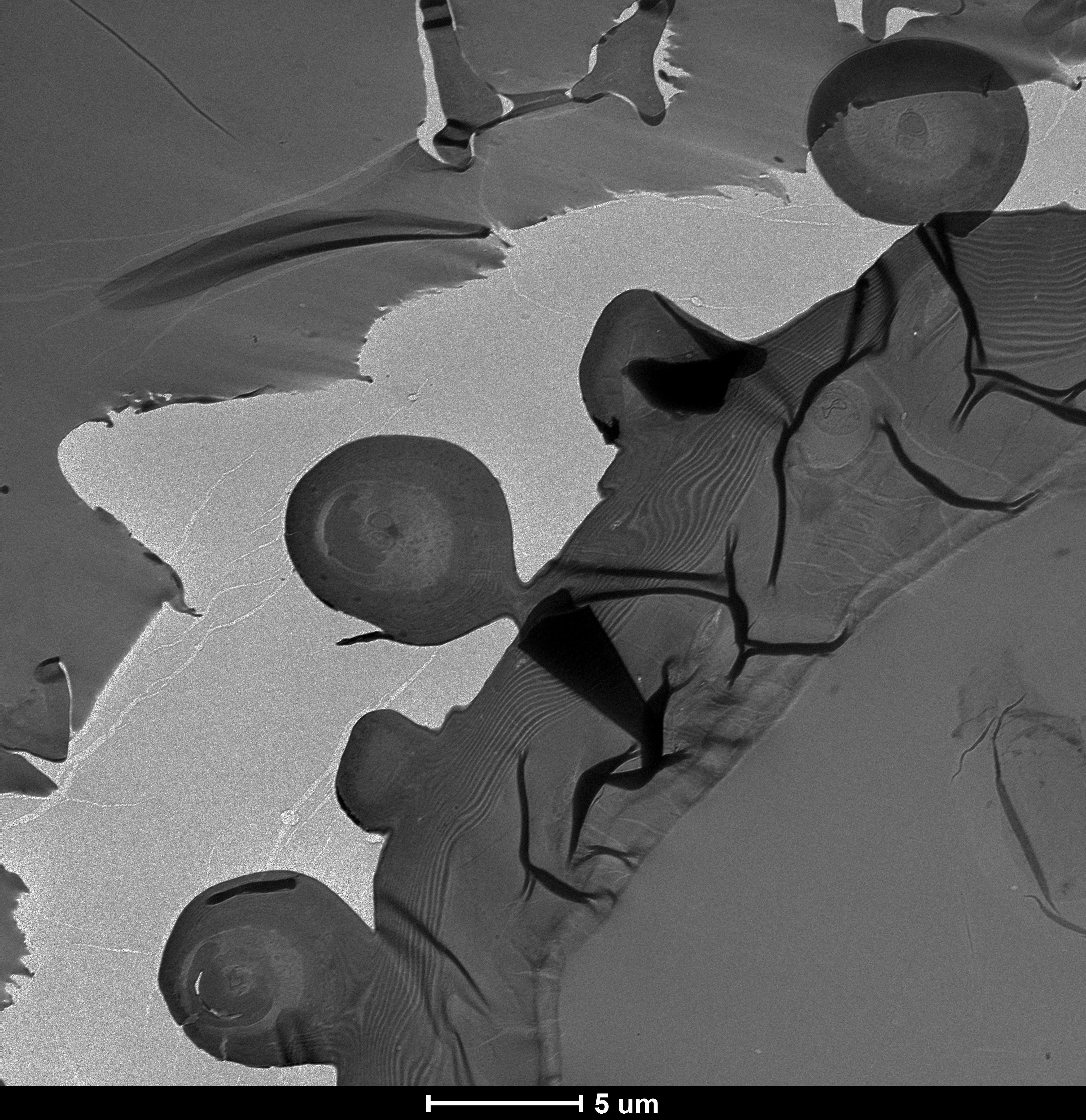


**5 μm**

**B**

**Pores**

**Fig 4.** **Sensilla trichoidea on the antennae of adult female *Diaphorina citri*.** (A) SEM micrograph of sensilla trichoidea 1. (B) TEM micrograph of the sensilla trichoidea 1. (C) Sensilla trichoidea 2. (D) Sensilla trichoidea 3. (E) Sensilla trichoidea 4. (F) The high magnification picture of the sensilla trichoidea 4, showing the pores.

**SCA**

**A**

**10 μm**


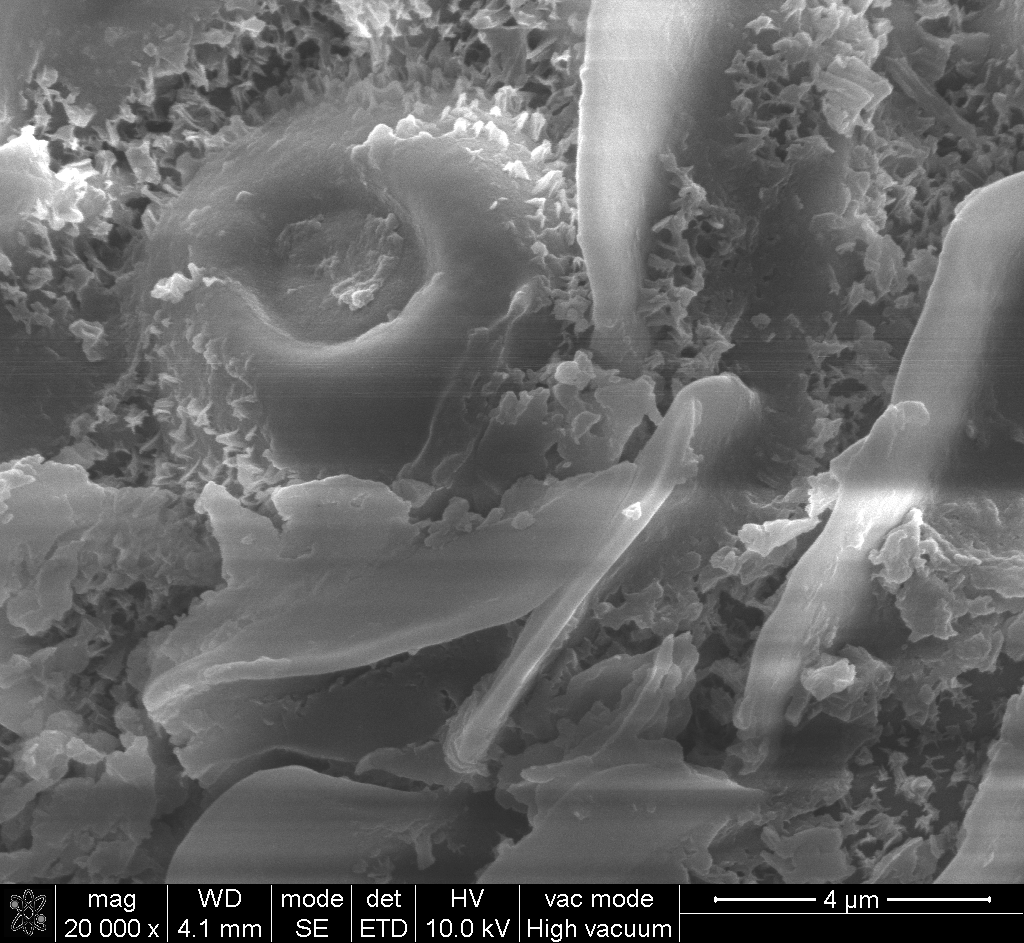


**B**

**4 μm**

**Fig 5. Sensilla campaniform (SCA) in females.** (A) Sensilla campaniform. (B) The high magnification picture of sensilla campaniform.


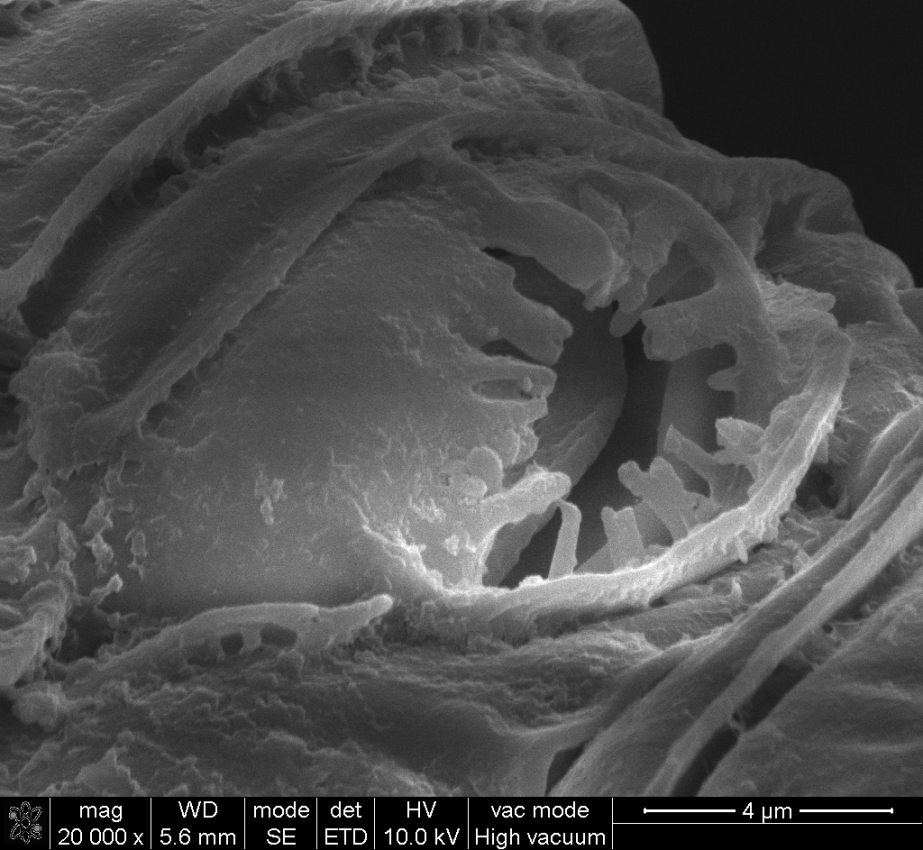


**4 μm**

**Pores**

**B**


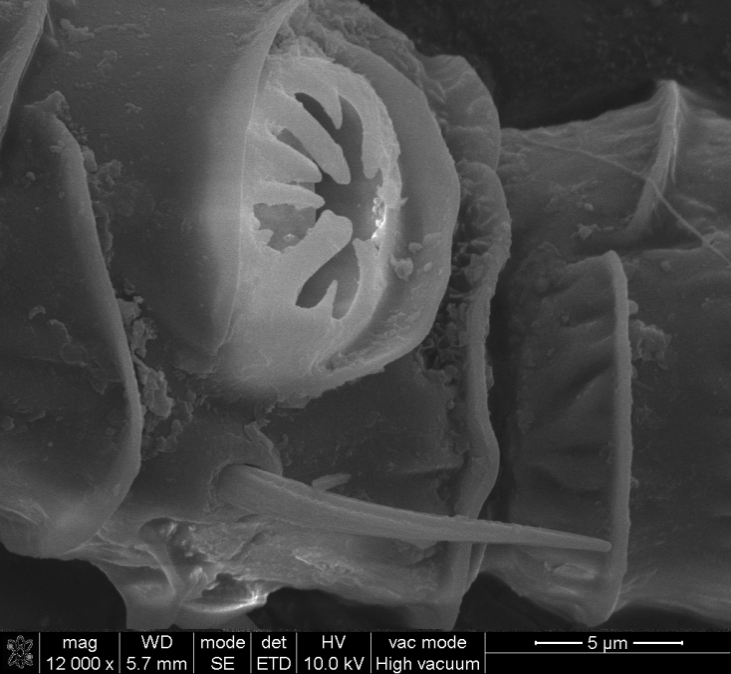


**5 μm**

**A**

**Cuticular fringe**

**Fig 6. Antennal rhinarium (AR) in males.** (A) Antennal rhinarium, showing the cuticular fringe. (B) Antennal rhinarium, showing the pores.

In adults, eight morphologically distinct types of sensilla were found to distribute on the antennae of female and male *D. citri* in our study, with seven types reminiscent of those described by Onagbola et al. (2008). The aporous sensilla trichoidea occurred on the medial portions of the scape, pedicel and flagellomeres 1, 2, 4 and 5, the chaetica sensilla and the unidentified uniporous sensilla which were reported by Onagbola et al. were absent, while the SCA were found in our study. In our study, we do not descript the morphology and structure of antennae and antennal sensilla of adult *D. citri*. Moreover, consistent with the previous observations (Onagbola et al., 2008), there was also a major sexual dimorphism identified only in the external morphology of the TH2, whose blunt tip was recessed inward in females but not in males (Fig 3). Moreover, the length of TH1 and TH2 was significant difference in the length and width (Table 2).

(Onagbola EO, Meyer WL, Boina DR, Stelinski LL. Morphological characterization of the antennal sensilla of the Asian citrus psyllid, *Diaphorina citri* Kuwayama (Hemiptera: Psyllidae), with reference to their probable functions. Micron. 2008; 39(8): 1184-1191.)

**Table 1. Mean length and width of antennal segments in female and male *Diaphorina citri* (*N*=10).**

| **Antennal segmengts** | | **Length(μm)** | | **Width(μm)** | |
| --- | --- | --- | --- | --- | --- |
|  |  | **Female** | **Male** | **Female** | **Male** |
| Scape |  | 55.60±3.35a | 46.96±3.17a | 64.63±1.67a | 64.66±2.27a |
| Pedicel |  | 40.18±2.20a | 39.17±2.93a | 50.75±0.98a | 52.92±1.79a |
|  | F1 | 51.73±5.22a | 60.23±3.68a | 21.10±0.67a | 20.93±1.24a |
|  | F2 | 50.11±2.52a | 54.75±2.13a | 24.60±1.49a | 26.55±2.58a |
|  | F3 | 42.04±1.32a | 42.90±2.46a | 18.47±0.60a | 21.64±2.17a |
|  | F4 | 50.78±1.39a | 54.10±1.56a | 23.54±0.35a | 24.43±0.95a |
|  | F5 | 47.96±2.09a | 46.27±2.67a | 18.15±0.41a | 20.30±0.67b |
|  | F6 | 46.15±1.20a | 49.09±2.55a | 27.95±0.86a | 29.09±0.86a |
|  | F7 | 36.66±1.00a | 39.58±1.62a | 34.02±1.02a | 36.57±0.88a |
|  | F8 | 31.56±1.39a | 27.94±2.24a | 27.35±1.00a | 27.74±0.82a |
| Total |  | 452.76±23.77a | 460.99±18.43a | — | — |
| Means in columns with same uppercase letters are not signiﬁcantly different (GLM, Tukey, *P*>0.05). Means in rows with same lowercase letters are not signiﬁcantly different (*P*>0.05) in Mann–Whitney *U* test. “－” indicates absent. | | | | | |

***Table 2. Length and width (mean ± SE, n=10) of TH1 and TH2 of various instars of Diaphorina citri.***

| **Stages** | **Length (μm)** | | **Width(μm)** | |
| --- | --- | --- | --- | --- |
|  | **TH1** | **TH2** | **TH1** | **TH2** |
| First-instar | 16.03±0.62BCa | 13.60±0.68ABb | 2.16±0.14Ca | 1.99±0.14Da |
| Second-instar | 13.38±0.66Ca | 9.88±0.33Bb | 2.37±0.20Ca | 2.45±0.09Da |
| Third-instar | 16.34±0.85BCa | 10.30±0.83Bb | 2.53±0.09Ca | 2.73±0.19CDa |
| Fourth-instar | 18.87±1.33BCa | 13.00±0.51ABb | 2.90±0.25BCa | 3.65±0.30Ca |
| Fifth-instar | 21.89±1.32Ba | 15.13±1.23Ab | 4.05±0.49ABa | 4.81±0.26Bb |
| Female adult | 51.99±0.80Aa | 15.14±1.11Ab | 4.39±0.32Aa | 6.94±0.26Ab |
| Male adult | 51.64±4.41Aa | 16.11±1.95Ab | 4.67±0.14Aa | 7.11±0.42Ab |
| Means in columns with same uppercase letters are not signiﬁcantly different (GLM, Tukey, *P*>0.05). Means in rows with same lowercase letters are not signiﬁcantly different (*P*>0.05) in Mann–Whitney *U* test. | | | | |
