# Supplementary material for: Morphological characterization of antennae and antennal sensilla of Diaphorina citri Kuwayama (Hemiptera: Liviidae) nymphs
Source: PLoS One. 2020 Jun 3;15(6):e0234030. doi: 10.1371/journal.pone.0234030 (PMC7269239; doi:10.1371/journal.pone.0234030)
Supplement: Attachment — Submitted filename: Reviewer general comments.pdf [file pone.0234030.s007.pdf]

General comments

The manuscript entitled 'Morphological characterization of nymphal antennae and antennal sensilla of *Diaphorina citri* Kuwayama (Hemiptera: Liviidae)' reports the investigation of general morphological aspects of immature *D. citri* antenna, as well the characterization of antennal sensillas among different nymphal instars. The manuscript is original; however, several points needs to be addressed before its publication.

Bellow, I will describe major points that should be addressed in order to justify the study or to avoid controversies. However, minor/detailed comments are noted in the pdf manuscript file.

Title: Asian citrus psyllid family: Liviidae instead Psyllidae

Introduction

1) The authors support the study of morphological characteristics of *D. citri* nymphs based on the fact that immature stages have the ability to transmit *Ca.* Liberibcter more efficiently than adults. This argument is used to justify the choice for immature stages. However, we cannot forget that adults can spread bacteria more efficiently due its flight (from plant to plant). Another aspect that supports the HLB spread is the fact that *D. citri* adults, at first, prefers HLB plants (regulated by MeSa Vocs) and after feeding on HLB plants, move the health plants (better nutritional aspects). I am not sure about the author´s sentence to justify the studies only with nymphs.
2) The entire literature related to ACP/chemical ecology are based on adult ACP behavior challenged by chemicals from plants or from co-specific insects and their VOCs. Some manuscripts explain the role of ACP adult antenna receptors challenged by plant and/or chemical volatiles. It is not possible to understand the relationship of immature ACP stages with chemicals, since nymphs do not perform choices in response to chemicals, since nymphs do not perform movements. Thus, I still not convicted about the choice for nymphs only. However, the comparison of nymph antenna structures/sensillas with adults could be helpful for other studies and if done, could be more easy to justify the study.
3) However, the authors cited 2 articles that assessed the antennal structures of adults. However, due to the lack of information (see your discussion topic), the authors are unable to explain the absence of these structures during the investigation of these articles. Maybe there is the absence of these structures in these articles, or the cited authors did not see the mentioned structures. In order to solve this doubt, I encourage the authors to add morphological aspects of adult antenna, since the literature was not helpful to support the findings with nymphs and comparison with adults.
4) In general, the introduction is not justified neither well presented. I suggest that this topic could be re-written and that in M&M and Results could be added adult antennal structures.

Material and Methods

There is a lack of substantial information that allow the replication of the protocols. There is no enough information related to the insect (rearing protocol, age, description of what the authors considered as nymph 1, nymph 2, nymph 3, nymph 4 and nymph 5 (see pdf manuscript with my notes).

Number of replicates.

More details about statistical analyses (see pdf manuscript with my notes).

Discussion

The beginning of discussion presents text that is related to results (see pdf manuscript with my notes).

The authors are not convicted if antennal structures present in nymphs were degenerated or Onagbola et al did not see this structure in adults (but could be present). This fact did not allow the authors to be convincing during the discussion of the topic. A solution could be the assessment of adult antennal morphological aspects in order to be clear and consistent/convicted to explain the 'possible degeneration' of these structures in ACP adults.

Tables and Figures

Figures should be self-explained (comments in pdf manuscript)

Reviewer decision

These findings (assessment in adults) and explanation of differences in nymphs/adult antennal morphological aspects will be very helpful for ACP chemical ecology/ACP behavioral studies. However, the lack of these information compromises the manuscript findings and new discoveries/tools that could increase the knowledge in this field.

In this way, I suggest that the manuscript should not be recommended for publication in PLOS ONE in this form. However, the addition of adult ACP findings and a better explanation/justification of the study could be helpful for a future peer-reviewer round.
