# Supplementary material for: Morphological characterization of antennae and antennal sensilla of Diaphorina citri Kuwayama (Hemiptera: Liviidae) nymphs
Source: PLoS One. 2020 Jun 3;15(6):e0234030. doi: 10.1371/journal.pone.0234030 (PMC7269239; doi:10.1371/journal.pone.0234030)
Supplement: Attachment — Submitted filename: PONE-D-19-23769_R1_reviewer.pdf [file pone.0234030.s008.pdf]

PLOS ONE

Morphological characterization of nymphal antennae and antennal sensilla of Diaphorina citri Kuwayama (Hemiptera: Psyllidae)

--Manuscript Draft--

**Manuscript Number:**	PONE-D-19-23769R1
**Article Type:**	Research Article
**Full Title:**	Morphological characterization of nymphal antennae and antennal sensilla of Diaphorina citri Kuwayama (Hemiptera: Psyllidae)
**Short Title:**	Antennal sensilla ultrastructure of Diaphorina citri of various nymphal stages
**Corresponding Author:**	Li-Xia Zheng<br>Foshan University<br>Foshan, CHINA
**Keywords:**	Diaphorina citri Kuwayama; antennal sensilla; nymph; SEM; structure; psyllid; Morphology
**Abstract:**	Diaphorina citri Kuwayama is an economically important citrus pest which is the primary vector of Candidatus Liberibacter spp. causing citrus greening (huanglongbing, HLB) disease. Because its ability to transmit C. Liberibacter was more efficient in nymphs, we investigated the antennal morphology, structure and sensilla distribution in five nymphal stages of D. citri using scanning electron microscopy. The antennae of five different nymphal stages were filiform in shape, which consisted of two segments in the first-, second- and third-instar nymphs; three segments in the fourth- and fifth-instar nymphs. The length of their antennae was significantly increased with the increase of the instars, as well as the total number of antennal sensilla. Eleven morphological sensilla types were recorded altogether. They were the long terminal hair (TH1), short terminal hair (TH2), sensilla trichoidea (ST), cavity sensillum 1 (CvS1), cavity sensillum 2 (CvS2), sensilla basiconica 1-4 (SB1-4), sensilla campaniform (SCA) and partitioned sensory organ (PSO). Also, the distribution of antennal sensilla in each nymphal stage of D. citri was asymmetrical. The SB only occurred on the antennae of the third-, fourth- and fifth-instar nymphs. Only one CvS2 was found in the third- and fifth-instar nymphs, and one SCA in the fourth- and fifth-instar nymphs, respectively. The possible roles of the nymphal antennal sensilla in D. citri were discussed. The results could contribute to further explore the relationship between the ability to carry and transmit C. Liberibacter and the feeding behavior of the nymphal D. citri.
**Order of Authors:**	Li-Xia Zheng
	Wen-Sheng Chen
**Response to Reviewers:**	We have studied the comments carefully and have made correction which we hope meet with approval. The main corrections in the paper and the responds to the comments are as flowing:<br><br>Responds:<br>1 Change into black all red letters left in the m/s.<br>Response: Thank you very much. We have done and checked several times.<br><br>2 send the m/s for a second cycle of editorial processing; there are many errors, such as DiscuRsion, flower shape vs. flower shaped, The PSO formed with an opened..., the first segment of the antennae was absent sensilla, citrus psylla (the official common name for D. citri is Asian Citrus Psyllid (ACP), Acknowledge, to cite a few (please have a thorough review).<br>Response: We are grateful to Professor Walter S. Leal for the nice and good comments. We are very sorry for the errors which should not be made. So, we have corrected the errors not only listed by Professor Walter S. Leal, but also revised some other errors. Revised portion can be found in the file “Revised Manuscript with Track Changes” easily.<br><br>3 the literature is outdated; to a minimum you have to cite the work of Illiano Coutinho

Powered by Editorial Manager® and ProduXion Manager® from Aries Systems Corporation

	when referring to D. citri antennal structure; our two papers on pheromones (please perform an in-depth literature search and update your references).<br>Response: Thank you very much for the good suggestions. In fact, we have read these two references ("Zanardi OZ, Volpe HXL, Favaris AP, Silva WD, Luvizotto RAG, Magnani RF, et al. Putative sex pheromone of the Asian citrus psyllid, Diaphorina citri, breaks down into an attractant. Scientific Reports. 2018; 8(1): 455."; "Laboratory and field evaluation of acetic acid-based lures for male Asian citrus psyllid, Diaphorina citri. Scientific Reports. 2019; 9(1): 1-10."), but we do not know how to refer. Our teams also work for the sex pheromone of the ACP. You can find these new references in the page 2 lines55-59.<br><br>4 Follow PLoS ONE instructions (Tables go in the Main Text).<br>Response: Done. Thank you!<br><br>5 Improve figure legends; some of them just have a title.<br>Response: Done. Thank you!<br><br>6 Bring relevant TEM figures to the main text.<br>Response: Thank you very much for the suggestion. However, the TEM figures of the five different nymphal stages are very poor. We do think they can meet the requirements of PLoS One.<br><br>We tried our best to improve the manuscript and made some changes in the manuscript. We appreciate for warm work of Professor Walter S. Leal earnestly, and hope that the correction will meet with approval.<br>Once again, thank you very much for your comments and suggestions.
**Additional Information:**	
**Question**	**Response**
**Financial Disclosure**<br><br>Enter a financial disclosure statement that describes the sources of funding for the work included in this submission. Review the submission guidelines for detailed requirements. View published research articles from *PLOS ONE* for specific examples.<br><br>This statement is required for submission and **will appear in the published article** if the submission is accepted. Please make sure it is accurate.	This research was supported by the National Natural Science Foundation of China (31660517) and the Natural Science Foundation of Jiangxi Province, China (20161BAB214172).

**Unfunded studies**<br>Enter: *The author(s) received no specific funding for this work.*<br><br>**Funded studies**<br>Enter a statement with the following details:<br>• Initials of the authors who received each award<br>• Grant numbers awarded to each author<br>• The full name of each funder<br>• URL of each funder website<br>• Did the sponsors or funders play any role in the study design, data collection and analysis, decision to publish, or preparation of the manuscript?<br>• **NO** - Include this sentence at the end of your statement: *The funders had no role in study design, data collection and analysis, decision to publish, or preparation of the manuscript.*<br>• **YES** - Specify the role(s) played.<br><br>* typeset	
**Competing Interests**<br><br>Use the instructions below to enter a competing interest statement for this submission. On behalf of all authors, disclose any competing interests that could be perceived to bias this work—acknowledging all financial support and any other relevant financial or non-financial competing interests.<br><br>This statement **will appear in the published article** if the submission is accepted. Please make sure it is accurate. View published research articles from *PLOS ONE* for specific examples.	The authors have declared that no competing interests exist.

**NO authors have competing interests**<br><br>Enter: *The authors have declared that no competing interests exist.*<br><br>**Authors with competing interests**<br><br>Enter competing interest details beginning with this statement:<br><br>*I have read the journal's policy and the authors of this manuscript have the following competing interests: [insert competing interests here]*<br><br>* typeset	
**Ethics Statement**<br><br>Enter an ethics statement for this submission. This statement is required if the study involved:<br><br>• Human participants<br>• Human specimens or tissue<br>• Vertebrate animals or cephalopods<br>• Vertebrate embryos or tissues<br>• Field research<br><br>Write "N/A" if the submission does not require an ethics statement.<br><br>General guidance is provided below. Consult the submission guidelines for detailed instructions. **Make sure that all information entered here is included in the Methods section of the manuscript.**	The research meets all applicable standards for the ethics of experimentation and research integrity.

**Format for specific study types**<br><br>**Human Subject Research (involving human participants and/or tissue)**<br>• Give the name of the institutional review board or ethics committee that approved the study<br>• Include the approval number and/or a statement indicating approval of this research<br>• Indicate the form of consent obtained (written/oral) or the reason that consent was not obtained (e.g. the data were analyzed anonymously)<br><br>**Animal Research (involving vertebrate animals, embryos or tissues)**<br>• Provide the name of the Institutional Animal Care and Use Committee (IACUC) or other relevant ethics board that reviewed the study protocol, and indicate whether they approved this research or granted a formal waiver of ethical approval<br>• Include an approval number if one was obtained<br>• If the study involved *non-human primates*, add *additional details* about animal welfare and steps taken to ameliorate suffering<br>• If anesthesia, euthanasia, or any kind of animal sacrifice is part of the study, include briefly which substances and/or methods were applied<br><br>**Field Research**<br><br>Include the following details if this study involves the collection of plant, animal, or other materials from a natural setting:<br>• Field permit number<br>• Name of the institution or relevant body that granted permission	
**Data Availability**<br><br>Authors are required to make all data underlying the findings described fully available, without restriction, and from the time of publication. PLOS allows rare exceptions to address legal and ethical concerns. See the PLOS Data Policy and FAQ for detailed information.	Yes - all data are fully available without restriction

Powered by Editorial Manager® and ProduXion Manager® from Aries Systems Corporation

A Data Availability Statement describing where the data can be found is required at submission. Your answers to this question constitute the Data Availability Statement and **will be published in the article**, if accepted.<br><br>**Important:** Stating 'data available on request from the author' is not sufficient. If your data are only available upon request, select 'No' for the first question and explain your exceptional situation in the text box.<br><br>Do the authors confirm that all data underlying the findings described in their manuscript are fully available without restriction?	
**Describe where the data may be found in full sentences. If you are copying our sample text, replace any instances of XXX with the appropriate details.**<br><br>• If the data are **held or will be held in a public repository**, include URLs, accession numbers or DOIs. If this information will only be available after acceptance, indicate this by ticking the box below. For example: *All XXX files are available from the XXX database (accession number(s) XXX, XXX.)*.<br>• If the data are all contained **within the manuscript and/or Supporting Information files**, enter the following: *All relevant data are within the manuscript and its Supporting Information files.*<br>• If neither of these applies but you are able to provide **details of access elsewhere**, with or without limitations, please do so. For example:<br><br>*Data cannot be shared publicly because of [XXX]. Data are available from the XXX Institutional Data Access / Ethics Committee (contact via XXX) for researchers who meet the criteria for access to confidential data.*<br><br>*The data underlying the results presented in the study are available from (include the name of the third party*	All relevant data are within the manuscript and its Supporting Information files.

and contact information or URL).<br>• This text is appropriate if the data are owned by a third party and authors do not have permission to share the data.<br><br>* typeset	
Additional data availability information:	

Powered by Editorial Manager® and ProduXion Manager® from Aries Systems Corporation

Manuscript
Click here to access/download;Manuscript;Manuscript.docx

Morphological characterization of nymphal antennae and antennal sensilla of *Diaphorina citri* Kuwayama (Hemiptera: Psyllidae)

Lixia Zheng[1,2]*, Wensheng Chen[1]

1 College of Horticulture and Food Sciences, Foshan University, Foshan, China, **2** College of Agronomy, Jiangxi Agricultural University, Nanchang, China.

*lxzheng@fosu.edu.cn; lxzheng@jxau.edu.cn (LZ)

Abstract

Diaphorina citri Kuwayama is an economically important citrus pest which is the primary vector of *Candidatus* Liberibacter spp. causing citrus greening (huanglongbing, HLB) disease. Because its ability to transmit *C.* Liberibacter was more efficient in nymphs, we investigated the antennal morphology, structure and sensilla distribution in five nymphal stages of *D. citri* using scanning electron microscopy. The antennae of five different nymphal stages were filiform in shape, which consisted of two [illegible]ments in the first-, second- and third-instar nymphs; three segments in the fourth- and fifth-instar nymphs. The length of their antennae was significantly increased with the increase of the instars, as well as the total number of antennal sensilla. Eleven morphological sensilla types were recorded altogether. They were the long terminal hair (TH1), short terminal hair (TH2), sensilla trichoidea (ST), cavity sensillum 1 (CvS1), cavity sensillum 2 (CvS2), sensilla basiconica 1-4 (SB1-4), sensilla campaniform (SCA) and partitioned sensory organ (PSO). Also, the distribution of antennal sensilla in each nymphal stage of *D. citri* was asymmetrical. The SB only occurred on the antennae of the third-, fourth- and fifth-instar nymphs. Only one CvS2 was found in the third- and fifth-instar nymphs, and one SCA in the fourth- and fifth-instar nymphs, respectively. The possible roles of the nymphal

antennal sensilla in *D. citri* were discussed. The results could contribute to further explore the relationship between the ability to carry and transmit *C.* Liberibacter and the feeding behavior of the nymphal *D. citri*.

Introduction

The Asian citrus psyllid (ACP), *Diaphorina citri* Kuwayama (Hemiptera: Psyllidae), is an economically important citrus pest because it is the primary vector of *Candidatus* Liberibacter spp. causing citrus greening (huanglongbing, HLB) disease, the most serious citrus disease worldwide [1,2]. *D. citri* apparently originated in southern Asia, but now has spread to other citrus-producing areas of the world [2]. More than 50 species of the family Rutaceae have been recorded as hosts of *D. citri* [3]. Nymphs and adults suck phloem sap from the foliage causing leaf distortion, curling and deposition of honeydew on leaves that result in mold growth [3,4]. However, the most serious threat to citrus worldwide comes from its ability to transmit the bacterium, *C.* Liberibacter spp., the causal organism of HLB which causes yellowing of shoots, stem dieback, sour fruit, crop losses and eventually tree death [5,6]. The nymphs and adults of *D. citri* feed on plant sap and the pathogen can only be transmitted by the third-, fourth- and fifth-instar nymphs and adults which play a major role in the spread of the pathogen because of their dispersal capabilities [2,7-9], but the nymphs were found to be more efficient [4].

For most insects, the antennae are peripheral sensory structures involved in habitat searching, host location, host discrimination, mating and oviposition [10-12]. They carry different kinds of sensilla which can be classified as chemosensory, mechanosensory and thermo-hygroreceptive sensilla based on their morphological and functional characteristics [10,13]. Studies on the interaction between ACP and host plant volatiles indicated the critical role of its olfactory system in finding host plants [14,15], mating [15-17], and spawning [16,18]. Coutnho-Abreu et al. have recognized the ACP antennal neurons that responded strongly to odorants found in the host citrus plants using single-unit electrophysiology [19]. Recently, Zanadi et al.

have identified the putative ACP sex pheromone [20], in addition to other related compounds that also generated electroantennographic (EAG) responses [20-22]. Thus, to analyze the antennal morphology, structure and sensilla distribution in *D. citri* is important to explore their life cycle, olfactory behavior and host identification mechanisms.

Previous studies have described the external morphology and ultrastructure of antennal sensilla in adult *D. citri* [23], antennae sensory arrays in adult *D. citri* [24], antennal transcriptome in adult *D. citri* [25]. However, there is very little information about the morphology of nymphal antennae and antennal sensilla of psyllids. Because its ability to transmit *C.* Liberibacter was more efficient in nymphs, we investigated the antennal morphology, structure and sensilla distribution in five nymphal stages of *D. citri* using scanning electron microscopy. The results could contribute to a better understanding of the development of the sensory system, and further explore the relationship between the ability to carry and transimit *C.* Liberibacter and the feeding behavior of *D. citri* nymphs.

Materials and methods

Insects

Specimens used in the study were five different nymphal stages of *D. citri*. These insects originated from a colony maintained at Jiangxi Agriculture University at Nanchang, China (N28°45'36", E115°49'43") that was initiated from insects collected in South China Agriculture University at Guangzhou, China (N23°10'7", E113°21'27") in June 2016. The ACP were fed on *Murraya exotica* ~~seeding~~ seedlings at 27 ± 1 ºC, 70 ± 5% and 14:10 L:D photoperiod.

Scanning electron microscopy (SEM)

The whole bodies of five different nymphal stages of *D. citri* were fixed in 2.5% glutaraldehyde at 4 ºC for 24 h and post-fixed in 1% osmium tetraoxide for 2.5 h. The treatment process for the specimens including rinsing, dehydrating and drying was

according to Yang et al. [26]. Finally, the specimens were anchored on a holder using double-sided adhesive tape in different orientations, sputter-coated with gold, examined and photographed at 10 kV using an SEM (XL30, FEI, Holland and Nova Nano 430, FEI, Holland).

Statistical analysis

Classification of sensilla types in this work was based on the morphological characteristics of similar structures described by Dethier [27], Schneider [28], Moran and Brown [29], Singleton-Smith et al. [30], Bromley et al. [31], Zacharuk [10], Zacharuk and Shields [32], Mellor and Anderson [33], Kristoffersen et al. [34], Onagbola et al. [23] and Zheng et al. [35].

Statistical tests were performed with SPSS 17.0. Means were analyzed by g[illegible]ral linear model (GLM) procedure and Tukey's mean separation test. Mann-Whitney *U* test was used to compare the length and width of various stages between the long terminal hair (TH1) and short terminal hair (TH2).

Results

General morphology of antennae

A view from the ventral side of the insect body, there was a pair of antennae observed between the compound eyes in nymphal *D. citri*. The morphology of five different nymphal stages of *D. citri* antennae is illustrated in Fig 1. There were two terminal hairs at the tip of the antennae of each nymphal stage. The first-, second- and third-instar nymphal antennae were composed of two segments (Fig 1A-C), while the fourth- and fifth-instar nymphal antennae consisted of three segments (Fig 1D and 1E). There were significant differences found in the length of the antennae of each nymphal stage (Table 1). The length of the five different nymphal antennae was significantly increased with the increase of the nymphal instars.

First-instar nymph

The antennae of the first-instar nymphs were composed of two segments (Fig 1A). They were the shortest, measuring 61.85 ± 1.83 μm in length. There was no sensillum found on the first segment of the antennae (Se1). Only six antennal sensilla belonging to five morphologically different types were observed on the antennae of the first-instar nymphs. They were the TH1, TH2, sensilla trichoidea (ST), partitioned sensory organ (PSO) and cavity sensillum 1 (CvS1).

Second-instar nymph

The antennae of the second-instar nymphs were also composed of two segments (Fig 1B). They were 35.96 μm longer than the first-instar nymphal antennae (Table 1). The sensilla occurred on the first-instar nymphal antennae were also found in the second-instar nymphs. Moreover, there was ST found on the Se1 of the second-instar nymphs.

Third-instar nymph

The antennae of the third-instar nymphs were similar to those of the first- and second-instar nymphs, which were also composed of two segments (Fig 1C). The antennae of the third-instar nymphs were about 1.5 times longer than those of the second-instar nymphs (Table 1). Ten morphologically different types of sensilla were detected on the third-instar nymphal antennae. They were TH1, TH2, ST, PSO, four types of sensilla basiconica (SB1-4), CvS1 and CvS2.

Fourth-instar nymph

The antennae of the fourth-instar nymphs were composed of three parts: two basal segments and the flagellum with poorly defined subsegments (Fig 1D). The types of antennal sensilla in the fourth-instar nymphs were one less, but the total number was one more than those in the third-instar nymphs (Table 2). The SB4 and CvS2 were

missed, however, the sensilla campaniform (SCA) was found in the fourth-instar nymphs.

Fifth-instar nymph

The antennae of the fifth-instar nymphs were similar to those of the fourth-instar nymphs, which were also composed of three parts (Fig 1F). They were the longest antennae in the five different nymphal stages of *D. citri* (Table 1). The types and the total number of antennal sensilla in the fifth-instar nymphs were the most (Table 2). Eleven morphologically different types of sensilla were detected on the fifth-instar nymphal antennae. They were TH1, TH2, ST, PSO, CvS1, CvS2, SB1-4 and SCA.

Morphology and structure of sensilla

Terminal hairs

There were two bristle-like sensilla apically on the terminal antennae of the five different nymphal stages (Fig 1 and 2A). The TH1 was strong and straight with longitudinal grooves (Fig 2A). They were inserted into a big cuticular socket. The TH2 had a similar morphology to the TH1, but much shorter and stronger (Fig 2A and 2B). The length of TH1 was significantly longer than that of the TH2 in each development stage. The lengths of TH1 and TH2 in the second-instar nymphs were the shortest, measuring 13.38 ± 0.66 μm and 9.88 ± 0.33 μm, respectively. In addition, the width of TH2 in the fifth-instar nymphs was significantly bigger than that in other nymphal stages and that of the TH1 (Table 3).

Sensilla trichoidea

Sensilla trichoidea (ST) was found on the antennae of each nymphal stage of *D. citri*. They were straight hairs with a smooth surface and a big conical socket (Fig 3). Only one ST occurred on the Se1 of the second-, third- and fourth-instar nymphs. There was also only one ST positioned just below the base of the TH1 in each development

stage (Fig 3A).

Partitioned sensory organ

Partitioned sensory organ (PSO) was located on the ventral side of the last ill-defined segment of the antennae of each nymphal stage of *D. citri*. The PSO formed with an open pit and a sheet structure from which a cavity sensillum protruded (Fig 4). The inner side of the PSO was enclosed by the cuticular fringe (Fig 4B). One large PSO was found in the first-instar nymphs, two in the second- and third-instar nymphs, three in the fourth-instar nymphs and four in the fifth-instar nymphs, respectively.

Cavity sensillum

Only one cavity sensillum 1 (CvS1) was found below the base of the TH2 in each nymphal stage. They were an oval sensory cavity formed by invaginations of the antennal cuticle and contained two sensilla (Fig 5A and S1). The cuticular parts of the two sensilla sitting on the bottom of the cavity were formed as short pegs, terminating just below the antennal surface. The tips of two short pegs were flat, and one was flower-shaped.

Cavity sensillum 2 (CvS2) was only found on the ventral side of the base of the third segment of the fifth-instar nymphal antennae. Similar to the CvS1, they were a sensory cavity containing only one sensilla peg with a flower-shaped tip (Fig 5B and 5C). The cavities were rounded, and the flower-shaped tip was not flat.

Sensilla basiconica

Only one sensilla basiconica 1 (SB1) was observed on the base of the Se1 of the third-, fourth- and fifth-instar nymphs. The SB1 was fitted into a tight socket with a sharp tip and was almost vertical to the longitudinal axis of the antennae (Fig 6A). It had a smooth surface, measuring 4.58 μm in length.

Sensilla basiconica 2 (SB2) was distributed on the dorsal side of the third-,

fourth- and fifth-instar nymphal antennae. The SB2 was also fitted into a big socket elevated above the cuticle, measuring 6.35 μm in length and 1.73 μm in basal diameter. They displayed a longitudinally grooved surface with a sharp tip (Fig 6B).

Sensilla basiconica 3 (SB3), similar to the SB2, was also found on the dorsal side of the third-, fourth- and fifth-instar nymphal antennae. They were robust on the antennae, grew in a big basal socket, and measured 31.07 μm in length and 3.31 μm in basal diameter. The SB3 had multiple-pitted surfaces formed by clutter gullies, like the amygdala (Fig 6C). The number of SB3 was much more than that of the SB2 (Table 2).

Sensilla basiconica 4 (SB4) was sparsely distributed on the dorsal side of the last segment of the third- and fifth-instar nymphal antennae. Two SB4 were found in the third-instar nymphs, and one in the fifth-instar nymphs. The SB4 had a wavy line surface, like noodles (Fig 6D). They were also robust and grew in a big basal socket, measuring 12.7 μm in length and 3.86 μm in basal diameter.

Sensilla campaniform

A single sensilla campaniform (SCA) was found on the dorsal side of the Se2 of the fourth- and fifth-instar nymphs (Fig 7). The SCA had a dome-shaped sensory structure. Their ambient cuticle was protuberant but sunken in the middle (Fig 7A). In addition, there was a concave spot at the sunken structure in SCA (Fig 7B).

Discussion

~~Discursion~~

The morphology and ultrastructure of the antennae and antennal sensilla in different nymphal stages of *D. citri* were studied. The antennae of *D. citri* were composed of two segments in the first-, second- and third-instar nymphs, two basal segments and the flagellum with poorly defined subsegments in the fourth- and fifth-instar nymphs (Fig 1). The length of the antennae of each development stage was significantly increased with the increase of the instars (Table 1), as well as the types and total number of the antennal sensilla (Table 2). At any stage of the life cycle of *D. citri*, the

total number of antennal sensilla was small; moreover, their structure was rather simple. Eleven morphologically distinct types of sensilla were found to distribute on the antennae of nymphal *D. citri* in our study. The SB and CvS were not reported by Onagbola et al. [23] in adult *D. citri*, maybe they were degenerated and missed in adults. The PSO of the nymphal instars had a 'raised edges and internal shutter-like' appearance which might be replaced by the antennal rhinaria in adult *D. citri* (see S2 and S3).

The TH were analogous with those found in *Psylla pyricola* Förster [30], *Trialeurodes vaporariorum*, *Aleyrodes proletella*, *Bemisia tabaci* [33,36], *Trioza apicalis* [34], *Dialeurodes citri* [37], *Aleurodicus dispersus* [35,38]. As is the case with other types of sensilla, the terminal hairs have been differently named by various authors. For example, Singleton-Smith et al. [30] and Mellor and Anderson [33] characterized them as “chaetica sensilla”, which can be suggested to serve as a proprioreceptor perceiving antennal movement and position or have mechano-sensory functions [23,39], due to their location and presence to membranous connections between the sensillar shaft and the antennal cuticular surfaces [40].

Trichoids hairs have been reported to perform either or both mechano- and chemo-sensory function [10]. The ST with no cellular material and pore canal structures (see S4) resembled the mechanosensory hair which positioned just below the base of the TH1 in carrot psyllid [34]. In addition, the ST present in analogous locations with the AST-2 described by Onagbola et al. [23] may also suggest to have mechanosensory functions [23,34].

The PSO was first reported in *P. pyricola* and suggested chemoreceptors [30]. Their morphology, location, and number were similar in five different nymphal instars between *P. pyricola* and *D. citri*. Previous studies have reported that the descriptions of the plate organs (sensilla placodea) in aphids conformed closest to the large sensory organ (PSO) [28,41-43]. Therefore, if these were indeed plate organs that were placed in the category of chemoreceptor by Slifer [43], they could be involved in olfactory function.

The CvS was compound sensilla which consisted of a sensory cavity and sensilla

pegs, but they were only meant the sensory cavity named by Kristoffersen et al. [34] and Onagbola et al. [23] in psyllids. The difference between CvS1 and CvS2 was the number of pegs in the cavity. All of their pegs were well hidden beneath the antennal surface, and their position was far from optimal for receiving chemical stimuli. Kristoffersen et al. [34] have reported that these pegs are likely to be a necessary adaptation to prevent desiccation. Moreover, several studies have demonstrated that sensilla that are recessed from the antennal surface and located within cavities may be involved in perception of the CO_2, humidity, and temperature [44-46]. Judging by morphological features, the CvS could be responsible for the perception of any type of stimuli as described above.

The SB only occurred on the antennae of third-, fourth- and fifth-instar nymphs. Similarly, they were not reported in other psyllids, no matter nymphs or adults [23,30,34]. In our study, the SB was inserted into a big inflexible socket and exhibited different morphological surfaces (Fig 6). Judging by the morphological surface features, four subtypes of this sensillum can be identified. The SB3 and SB4 were the reported. According to Schneider [28], sensilla basiconica are the most common and important chemoreceptors found on the antennae of insects. Moreover, Altner and Prillinger [47] and Zacharuk [48] suggested that it would be impossible for sensilla with an inflexible socket to act as a mechanoreceptor.

The SCA has been found in many insects, including whiteflies [39] and psyllids [30]. On the antennae of the fourth- and fifth-instar nymphs, only one SCA was presented on the Se2. The SCA was seemed to occur in aging nymphs (the fourth-instar nymph of *A. dispersus* and the fifth-instar nymph of *P. pyricola*) [30,35] or adults (*Tetrastichus howardi* and *A. dispersus*) [38,49]. Previous studies have reported that SCA with no pores in their cuticular structure play the role of mechanoreceptors [32,40,50-54], whereas the SCA with pores was involved in gustatory function were highly susceptible to humidity [55]. Here they probably acted as mechanoreceptors, because they were few and located near the segmental joints [10,29,56,57].

According to Hung et al. [7], *C.* Liberibacter asiaticus (Las) persists in the ACP

vector but is not transovarially transmitted by the vector. Only the third-, fourth- and fifth-instar nymphs and adults can carry and transmit Las, and Las in these nym[illegible]s can transmit to the adults. In our study, the types and number of the antennal sensilla increased in the third-, fourth- and fifth-instar nymphs. Further studies are needed to determine whether the antennal sensilla in the third-, fourth- and fifth-instar nymphs (especially the SB) are related to the feeding behavior on the citrus with Las by these nymphs. In conclusion, we have provided an extensive description of the antennae and antennal sensilla in different nymphal stages of *D. citri* using SEM. This information can be a great help for revealing the developmental course of psyllid's antennae and antennal sensilla and allow us to better understand the host location mechanism in *D. citri*.

Acknowledge

The authors are grateful to Wei-Chun Li from Jiangxi Agricultural University for English revision and critical reading of the manuscript. We would like to thank Ms Xiao-Yin Hu from the Centre Laboratory of the South China Botanical Garden for her valuable assistance with the treatment for the specimens in SEM.

Reference

1. Bové JM. Huanglongbing: a destructive, newly-emerging, century-old disease of citrus. Journal of Plant Pathology. 2006; 88: 7-37.
2. Grafton-Caldwell EE, Stelinski LL, Stansly PA. Biology and management of Asian citrus psyllid, vector of the Huanglongbing pathogens. Ann Rev Entomol. 2013; 58: 413-432.
3. Halbert SE, Manjunath KL. Asian citrus psyllids (Sternorrhyncha: Psyllidae) and greening disease of citrus: a literature review and assessment of risk in Florida. Fla Entomol. 2004; 87: 330-353.
4. Ammar ED, Alessandro R, Shatters JrRG, Hall DG. Behavioral, ultrastructural and chemical studies on the honeydew and waxy secretions by nymphs and adults of

Asian citrus psyllid *Diaphorina citri* (Hemiptera: Psyllidae). PloS One. 2013; 8(6): e64938.

5. McClean PD, Schwarz RE. Greening or blotchy mottle disease of citrus. Phytophylactica. 1970; 2: 177-194.

6. Dagulo L, Danyluk MD, Spann TM, Valim MF, Goodrich-Schneider R, Sims C, et al. Chemical characterization of orange juice from trees infected with citrus greening (Huanglongbing). Journal of Food Science. 2010; 75(2): C199-C207.

7. Hung TH, Hung SC, Chen CN, Hsu MH, Su HJ. Detection by PCR of *Candidatus* Liberibacter asiaticus, the bacterium causing citrus huanglongbing in vector psyllids: application to the study of vector pathogen relationships. Plant Pathol. 2004; 53: 96-102.

8. Boina DR, Rogers ME, Wang N, Stelinski LL. Effect of pyriproxyfen, a juvenile hormone mimic, on egg hatch, nymph development, adult emergence and reproduction of the Asian citrus psyllid, *Diaphorina citri* Kuwayama. Pest Manag Sci. 2010; 66: 349-357.

9. Cen Y, Yang C, Holford P, Beattie GAC, Spooner-Hart RN, Liang G, et al. Feeding behaviour of the Asiatic citrus psyllid, *Diaphorina citri*, on healthy and hunaglongbing infected citrus. Entomol Exp Applic. 2012; 143: 13-22.

10. Zacharuk RY. Antennae and sensilla. In: Kerkut GA, Gilbert LJ, editors. Comprehensive Insect Physiology, Biochemistry, and Pharmacology, Sensory. Oxford: Pergamon; 1985. p.1-69.

11. Bland RG. Antennal sensilla of acrididae (Orthoptera) in relation to subfamily and food preference. Ann Entomol Soc Am. 1989; 82: 368-384.

12. Blaney WM, Simmonds MSJ. The chemoreceptors. In: Chapman RE, Joern A, editors. Biology of Grasshoppers. New York: Wiley; 1989. p.1-37.

13. Gullan PJ, Cranston PS. The Insects: an Outline of Entomology, third ed. London: Blackwell Publishing Ltd; 2004. p. 86-105.

14. Patt JM, Sétamou M. Responses of the Asian citrus psyllid to volatiles emitted by flushing shoots of its rutaceous host plants. Environ Entomol. 2010; 39: 618-624.

15. Moghbeli Gharaei A, Ziaaddini M, Jalali MA, Michaud JP. Sex-specific responses

of Asian citrus psyllid to volatiles of conspecific and host-plant origin. Journal of Applied Entomology. 2014; 138(7): 500-509.

16. Wenninger EJ, Hall DG. Daily timing of mating and age at reproductive matruity in *Diaphorina citri* (Hemiptera: Psyllidae). Fla Entomol. 2007; 90: 715-722.

17. Wenninger EJ, Stelinski LL, Hall DG. Behavioral evidence for a female-produced sex attractant in *Diaphorina citri* (Hemiptera: Psyllidae). Entomol Exp Appl. 2008; 128: 450-459.

18. Moran VC, Buchan PR. Oviposition by the citrus psylla, *Trioza erytreae* (Homoptera: Psyllidae), in relation of leaf hardiness. Entomol Exp Appl. 1975; 18: 96-104.

19. Coutinho-Abreu IV, McInally S, Forster L, Luck R, Ray A. Odor coding in a disease-transmitting herbivorous insect, Asian citrus psyllid. Chem Senses. 2014; 39: 539-549.

20. Zanardi OZ, Volpe HXL, Favaris AP, Silva WD, Luvizotto RAG, Magnani RF, et al. Putative sex pheromone of the Asian citrus psyllid, *Diaphorina citri*, breaks down into an attranctant. Scientific Reports. 2018; 8(1): 455.

21. George J, Robbins PS, Alessandro RT, Stelinski LL, Lapointe SL. Formic and acetic acids in degradation products of plant volatiles elicit olfactory and behavioral reponses from an insect vector. Chem Senses. 2016; 41: 325-338.

22. Zanardi OZ, Volpe HXL, Luvizotto RAG, Magnani RF, Gonzalez F, Calvo C, et al. Laboratory and field evaluation of acetic acid-based lures for male Asian citrus psyllid, *Diaphorina citri*. Scientific Reports. 2019; 9(1): 1-10.

23. Onagbola EO, Meyer WL, Boina DR, Stelinski LL. Morphological characterization of the antennal sensilla of the Asian citrus psyllid, *Diaphorina citri* Kuwayama (Hemiptera: Psyllidae), with reference to their probable functions. Micron. 2008; 39(8): 1184-1191.

24. Arras J, Hunter W, Bextine B. Comparative analysis of antennae sensory arrays in Asian citrus psyllid, *Diaphorina citri*, and potato psyllid, *Bactericera cockerelli* (Hemiptera). Southwestern Entomologist. 2012; 37(1): 1-12.

25. Wu ZZ, Zhang H, Bin SY, Chen L, Han QX, Lin JT. Antennal and abdominal

transcriptomes reveal chemosensory genes in the Asian citrus psyllid, *Diaphorina citri*. PloS One. 2016; 11(7): e0159372.

26. Yang HY, Zheng LX, Zhang ZF, Zhang Y, Wu WJ. The structure and morphologic changes of antennae of *Cyrtorhinus lividipennis* (Hemiptera: Miridae: Orthotylinae) in different instars. PloS One. 2018; 13(11): e0207551.

27. Dethier VG. The physiology of insect senses. London: Methuen and Co. Ltd; 1963.

28. Schneider D. Insect antenna. Annual Review of Entomology.1964; 9: 103-122.

29. Moran VC, Brown RP. The antennae, host plant chemoreception and probing activity of citrus psylla, *Trioza erytreae* (Del Guercio) (Homoptera: Psyllidae). Journal of the Entomological Society of Southern Africa. 1973; 36(2): 191-202.

30. Singleton-Smith J, Chang JF, Philogène JR. Morphological differences between nymphal instars and descriptions of the antennal sensory structures of the nymphs and adults of *Psylla pyricola* Foerster (Homoptera: Psyllidae). Can J Zool. 1978; 56(7): 1576-1584.

31. Bromley AK, Dunn JA, Anderson M. Ultrastructure of the antennal sensilla of aphids I. coeloconic and placoid sensilla. Cell Tissue Res. 1979; 203: 427-442.

32. Zacharuk RY, Shields VD. Sensilla of immature insects. Annual Review of Entomology. 1991; 36: 331-354.

33. Mellor HE, Anderson M. Antennal sensilla of whiteflies: *Trialeurodes vaporariorum* (Westwood), the glasshouse whitefly, *Aleyrodes proletella* (Linnaeus), the cabbage whitefly, and *Bemisia tabaci* (Gennadius), the tobacco whitefly (Homoptera: Aleyrodidae). Part 1: external morphology. International Journal of Insect Morphology and Embryology. 1995; 24(2): 133-143.

34. Kristoffersen L, Hallberg E, Wallén R, Anderbrant O. Sparse sensillar array on *Trioza apicalis* (Homoptera, Triozidae) antennae——an adaptation to high stimulus levels? Arthropod Structure and Development. 2006; 35: 85-92.

35. Zheng LX, Wu WJ, Liang GW, Fu YG. Nymphal antennae and antennal sensilla in *Aleurodicus dispersus* (Hemiptera: Aleyrodidae). Bull Entomol Res. 2014; 104: 622-630.

36. Zhang XM, Wang S, Li S, Luo C, Li YX, Zhang F. Comparison of the antennal sensilla ultrastructure of two cryptic species in *Bemisia tabaci*. PLoS One. 2015; 10(3): e0121820.
37. Zheng LX, Xiao TT, Wu WJ, Cui ZX, Fu YG. Ultra-structure observation on the antenna sensilla of adult citrus whitefly *Dialeurodes citri*. Journal of Fruit Science. 2011; 28(2): 344-347.
38. Zheng LX, Wu WJ, Fu YG. Sensilla on antenna of adult spiraling whitefly *Aleurodicus dispersus*. Chinese Bulletin of Entomology. 2010; 47(3): 525-528.
39. Ochieng SA, Park KC, Zhu JW, Baker TC. Functional morphology of antennal chemoreceptors of the parasitoid *Microplitis croceipes* (Hymenoptera: Braconidae). Arthropod Structure and Development. 2000; 29: 231-240.
40. Chapman RF, Chapman RF. The insects: structure and function, Cambridge University Press; 1998.
41. Slifer EH, Sekhon SS, Lees AD. The sense organs on the antennal flagellum of aphids (Homoptera), with special reference to the plate organs. Q J Microsc Sci. 1964; 105: 21-30.
42. Imms AD. A general textbook of entomology, ninth ed. London: Methuen and Co. Ltd; 1970.
43. Slifer EH. The structure of arthropod chemoreceptors. Annu Rev Entomol. 1970; 15: 121-142.
44. Steinbrecht RA. Arthropods: Chemo-, thermo-, and hygro-receptor. In: Bereiter-Hahn J, Matolsty AG, Richards KS, editors. Biology of the intergument, Vol. 1. Berlin: Spring-Verlag; 1984. p. 523-553.
45. Stange G, Stowe S. Carbon-dioxide sensing structures in terrestrial arthropods. Microsc Res Tech. 1999; 17: 416-427.
46. Onagbola EO, Fadamiro HY. Scanning electron microscopy studies of antennal sensilla of *Pteromalus cerealellae* (Hymenoptera: Pteromalidae). Micron. 2008; 39: 526-535.
47. Altner H, Prillinger L. Ultrastructure of invertebrate chemo-, thermo-, and hygroreceptors and its functional significance. International Review of Cytology.

1980; 67: 69-139.
48. Zacharuk RY. Ultrastructure and function of insect chemosensilla. Annu Rev Entomol. 1980; 25: 27-47.
49. Zheng YH, Zheng LX, Liao YL, Wu WJ. Sexual dimorphism in antennal morphology and sensilla ultrastructure of a pupal endoparasitoid *Tetrastichus howardi* (Olliff) (Hymenoptera: Eulophidae). Microscopy Research and Technique. 2016; 79: 374-384.
50. Slifer EH, Sekhon SS. Fine structure of the sense organs on the antennal flagellum of the honey bee, *Apis mellifera*. Linnaeus. Journal of Morphology. 1961; 109: 351-381.
51. Stort CA, Barelli N. Genetic study of olfactory structures in the antennae of two *Apis mellifera* subspecies. Journal of the Kansas Entomological Society. 1981; 54: 352.
52. Hashimoto Y. Unique features of sensilla on the antennae of Formicidae (Hymenoptera). Journal of Applied Entomology and Zoology. 1990; 25: 491-501.
53. Olson DM, Andow DA. Antennal sensilla of female *Trichogramma nubilale* (Ertle and Davis) (Hymenoptera: Trichogrammatidae) and comparisons with other parasitic Hymenoptera. International Journal of Insect Morphology & Embryology. 1993; 22: 507-520.
54. Kleineidam C, Romani R, Tautz J, Izidoro N. Ultrastructure and physiology of the CO_2 sensitive sensillum ampullaceum in the leaf-cutting ant *Atta sexdens*. Arthropod Structure & Development. 2000; 29: 43-55.
55. Dietz A, Humphreys WJ. Scanning electron microscopic studies of antennal receptors of the worker honey bee, including sensilla campaniformia. Annals of the Entomological Society of America. 1971; 64: 919-925.
56. McIver SB. Mechanoreception. In: Kerkut GA, Gilbert LI, editors. Comparative Insect Physiology, Biochemistry and Pharmacology, Pergamon Press, Oxford; 1985. p. 71-132.
57. Liang AP, Fletcher JM. Morphology of the antennal sensilla in four Australian

spittlebug species (Hemiptera: Ceropidae) with implications for phylogeny. Australian Journal of Entomology. 2002; 41: 39-44.

Supporting information

S1 Fig. Transmission electron microscopy micrograph of the transect through the cavity sensillum 1, showing the two sensilla in the cavity (arrows).

(DOCX)

S2 Fig. General morphology of antennae of adult *D. citri*. (A) SEM photograph of the dorsal view of female *D. citri* antennae, showing the scape, pedicel, and flagellum; (B) SEM photograph of the ventral view of male *D. citri* antennae. F1, F2, F3, F4, F5, F6, F7, and F8 mean the eight flagella subsegments comprised of the flagellum; AR, antennal rhinarium; TH1, the long terminal hair; TH2, the short terminal hair.

(DOCX)

S3 Fig. Antennal rhinarium (AR) in males. (A) Antennal rhinarium, showing the cuticular fringe. (B) Antennal rhinarium, showing the pores.

(DOCX)

S4 Fig. Transmission electron microscopy micrograph of the sensilla trichoidea positioned below the base of the TH1 in ~~the~~ third-instar nymphs.

(DOCX)

Table 1 Length (mean ± SE) of various nymphal stages of *D. citri* antennae.

Nymphal stages	*N*	Length (μm)
First-instar	10	61.85 ± 1.83e
Second-instar	10	97.81 ± 1.98d
Third-instar	10	150.83 ± 4.39c
Fourth-instar	10	188.38 ± 20.69b
Fifth-instar	10	316.11 ± 3.22a

Means with same letters in the same column are not significantly different (GLM, Tukey, P>0.05).

Table 2 Abundance and distribution of sensilla on the antennae of various nymphal stages of *D. citri*.

Nymphal stages		Sensilla											Total	
		ST	TH1	TH2	PSO	CvS1	CvS2	SCA	SB1	SB2	SB3	SB4		
First-instar	S1	—	—	—	—	—	—	—	—	—	—	—	—	6
	S2	2	1	1	1	1	—	—	—	—	—	—	6	
Second-instar	S1	1	—	—	—	—	—	—	—	—	—	—	1	9
	S2	3	1	1	2	1	—	—	—	—	—	—	8	
Third-instar	S1	1	—	—	—	—	—	—	1	—	—	—	2	17
	S2	3	1	1	2	1	1	—	—	1	3	2	15	
Fourth-instar	S1	1	—	—	—	—	—	1	1	—	—	—	3	18
	S2	—	—	—	—	—	—	—	—	1	1	—	2	
	S3	4	1	1	3	1	—	—	—	1	2	—	13	
Fifth-instar	S1	4	—	—	—	—	—	1	1	—	—	—	6	31
	S2	3	—	—	—	—	—	—	—	—	3	—	6	
	S3	5	1	1	4	1	1	—	—	3	2	1	19	

Number and location of the various sensilla observed on the antennae of various nymphal stages of *D. citri*. S1, S2 and S3: segment 1, 2 and 3; ST: sensilla trichoidea; TH1 and TH2: the long and short terminal hair at the tip of the antennae, respectively; PSO: partitioned sensory organ; CvS1 and CvS2: cavity sensillum 1 and 2, respectively; SCA: sensilla campaniform; SB1, SB2, SB3 and SB4: sensilla basiconica 1, 2, 3 and 4, respectively; '—', indicates absent.

Table 3 Length and width (mean ± *SE*) of TH1 and TH2 of various nymphal stages of *D. citri*.

Stages	Length (μm)		width(μm)	
	TH1	TH2	TH1	TH2
First-instar	16.03±0.62ABa	13.60±0.68ABb	2.16±0.14Ca	1.99±0.14Ca
Second-instar	13.38±0.66Ba	9.88±0.33Bb	2.37±0.20Ca	2.45±0.09Ca
Third-instar	16.34±0.85ABa	10.30±0.83Bb	2.53±0.09Ca	2.73±0.19BCa
Fourth-instar	18.87±1.33ABa	13.00±0.51ABb	2.90±0.25BCa	3.65±0.30Ba
Fifth-instar	21.89±1.32Aa	15.13±1.23Ab	4.05±0.49ABa	4.81±0.26Ab

Means in columns with same uppercase letters are not significantly different (GLM, Tukey, $P>0.05$). Means in rows with same lowercase letters are not significantly different ($P>0.05$) in Mann–Whitney *U* test.

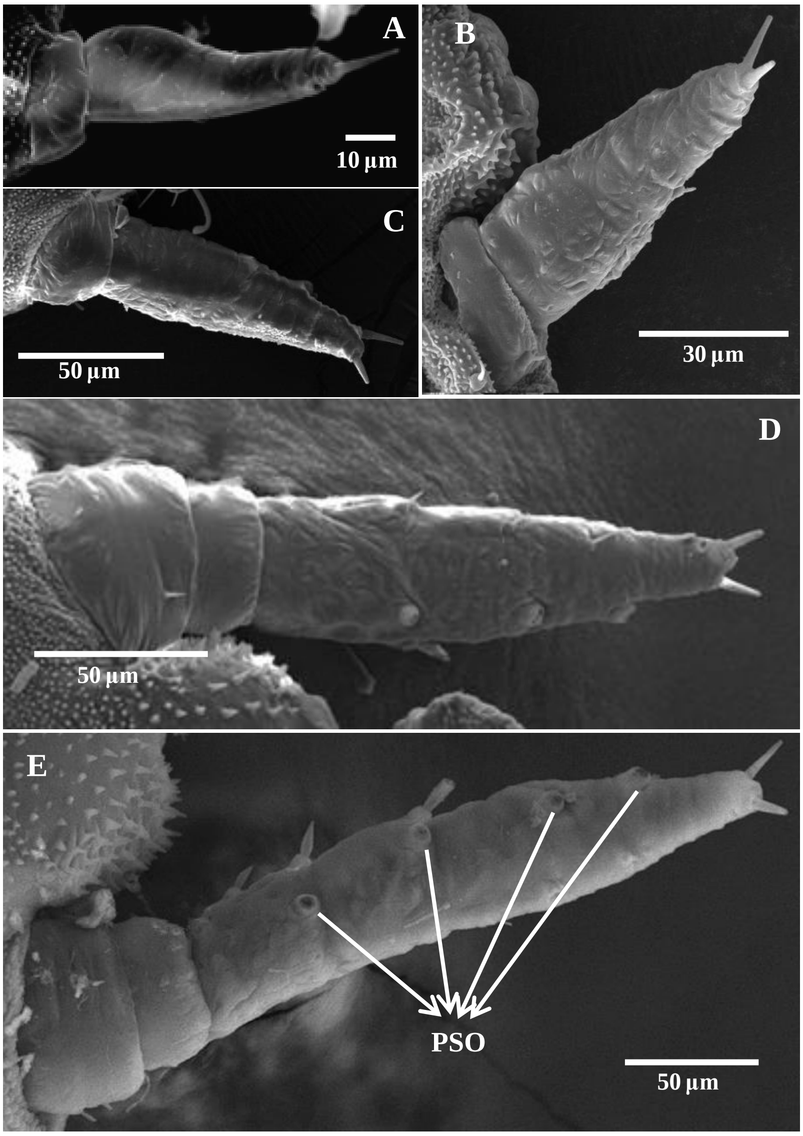

Fig 1. General morphology of antennae of immature *D. citri*. (A-E) SEM photograph in the first-, second-, third-, fourth- and fifth-instar of *D. citri* nymphal antennae. PSO, partitioned sensory organ.

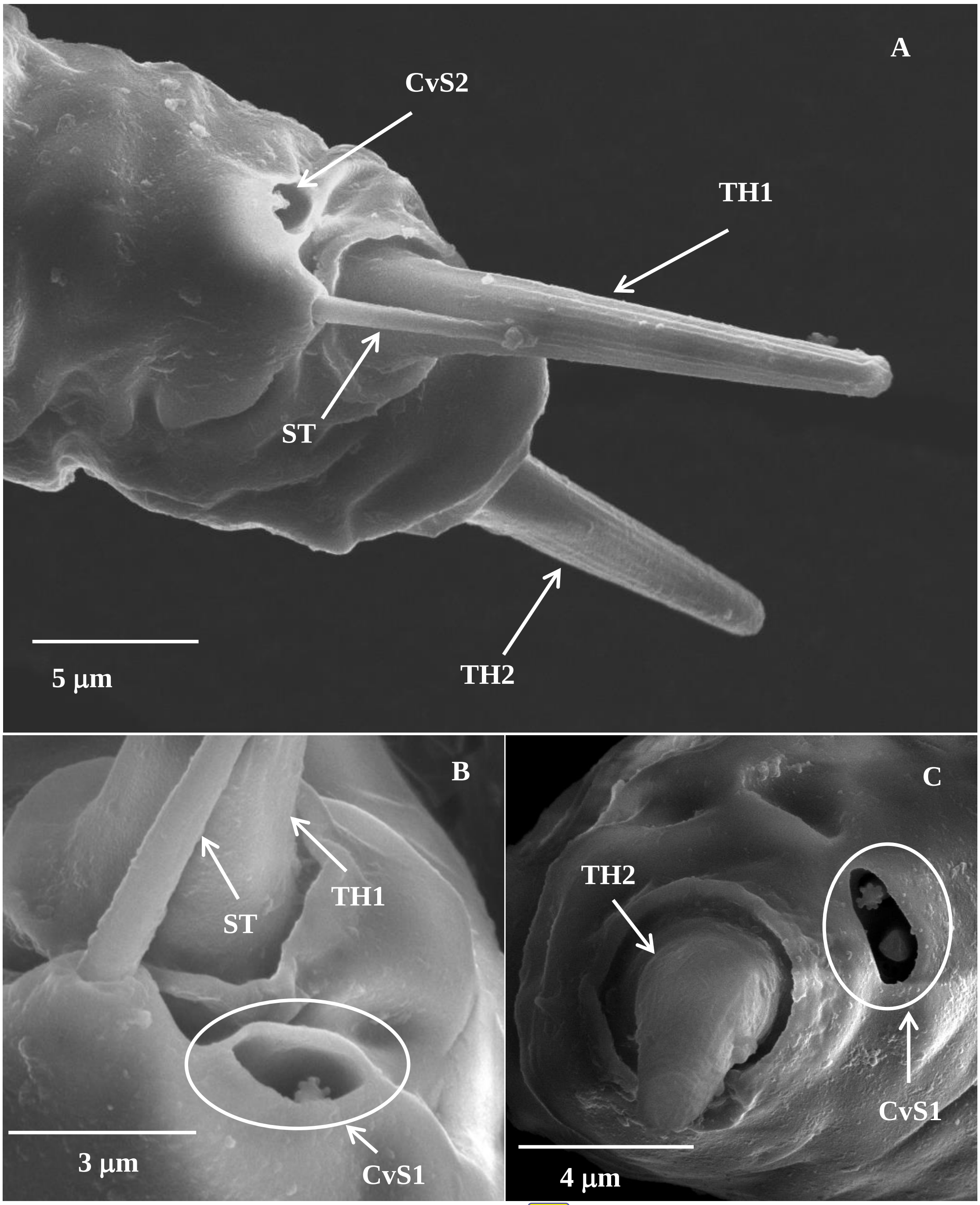

Fig 2. The antennal tip of the third-instar nymphs. (A) Two terminal hairs and sensilla trichoidea. (B) Cavity sensillum 1 and sensilla trichoidea. (C) The short terminal hair and cavity sensillum 1. TH1, the long terminal hair; TH2, the short terminal hair; ST, sensilla trichoidea; CvS1, cavity sensillum 1; CvS2, cavity sensillum.

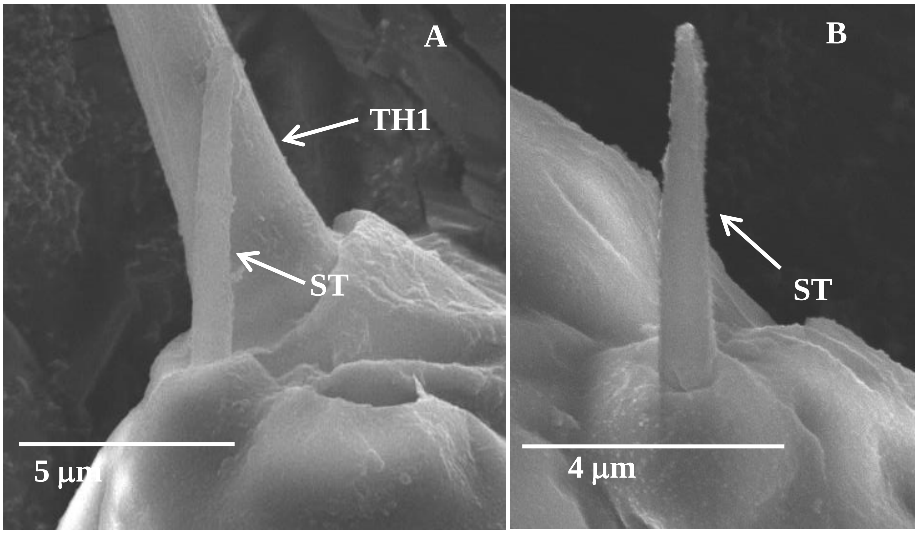

Fig 3. Sensilla trichoidea in the third-instar nymphs. (A) The long terminal hair (TH1) and sensilla trichoidea (ST). (B) Sensilla trichoidea.

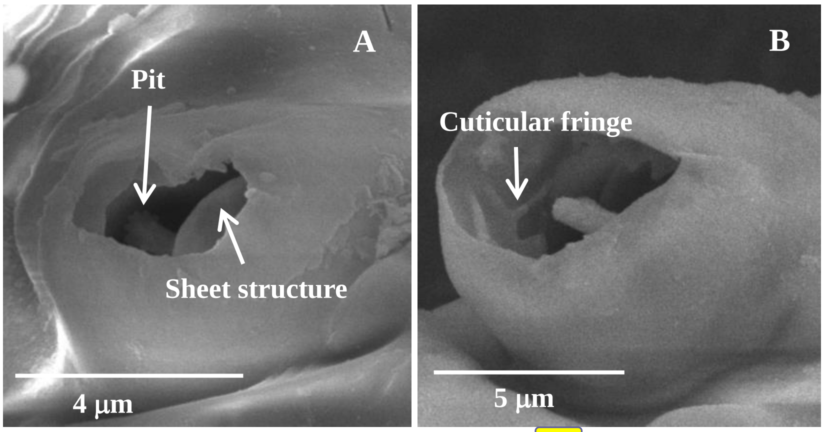

Fig 4. Partitioned sensory organ in the fifth-instar nymph. (A) Partitioned sensory organ, showing the pit and sheet structure. (B) Partitioned sensory organ, showing the cuticular fringe.

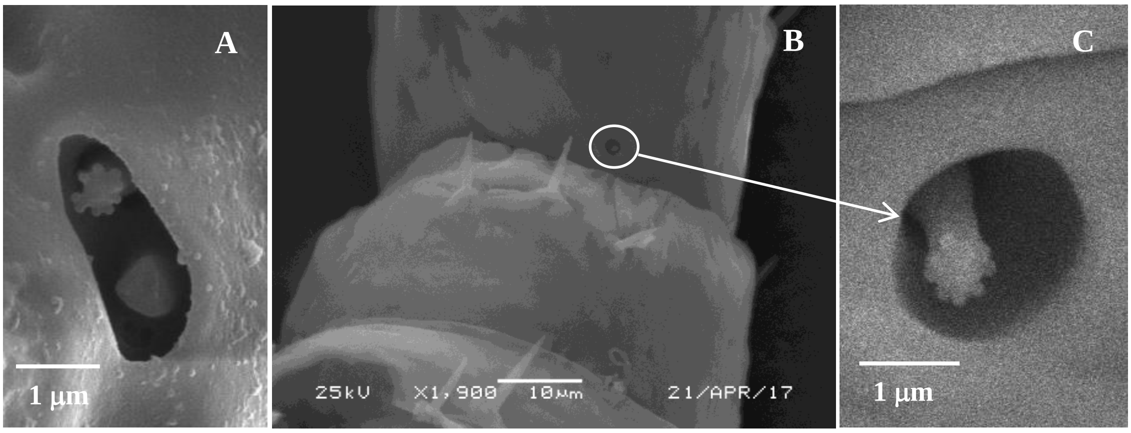

Fig 5. Cavity sensillum in the fifth-instar nymph. (A) Cavity sensillum 1. (B) Cavity sensillum 2. (C) The high magnification picture of cavity sensillum 2.

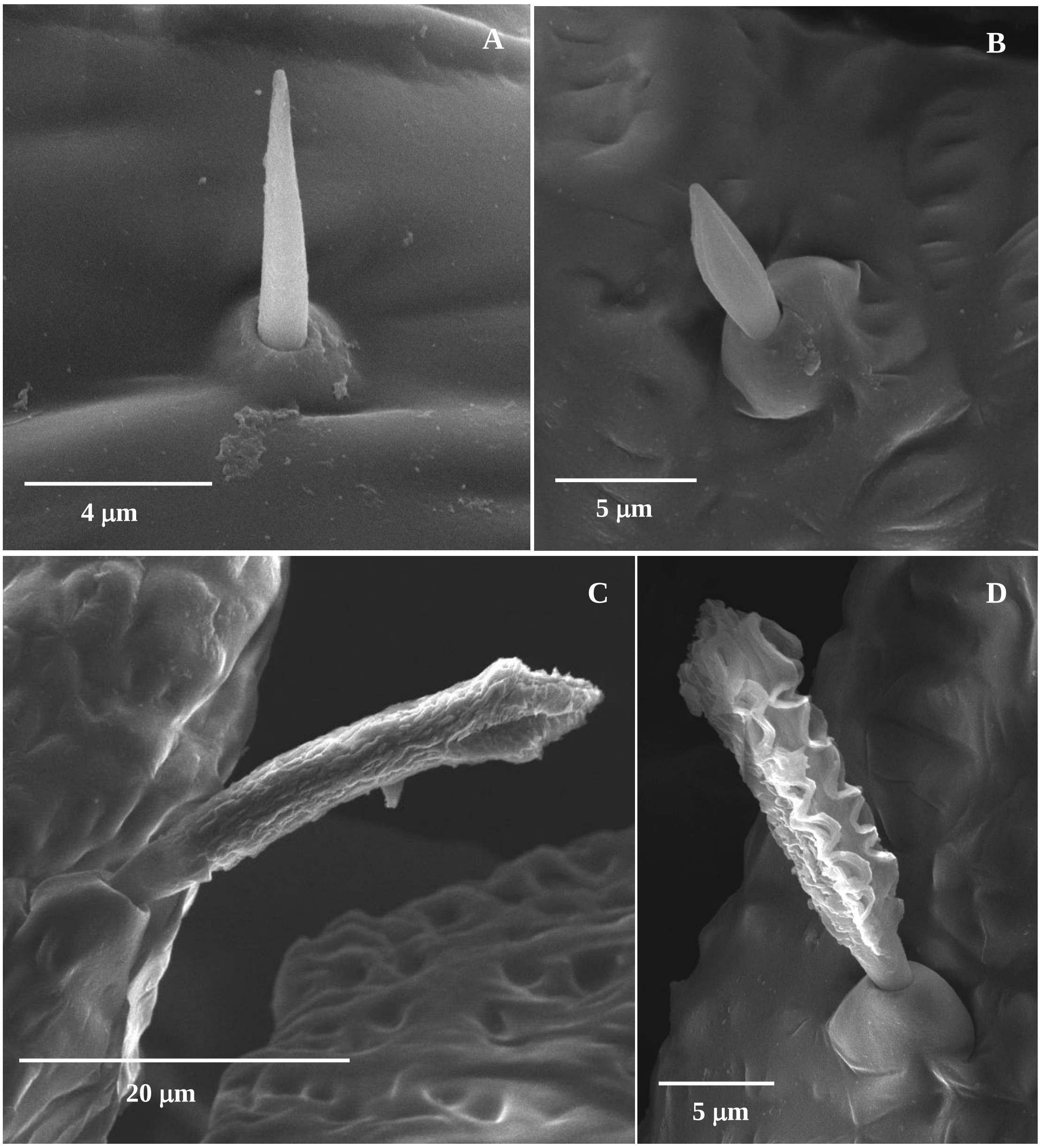

Fig 6. Sensilla basiconica in the fourth-instar nymph. (A) Sensilla basiconica 1. (B) Sensilla basiconica 2. (C) Sensilla basiconica 3. (D) Sensilla basiconica 4.

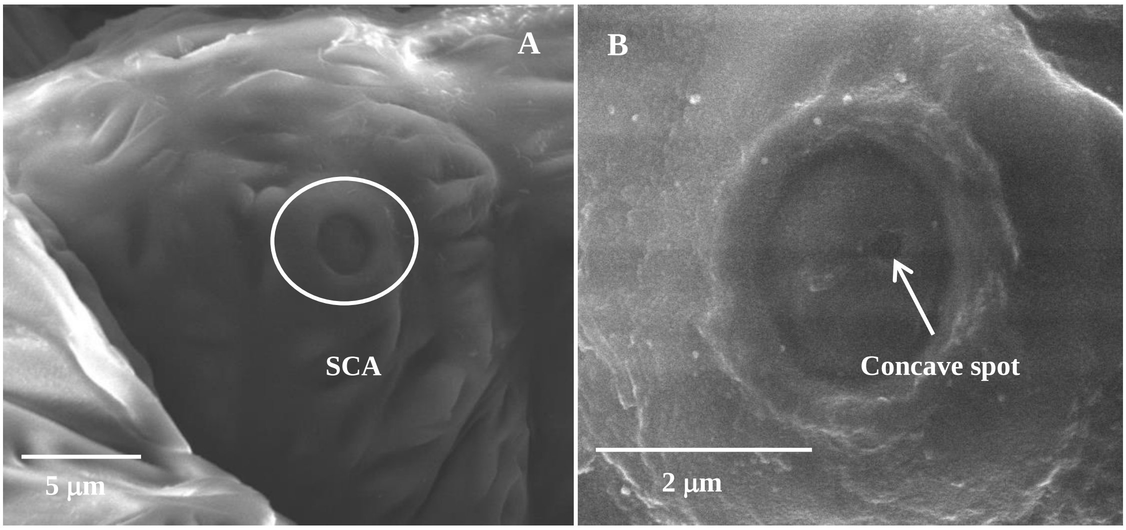

Fig 7. Sensilla campaniform (SCA) in fourth-instar nymphs. (A) Sensilla campaniform on the second segment of the antennae. (B) Sensilla campaniforn, showing the concave spot.

Click here to access/download
Supporting Information
Supplenmentary data.docx

Morphological characterization of nymphal antennae and antennal sensilla of *Diaphorina citri* Kuwayama (Hemiptera: Psyllidae)

Lixia Zheng[1,2]*, Wensheng Chen[1]

1 College of Horticulture and Food Sciences, Foshan University, Foshan, China.~~;~~ **2** College of Agronomy, Jiangxi Agricultural University, Nanchang, China.

*lxzheng@fosu.edu.cn~~86@hotmail.com~~; lxzheng@jxau.edu.cn (LZ)

Formatted: Font: Bold

Formatted: Font: Bold, Not Superscript/ Subscript

Formatted: Font: Bold

Formatted: Font: Bold

Field Code Changed

Formatted: Underline, Font color: Blue

Formatted: Font color: Blue

Abstract

Diaphorina citri Kuwayama is an economically important citrus pest which is the primary vector of *Candidatus* Liberibacter spp. causing citrus greening (huanglongbing, HLB) disease. Because its ability to transmit *C.* Liberibacter was more efficient in nymphs, we investigated the antennal morphology, structure and sensilla distribution in five nymphal stages of *D. citri* using scanning electron microscopy. The antennae of five different nymphal stages were filiform in shape, which consisted of two segments in the first-, second- and third-instar nymphs; three segments in the fourth- and fifth-instar nymphs. The length of their antennae was significantly increased with the increase of the instars, as well as the total number of antennal sensilla. Eleven morphological sensilla types were recorded altogether. They were the long terminal hair (TH1), short terminal hair (TH2), sensilla trichoidea (ST), cavity sensillum 1 (CvS1), cavity sensillum 2 (CvS2), sensilla basiconica 1-4 (SB1-4), sensilla campaniform (SCA) and partitioned sensory organ (PSO). Also, the distribution of antennal sensilla in each nymphal stage of *D. citri* was asymmetrical. The SB only occurred on the antennae of the third-, fourth- and fifth-instar nymphs. Only one CvS2 was found in the third- and fifth-instar nymphs, and one SCA in the fourth- and fifth-instar nymphs, respectively. The possible roles of the antennal

sensilla ~~of~~in *D. citri* nymphs were discussed. The results could contribute to further explore the relationship between the ability to carry and transmit *C.* Liberibacter and the feeding behavior of *D. citri* nymphs.

Introduction

The Asian citrus psyllid (ACP), *Diaphorina citri* Kuwayama (Hemiptera: Psyllidae), is an economically important citrus pest because it is the primary vector of *Candidatus* Liberibacter spp. causing citrus greening (huanglongbing, HLB) disease, the most serious citrus disease worldwide [1,2]. *D. citri* apparently originated in southern Asia, but now has~~ve~~ spread to other citrus-producing areas of the world [2]. More than 50 species of the family Rutaceae have been recorded as hosts of *D. citri* [3]. Nymphs and adults suck phloem sap from the foliage causing leaf distortion, curling~~,~~ and deposition of honeydew on leaves that result in mold growth [3,4]. However, the most serious threat to citrus worldwide comes from its ability to transmit the bacterium, *C.* Liberibacter spp., the causal organism of HLB which causes yellowing of shoots, stem dieback, sour fruit, crop losses and eventually tree death [5,6]. The nymphs and adults of *D. citri* feed on plant sap and the pathogen can only be transmitted by the third-, fourth- and fifth-instar nymphs and adults which play a major role in the spread of the pathogen because of their dispersal capabilities [2,7-9], but the nymphs were found to be more efficient [4].

Formatted: No underline, Font color: Auto

For most insects, the antennae are peripheral sensory structures involved in habitat searching, host location, host discrimination, mating and oviposition [10-12]. They carry different kinds of sensilla which can be classified as chemosensory, mechanosensory and thermo-hygroreceptive sensilla based on their morphological and functional characteristics~~.~~ [10,13]. Studies on the interaction between ~~citrus psylla~~ACP and host plant volatiles indicated the critical role of~~ a citrus psylla'~~~~s~~ its olfactory system in finding host plants [14,15], mating [15-17]~~,~~ and spawning [16,18]. Coutnho-Abreu et al. have recognized ~~identified~~ the ACP antennal neurons that responded strongly to odorants found in the host citrus plants using single-unit

electrophysiology [19]. Recently, Zanadi et al. have identified the putative ACP sex pheromone [20], in addition to other related compounds that also generated electroantennographic (EAG) responses [20-22]. Thus, to analyze the antennal morphology, structure and sensilla distribution in *D. citri* is important to explore their life cycle, olfactory behavior~~,~~ and host identification mechanisms.

Previous studies have described the external morphology and ultrastructure of antennal sensilla in~~of~~ adult *D. citri* [23~~19~~], antennae sensory arrays in adult *D. citri* [24~~0~~], antennal transcriptome in adult *D. citri* [25~~1~~]. However, there is very little information about the morphology of ~~psyllid~~ nymphal antennae and antennal sensilla of psyllids. Because its ability to transmit *C.* Liberibacter was more efficient in nymphs, we investigated the antennal morphology, structure and sensilla distribution in five nymphal stages of *D. citri* using scanning electron microscopy. The results could contribute to a better understanding of the development of the sensory system, and further explore the relationship between the ability to carry and transimit *C.* Liberibacter and the feeding behavior of *D. citri* nymphs.

Materials and methods

Insects

Specimens used in the study were five different nymphal stages of *D. citri*. These insects originated from a colony maintained at Jiangxi Agriculture University at Nanchang, China (N28°45'36", E115°49'43") that was initiated from insects collected in South China Agriculture University at Guangzhou, China (N23°10'7", E113°21'27") in June 2016. The ~~psyllids~~ ACP were fed on *Murraya exotica* seeding at 27 ± 1 ºC, 70 ± 5%~~,~~ and 14:10 L:D photoperiod.

Scanning electron microscopy (SEM)

The whole bodies of five different nymphal stages of *D. citri* were fixed in 2.5% glutaraldehyde at 4 ºC for 24 h and post-fixed in 1% osmium tetraoxide for 2.5 h. The treatment process for the specimens including rinsing, dehydrating and drying was

according to Yang et al. [26~~2~~]. Finally, the specimens were anchored on a holder using double-sided adhesive tape in different orientations, sputter-coated with gold, examined and photographed at 10 kV using an SEM (XL30, FEI, Holland and Nova Nano 430, FEI, Holland).

Statistical analysis

Classification of sensilla types in this work was based on the morphological characteristics of similar structures described by Dethier [27~~3~~], Schneider [~~24~~28], Moran and Brown [29~~5~~], Singleton-Smith et al. [30~~26~~], Bromley et al. [31~~27~~], Zacharuk [10], Zacharuk and Shields [32~~28~~], Mellor and Anderson [33~~29~~], Kristoffersen et al. [34~~0~~], Onagbola et al. [23~~19~~] and Zheng et al. [35~~1~~].

Statistical tests were performed with SPSS 17.0. Means were analyzed by general linear model (GLM) procedure and Tukey's mean separation test. Mann-Whitney *U* test was used to compare the length and width of various stages between the long terminal hair (TH1) and short terminal hair (TH2).

Results

General morphology of antennae

A view from the ventral side of the insect body, there was a pair of antennae observed between the compound eyes in nymphal *D. citri*. The morphology of five different nymphal stages of *D. citri* antennae is illustrated in Fig 1. There were two terminal hairs at the tip of the antennae of ~~in~~ each ~~of the five~~ nymphal stage~~s~~. The first-, second- and third-instar nymphal antennae were composed of two segments (Fig 1A-C), while the fourth- and fifth-instar nymphal antennae consisted of three segments (Fig 1D and 1E). There were significant differences found in the length of the antennae of each nymphal stage (Table 1). The length of the five different nymphal antennae was significantly increased with the increase of the nymphal instars.~~; the fifth-instar nymphal antennae were sure the longest.~~

First-instar nymph

The antennae of the first-instar nymphs were composed of two segments (Fig 1A). They were the shortest, measuring 61.85 ± 1.83 μm in length. There was no sensillum found on the first segment of the antennae (Se1)~~ was absent sensilla~~. Only six antennal sensilla belonging to five morphologically different types were observed on the antennae of the first-instar nymphs. They were the ~~long terminal hair (~~TH1~~)~~,~~ and short terminal hair (~~TH2~~)~~, sensilla trichoidea (ST), partitioned sensory organ (PSO) and cavity sensillum 1 (CvS1).

Second-instar nymph

The antennae of the second-instar nymphs were also composed of two segments (Fig 1B). They were 35.96 μm longer than the first-instar nymphal antennae (Table 1). The sensilla occurred on the first-instar nymphal antennae were also found in the second-instar nymphs. Moreover, there was ST found on the Se1 of the second-instar nymphs~~al antennae~~.

Third-instar nymph

The antennae of the third-instar nymphs were similar to those of the first- and second-instar nymphs, which were also composed of two segments (Fig 1C). The antennae of the third-instar nymphs were about 1.5 times longer than those of the second-instar nymphs (Table 1). Ten morphologically different types of sensilla were detected on the third-instar nymphal antennae. They were TH1, TH2, ST, PSO, four types of sensilla basiconica (SB1-4), CvS1 and CvS2.

Fourth-instar nymph

The antennae of the fourth-instar nymphs were composed of three parts: two basal segments and the flagellum with poorly defined subsegments (Fig 1D). The types of antennal sensilla in the fourth-instar nymphs were one less, but the total number was

one more than those in the third-instar nymphs (Table 2). The SB4 and CvS2 were missed~~absent~~, however, the sensilla campaniform (SCA) was found in the fourth-instar nymphs.

Fifth-instar nymph

The antennae of the fifth-instar nymphs were similar to those of the fourth-instar nymphs, which were also composed of three parts (Fig 1F). They were the longest antennae in the five different nymphal stages of *D. citri* (Table 1). The types and the total number of antennal sensilla in the fifth-instar nymphs were the most (Table 2). Eleven morphologically different types of sensilla were detected on the fifth-instar nymphal antennae. They were TH1, TH2, ST, PSO, CvS1, CvS2, SB1-4 and SCA.

Morphology and structure of sensilla

Terminal hairs

There were two bristle-like sensilla apically on the terminal antennae of the ~~in~~ five different nymphal stages (Fig 1 and 2A). The TH1 was strong and straight with longitudinal grooves (Fig 2A). They were inserted into a big cuticular socket. The TH2 had a similar morphology to~~with~~ the TH1, but much shorter and stronger (Fig 2A and 2B). The length of TH1 was significantly longer than that of the TH2 in each development stage. The lengths of TH1 and TH2 in the second-instar nymphs were the shortest, measuring 13.38 ± 0.66 μm and 9.88 ± 0.33 μm, respectively. In addition, the width of TH2~~1~~ in the fifth-instar nymphs was significantly bigger than that in other nymphal stages and that of the TH1~~2~~ (Table 3).

Sensilla trichoidea

Sensilla trichoidea (ST) was found on the antennae of each nymphal stage of *D. citri*. They were straight hairs with a smooth surface and a big conical socket~~s~~ (Fig 3). Only one ST occurred on the Se1 of the second-, third- and fourth-instar nymph~~sal antennae~~.

There was also only one ST positioned just below the base of the TH1 in~~at~~ each development stage (Fig 3A).

Partitioned sensory organ

Partitioned sensory organ (PSO) was located on the ventral side of the last ill-defined segment of the antennae of each nymphal stage of *D. citri*. The PSO formed with an open~~ed~~ pit and a sheet structure from which a cavity sensillum protruded (Fig 4). The inner side of the PSO was enclosed by the cuticular fringe (Fig 4B). One large PSO was found in the first-instar nymphs, two in the second- and third-instar nymphs, three in the fourth-instar nymphs~~,~~ and four in the fifth-instar nymphs, respectively.

Cavity sensillum

Only one cavity sensillum 1 (CvS1) was found below the base of the TH2 in~~at~~ each nymphal stage. They were an oval sensory cavity formed by invaginations of the antennal cuticle and contained two sensilla (Fig 5A and S1). The cuticular parts of the two sensilla sitting on the bottom of the cavity were formed as short pegs, terminating just below the antennal surface. The tips of two short pegs were flat, and one was flower~~-~~shaped.

Formatted: No underline, Font color: Auto

Cavity sensillum 2 (CvS2) was only found on the ventral side of the base of the third segment of the fifth-instar nymphal antennae. Similar to the CvS1, they were a sensory cavity containing only one sensilla peg with a flower~~-~~shaped tip (Fig 5B and 5C). The cavities were rounded, and the flower~~-~~shaped tip was not flat.

Formatted: No underline, Font color: Auto

Sensilla basiconica

Only one sensilla basiconica 1 (SB1) was observed on the base of the Se1 of the third-, fourth- and fifth-instar nymph~~sal antennae~~. The SB1 was fitted into a tight socket with a sharp tip and was almost vertical to the longitudinal axis of the antennae (Fig 6A). It

had a smooth surface, measuring 4.58 μm in length.

Sensilla basiconica 2 (SB2) was distributed on the dorsal side of the third-, fourth- and fifth-instar nymphal antennae. The SB2 was also fitted into a big socket elevated above the cuticle, measuring 6.35 μm in length and 1.73 μm in basal diameter. They displayed a longitudinally grooved surface with a sharp tip (Fig 6B).

Sensilla basiconica 3 (SB3), similar to the SB2, was also found on the dorsal side of the third-, fourth- and fifth-instar nymphal antennae. They were robust on the antennae, grew in a big basal socket, and measured 31.07 μm in length and 3.31 μm in basal diameter. The SB3 had multiple-pitted surfaces formed by clutter gullies, like the amygdala (Fig 6C). The number of SB3 was much more than that of the SB2 (Table 2).

Sensilla basiconica 4 (SB4) was sparsely distributed on the dorsal side of the last segment of the third- and fifth-instar nymphal antennae. Two SB4 were found in the third-instar nymphs, and one in the fifth-instar nymphs. The SB4 had a wavy line surface, like noodles (Fig 6D). They were also robust and grew in a big basal socket, measuring 12.7 μm in length and 3.86 μm in basal diameter.

Sensilla campaniform

A single sensilla campaniform (SCA) was found on the dorsal side of the Se2 of the fourth- and fifth-instar nymph~~sal antennae~~ (Fig 7). The SCA had a dome-shaped sensory structure. Their ~~which the~~ ambient cuticle was protuberant but sunken in the middle (Fig 7A). In addition, there was a concave spot at the sunken structure in SCA (Fig 7B).

Discursion

The morphology and ultrastructure of the antennae and antennal sensilla in different nymphal stages of *D. citri* were studied. The antennae of *D. citri* were composed of two segments in the first-, second- and third-instar nymphs, two basal segments and the flagellum with poorly defined subsegments in the fourth- and fifth-instar nymphs

Formatted: No underline, Font color: Auto

(Fig 1)., The length of the antennae ofin each development stage was significantly increased with the increase of the instars (Table 1), as well as the types and total number of the antennal sensilla (Table 2). At any stage in of the life cycle of *D. citri*, the total number of antennal sensilla was small; moreover, their structure was rather simple. Eleven morphologically distinct types of sensilla were found to distribute on the antennae of nymphal *D. citri* in our study. The SB and CvS were not reported by Onagbola et al. [2319] in adult *D. citri*, maybe they were degenerated and absent missed in adults. The PSO of the nymphal instars had a 'raised edges and internal shutter-like' appearance which might be replaced by the antennal rhinaria in adult *D. citri* (see S2 and S3).

The TH were analogous with those found in *Psylla pyricola* Förster [3026], *Trialeurodes vaporariorum*, *Aleyrodes proletella*, *Bemisia tabaci* [3329,362], *Trioza apicalis* [340], *Dialeurodes citri* [373], *Aleurodicus dispersus* [351,384]. As is the case with other types of sensilla, the terminal hairs have been differently named by various authors. For example, Singleton-Smith et al. [3026] and Mellor and Anderson [3329] characterized them as "chaetica sensilla", which can be suggested to serve as a proprioreceptor perceiving antennal movement and position or have mechano-sensory functions [2319,395], due to their location and presence to membranous connections between the sensillar shaft and the antennal cuticular surfaces [4036].

Trichoids hairs have been reported to perform either or both mechano- and chemo-sensory function [10]. The ST with no cellular material and pore canal structures (see S4) resembled the mechanosensory hair which positioned just below the base of the long terminal sensillum (TH1) in carrot psyllid [340]. In addition, the ST present in analogous locations with the AST-2 described by Onagbola et al. [2319] may also suggest to have mechanosensory functions [2319,340].

The PSO was first reported in *P. pyricola* and suggested chemoreceptors [3026]. Their morphology, location, and number were similar in five different nymphal instars between *P. pyricola* and *D. citri*these two psyllids. Previous studies have reported that the descriptions of the plate organs (sensilla placodea) in aphids conformed closest to the large sensory organ (PSO) [284,4137-4339]. Therefore, if these were indeed plate

Formatted: Font: Not Italic

organs that were placed in the category of chemoreceptor by Slifer [4339], they could be involved in olfactory function.

The CvS was compound sensilla which consisted of a sensory cavity and sensilla pegs, but they were only meant the sensory cavity named by Kristoffersen et al. [340] and Onagbola et al. [2319] in psyllids. The difference between CvS1 and CvS2 was the difference in the number of pegs in the cavity. All of their pegs were well hidden beneath the antennal surface, and their position was far from optimal for receiving chemical stimuli. Kristoffersen et al. [340] have reported that these pegs are likely to be a necessary adaptation to prevent desiccation. Moreover, several studies have demonstrated that sensilla that are recessed from the antennal surface and located within cavities may be involved in perception of the CO_2, humidity, and temperature [440-462]. Judging by morphological features, the CvS could be responsible for the perception of any type of stimuli as described above.

The SB only occurred on the antennae of third-, fourth- and fifth-instar nymphs. Similarly, they were not reported in other psyllids, no matter nymphs or adults [2319,3026,340]. In our study, the SB was inserted into a big inflexible socket and exhibited different morphological surfaces (Fig 6). Judging by the morphological surface features, four subtypes of this sensillum can be identified. The SB3 and SB4 were first reported. According to Schneider [284], sensilla basiconica are the most common and important chemoreceptors found on the antennae of insects. Moreover, Altner and Prillinger [473] and Zacharuk [484] suggested that it would be impossible for sensilla with an inflexible socket to act as a mechanoreceptor.

The SCA has been found in many insects, including whiteflies [395] and psyllids [3026]. On the antennae of the fourth- and fifth-instar nymphs, only one SCA was presented on the second segmentSe2 in nymphs. The SCA was seemed to occur in aging nymphs (the fourth-instar nymph of *A. dispersus* and the fifth-instar nymph of *P. pyricola*) [3026,351] or adults (*Tetrastichus howardi* and *A. dispersus*) [38,495]. Previous studies have reported that the SCA with no pores in their cuticular structure play the role of mechanoreceptors [3228,4036,5046-540], whereas the SCA with pores was involved in gustatory function were highly susceptible to humidity [551].

Formatted: Underline

Here they probably acted as mechanoreceptors, because they were few and located near the segmental joints [10,294,562,573].

According to Hung et al. [7], *C.* Liberibacter asiaticus (Las) persists in the Asian citrus psyllidACP vector but is not transovarially transmitted by the vector. Only the third-, fourth- and fifth-instar nymphs and adults can carry and transmit Las, and Las in these nymphs can transmit to the adults. In our study, the types and number of the antennal sensilla increased in the third-, fourth- and fifth-instar nymphs. Further studies are needed to determine whether the antennal sensilla in the third-, fourth- and fifth-instar nymphs (especially the SB) are related to the feeding behavior on the citrus with Las by these nymphs. In conclusion, we have provided an extensive description of the antennae and antennal sensilla in different nymphal stages of *D. citri* using SEM. This information can be a great help for revealing the developmental course of psyllid's antennae and antennal sensilla and allow us to better understand the host location mechanism in *D. citri*.

Acknowledge

The authors are grateful to Wei-Chun Li from Jiangxi Agricultural University for English revision and critical reading of the manuscript. We would like to thank Ms Xiao-Yin Hu from the Centre Laboratory of the South China Botanical Garden for her valuable assistance with the treatment for the specimens in SEM.

Reference

1. Bové JM. Huanglongbing: a destructive, newly-emerging, century-old disease of citrus. Journal of Plant Pathology. 2006; 88: 7-37.
2. Grafton-Caldwell EE, Stelinski LL, Stansly PA. Biology and management of Asian citrus psyllid, vector of the Huanglongbing pathogens. Ann Rev Entomol. 2013; 58: 413-432.
3. Halbert SE, Manjunath KL. Asian citrus psyllids (Sternorrhyncha: Psyllidae) and

greening disease of citrus: a literature review and assessment of risk in Florida. Fla Entomol. 2004; 87: 330-353.

4. Ammar ED, Alessandro R, Shatters JrRG, Hall DG. Behavioral, ultrastructural and chemical studies on the honeydew and waxy secretions by nymphs and adults of Asian citrus psyllid *Diaphorina citri* (Hemiptera: Psyllidae). PloS One. 2013; 8(6): e64938.

5. McClean PD, Schwarz RE. Greening or blotchy mottle disease of citrus. Phytophylactica. 1970; 2: 177-194.

6. Dagulo L, Danyluk MD, Spann TM, Valim MF, Goodrich-Schneider R, Sims C, et al. Chemical characterization of orange juice from trees infected with citrus greening (Huanglongbing). Journal of Food Science. 2010; 75(2): C199-C207.

7. Hung TH, Hung SC, Chen CN, Hsu MH, Su HJ. Detection by PCR of *Candidatus* Liberibacter asiaticus, the bacterium causing citrus huanglongbing in vector psyllids: application to the study of vector pathogen relationships. Plant Pathol. 2004; 53: 96-102.

8. Boina DR, Rogers ME, Wang N, Stelinski LL. Effect of pyriproxyfen, a juvenile hormone mimic, on egg hatch, nymph development, adult emergence and reproduction of the Asian citrus psyllid, *Diaphorina citri* Kuwayama. Pest Manag Sci. 2010; 66: 349-357.

9. Cen Y, Yang C, Holford P, Beattie GAC, Spooner-Hart RN, Liang G, et al. Feeding behaviour of the Asiatic citrus psyllid, *Diaphorina citri*, on healthy and hunaglongbing infected citrus. Entomol Exp Applic. 2012; 143: 13-22.

10. Zacharuk RY. Antennae and sensilla. In: Kerkut GA, Gilbert LJ, editors. Comprehensive Insect Physiology, Biochemistry, and Pharmacology, Sensory. Oxford: Pergamon; 1985. p.1-69.

11. Bland RG. Antennal sensilla of acrididae (Orthoptera) in relation to subfamily and food preference. Ann Entomol Soc Am. 1989; 82: 368-384.

12. Blaney WM, Simmonds MSJ. The chemoreceptors. In: Chapman RE, Joern A, editors. Biology of Grasshoppers. New York: Wiley; 1989. p.1-37.

13. Gullan PJ, Cranston PS. The Insects: an Outline of Entomology, third ed. London:

Blackwell Publishing Ltd; 2004. p. 86-105.

14. Patt JM, Sétamou M. Responses of the Asian citrus psyllid to volatiles emitted by flushing shoots of its rutaceous host plants. Environ Entomol. 2010; 39: 618-624.

15. Moghbeli Gharaei A, Ziaaddini M, Jalali MA, Michaud JP. Sex-specific responses of Asian citrus psyllid to volatiles of conspecific and host-plant origin. Journal of Applied Entomology. 2014; 138(7): 500-509.

16. Wenninger EJ, Hall DG. Daily timing of mating and age at reproductive matruity in *Diaphorina citri* (Hemiptera: Psyllidae). Fla Entomol. 2007; 90: 715-722.

17. Wenninger EJ, Stelinski LL, Hall DG. Behavioral evidence for a female-produced sex attractant in *Diaphorina citri* (Hemiptera: Psyllidae). Entomol Exp Appl. 2008; 128: 450-459.

18. Moran VC, Buchan PR~~.~~. Oviposition by the citrus psylla, *Trioza erytreae* (Homoptera: Psyllidae), in relation of leaf hardiness. Entomol Exp Appl. 1975; 18: 96-104.

19. Coutinho-Abreu IV, McInally S, Forster L, Luck R, Ray A. Odor coding in a disease-transmitting herbivorous insect, Asian citrus psyllid. Chem Senses. 2014; 39: 539-549.

20. Zanardi OZ, Volpe HXL, Favaris AP, Silva WD, Luvizotto RAG, Magnani RF~~,~~ ~~Esperança V, Delfino JY, Freitas R, Miranda MP, Parra JRP, Bento JMS, Leal WS~~ et al. Putative sex pheromone of the Asian citrus psyllid, *Diaphorina citri*, breaks down into an attranctant. Scientific Reports. 2018; 8(1): 455.

21. George J, Robbins PS, Alessandro RT, Stelinski LL, Lapointe SL. Formic and acetic acids in degradation products of plant volatiles elicit olfactory and behavioral reponses from an insect vector. Chem Senses. 2016; 41: 325-338.

22. Zanardi OZ, Volpe HXL, Luvizotto RAG, Magnani RF, Gonzalez F, Calvo C, et al~~,~~ ~~Oehlschlager CA, Lehan BJ, Esperança V, Delfino JY, Freitas R, Carvalho R, Mulinari TA, Miranda MP, Bento JMS, Leal WS~~. Laboratory and field evaluation of acetic acid-based lures for male Asian citrus psyllid, *Diaphorina citri*. Scientific Reports. 2019; 9(1): ~~12920~~ 1-10.

23~~19~~. Onagbola EO, Meyer WL, Boina DR, Stelinski LL. Morphological

Formatted: Indent: Left: 0", Hanging: 2 ch, First line: -2 ch

Formatted: Font: Times New Roman, No underline, Font color: Auto

Formatted: Font: Italic, No underline, Font color: Auto

Formatted: Font: Italic

characterization of the antennal sensilla of the Asian citrus psyllid, *Diaphorina citri* Kuwayama (Hemiptera: Psyllidae), with reference to their probable functions. Micron. 2008; 39(8): 1184-1191.

240. Arras J, Hunter W, Bextine B. Comparative analysis of antennae sensory arrays in Asian citrus psyllid, *Diaphorina citri*, and potato psyllid, *Bactericera cockerelli* (Hemiptera). Southwestern Entomologist. 2012; 37(1): 1-12.

251. Wu ZZ, Zhang H, Bin SY, Chen L, Han QX, Lin JT. Antennal and abdominal transcriptomes reveal chemosensory genes in the Asian citrus psyllid, *Diaphorina citri*. PloS One. 2016; 11(7): e0159372.

262. Yang HY, Zheng LX, Zhang ZF, Zhang Y, Wu WJ. The structure and morphologic–_–changes of antennae of *Cyrtorhinus lividipennis* (Hemiptera: Miridae:——Orthotylinae) in different instars. PloS One. 2018; 13(11): e0207551.

273. Dethier VG. The physiology of insect senses. London: Methuen and Co. Ltd; 1963.

284. Schneider D. Insect antenna. Annual Review of Entomology.1964; 9: 103-122.

295. Moran VC, Brown RP. The antennae, host plant chemoreception and probing activity of citrus psylla, *Trioza erytreae* (Del Guercio) (Homoptera: Psyllidae). Journal of the Entomological Society of Southern Africa. 1973; 36(2): 191-202.

3026. Singleton-Smith J, Chang JF, Philogène JR. Morphological differences between nymphal instars and descriptions of the antennal sensory structures of the nymphs and adults of *Psylla pyricola* Foerster (Homoptera: Psyllidae). Can J Zool. 1978; 56(7): 1576-1584.

3127. Bromley AK, Dunn JA, Anderson M. Ultrastructure of the antennal sensilla of aphids I. coeloconic and placoid sensilla. Cell Tissue Res. 1979; 203: 427-442.

3228. Zacharuk RY, Shields VD. Sensilla of immature insects. Annual Review of Entomology. 1991; 36: 331-354.

3329. Mellor HE, Anderson M. Antennal sensilla of whiteflies: *Trialeurodes vaporariorum* (Westwood), the glasshouse whitefly, *Aleyrodes proletella* (Linnaeus), the cabbage whitefly, and *Bemisia tabaci* (Gennadius), the tobacco

Formatted: Indent: Left: 0", Hanging: 2 ch, First line: -2 ch

whitefly (Homoptera: Aleyrodidae). Part 1: external morphology. International Journal of Insect Morphology and Embryology. 1995; 24(2): 133-143.

340. Kristoffersen L, Hallberg E, Wallén R, Anderbrant O. Sparse sensillar array on *Trioza apicalis* (Homoptera, Triozidae) antennae——an adaptation to high stimulus levels? Arthropod Structure and Development. 2006; 35: 85-92.

351. Zheng LX, Wu WJ, Liang GW, Fu YG. Nymphal antennae and antennal sensilla in *Aleurodicus dispersus* (Hemiptera: Aleyrodidae). Bull Entomol Res. 2014; 104: 622-630.

362. Zhang XM, Wang S, Li S, Luo C, Li YX, Zhang F. Comparison of the antennal sensilla ultrastructure of two cryptic species in *Bemisia tabaci*. PLoS One. 2015; 10(3): e0121820.

373. Zheng LX, Xiao TT, Wu WJ, Cui ZX, Fu YG. Ultra-structure observation on the antenna sensilla of adult citrus whitefly *Dialeurodes citri*. Journal of Fruit Science. 2011; 28(2): 344-347.

384. Zheng LX, Wu WJ, Fu YG. Sensilla on antenna of adult spiraling whitefly *Aleurodicus dispersus*. Chinese Bulletin of Entomology. 2010; 47(3): 525-528.

395. Ochieng SA, Park KC, Zhu JW, Baker TC. Functional morphology of antennal chemoreceptors of the parasitoid *Microplitis croceipes* (Hymenoptera: Braconidae). Arthropod Structure and Development. 2000; 29: 231-240.

4036. Chapman RF, Chapman RF. The insects: structure and function, Cambridge University Press; 1998.

4137. Slifer EH, Sekhon SS, Lees AD. The sense organs on the antennal flagellum of aphids (Homoptera), with special reference to the plate organs. Q J Microsc Sci. 1964; 105: 21-30.

4238. Imms AD. A general textbook of entomology, ninth ed. London: Methuen and Co. Ltd; 1970.

4339. Slifer EH. The structure of arthropod chemoreceptors. Annu Rev Entomol. 1970; 15: 121-142.

440. Steinbrecht RA. Arthropods: Chemo-, thermo-, and hygro-receptor. In: Bereiter-Hahn J, Matolsty AG, Richards KS, editors. Biology of the intergument,

Vol. 1. Berlin: Spring-Verlag; 1984. p. 523-553.

45~~1~~. Stange G, Stowe S. Carbon-dioxide sensing structures in terrestrial arthropods. Microsc Res Tech. 1999; 17: 416-427.

46~~2~~. Onagbola EO, Fadamiro HY. Scanning electron microscopy studies of antennal sensilla of *Pteromalus cerealellae* (Hymenoptera: Pteromalidae). Micron. 2008; 39: 526-535.

47~~3~~. Altner H, Prillinger L. Ultrastructure of invertebrate chemo-, thermo-, and hygroreceptors and its functional significance. International Review of Cytology. 1980; 67: 69-139.

48~~4~~. Zacharuk RY. Ultrastructure and function of insect chemosensilla. Annu Rev Entomol. 1980; 25: 27-47.

49~~5~~. Zheng YH, Zheng LX, Liao YL, Wu WJ. Sexual dimorphism in antennal morphology and sensilla ultrastructure of a pupal endoparasitoid *Tetrastichus howardi* (Olliff) (Hymenoptera: Eulophidae). Microscopy Research and Technique. 2016; 79: 374-384.

50~~46~~. Slifer EH, Sekhon SS. Fine structure of the sense organs on the antennal flagellum of the honey bee, *Apis mellifera*. Linnaeus. Journal of Morphology. 1961; 109: ~~–~~351-381.

51~~47~~. Stort CA, Barelli N. Genetic study of olfactory structures in the antennae of two *Apis mellifera* subspecies. Journal of the Kansas Entomological Society. 1981; 54: 352.

52~~48~~. Hashimoto Y. Unique features of sensilla on the antennae of Formicidae (Hymenoptera). Journal of Applied Entomology and Zoology. 1990; 25: 491-501.

53~~49~~. Olson DM, Andow DA. Antennal sensilla of female *Trichogramma nubilale* (Ertle and Davis) (Hymenoptera: Trichogrammatidae) and comparisons with other parasitic Hymenoptera. International Journal of Insect Morphology & Embryology. 1993; 22: 507-520.

54~~0~~. Kleineidam C, Romani R, Tautz J, Izidoro N. Ultrastructure and physiology of the CO_2 sensitive sensillum ampullaceum in the leaf-cutting ant *Atta sexdens*.

Arthropod Structure & Development. 2000; 29: 43-55.

55~~1~~. Dietz A, Humphreys WJ. Scanning electron microscopic studies of antennal receptors of the worker honey bee, including sensilla campaniformia. Annals of the Entomological Society of America. 1971; 64: 919-925.

56~~2~~. McIver SB. Mechanoreception. In: Kerkut GA, Gilbert LI, editors. Comparative Insect Physiology, Biochemistry and Pharmacology, Pergamon Press, Oxford; 1985. p. 71-132.

57~~3~~. Liang AP, Fletcher JM. Morphology of the antennal sensilla in four Australian spittlebug species (Hemiptera: Ceropidae) with implications for phylogeny. Australian Journal of Entomology. 2002; 41: 39-44.

Supporting information

S1 Fig. Transmission electron microscopy micrograph of the transect through the cavity sensillum 1, showing the two sensilla in the cavity (arrows).

(DOCX)

S2 Fig. General morphology of antennae of adult *D. citri*. (A) SEM photograph of the dorsal view of female *D. citri* antennae, showing the scape, pedicel, and flagellum; (B) SEM photograph of the ventral view of male *D. citri* antennae. F1, F2, F3, F4, F5, F6, F7, and F8 mean the eight flagella subsegments comprised of the flagellum; AR, antennal rhinarium; TH1, long terminal hair; TH2, short terminal hair.

(DOCX)

S3 Fig. Antennal rhinarium (AR) in males (A&B).

(DOCX)

S4 Fig. Transmission electron microscopy micrograph of the sensilla trichoidea positioned below the base of the TH1 in the third-instar nymphs.

(DOCX)

Table 1 Length (mean ± SE) of various nymphal stages of *D. citri* antennae.

**Nymphal stages**	***N***	**Length (µm)**
First-instar	10	61.85 ± 1.83e
Second-instar	10	97.81 ± 1.98d
Third-instar	10	150.83 ± 4.39c
Fourth-instar	10	188.38 ± 20.69b
Fifth-instar	10	316.11 ± 3.22a

Means with same letters in the same column are not significantly different (GLM, Tukey, *P*>0.05).

Table 2 Abundance and distribution of sensilla on the antennae of various nymphal stages of *D. citri.*

Nymphal stages		Sensilla											Total	
		ST	TH1	TH2	PSO	CvS1	CvS2	SCA	SB1	SB2	SB3	SB4		
First-instar	S1	—	—	—	—	—	—	—	—	—	—	—	—	6
	S2	2	1	1	1	1	—	—	—	—	—	—	6	
Second-instar	S1	1	—	—	—	—	—	—	—	—	—	—	1	9
	S2	3	1	1	2	1	—	—	—	—	—	—	8	
Third-instar	S1	1	—	—	—	—	—	—	1	—	—	—	2	17
	S2	3	1	1	2	1	1	—	—	1	3	2	15	
Fourth-instar	S1	1	—	—	—	—	—	1	1	—	—	—	3	18
	S2	—	—	—	—	—	—	—	—	1	1	—	2	
	S3	4	1	1	3	1	—	—	—	1	2	—	13	
Fifth-instar	S1	4	—	—	—	—	—	1	1	—	—	—	6	31
	S2	3	—	—	—	—	—	—	—	—	3	—	6	
	S3	5	1	1	4	1	1	—	—	3	2	1	19	

Number and location of the various sensilla observed on the antennae of various nymphal stages of *D. citri*. S1, S2 and S3: segment 1, 2 and 3; ST: sensilla trichoidea; TH1 and TH2: the long and short terminal hair at the tip of the antennae, respectively; PSO: partitioned sensory organ; CvS1 and CvS2: cavity sensillum 1 and 2, respectively; SCA: sensilla campaniform; SB1, SB2, SB3 and SB4: sensilla basiconica 1, 2, 3 and 4, respectively; '—', indicates absent.

Table 3 Length and width (mean ± *SE*) of TH1 and TH2 of various nymphal stages of *D. citri*.

Stages	Length (μm)		width(μm)	
	TH1	TH2	TH1	TH2
First-instar	16.03±0.62ABa	13.60±0.68ABb	2.16±0.14Ca	1.99±0.14Ca
Second-instar	13.38±0.66Ba	9.88±0.33Bb	2.37±0.20Ca	2.45±0.09Ca
Third-instar	16.34±0.85ABa	10.30±0.83Bb	2.53±0.09Ca	2.73±0.19BCa
Fourth-instar	18.87±1.33ABa	13.00±0.51ABb	2.90±0.25BCa	3.65±0.30Ba
Fifth-instar	21.89±1.32Aa	15.13±1.23Ab	4.05±0.49ABa	4.81±0.26Ab

Means in columns with same uppercase letters are not significantly different (GLM, Tukey, *P*>0.05). Means in rows with same lowercase letters are not significantly different (*P*>0.05) in Mann–Whitney *U* test.

We have studied the comments carefully and have made correction which we hope meet with approval. The main corrections in the paper and the responds to the comments are as flowing:

Responds:

1 Change into black all red letters left in the m/s.

Response: Thank you very much. We have done and checked several times.

2 send the m/s for a second cycle of editorial processing; there are many errors, such as DiscuRsion, flower shape vs. flower shaped, The PSO formed with an opened..., the first segment of the antennae was absent sensilla, citrus psylla (the official common name for *D. citri* is Asian Citrus Psyllid (ACP), Acknowledge, to cite a few (please have a thorough review).

Response: We are grateful to Professor Walter S. Leal for the nice and good comments. We are very sorry for the errors which should not be made. So, we have corrected the errors not only listed by Professor Walter S. Leal, but also revised some other errors. Revised portion can be found in the file “Revised Manuscript with Track Changes” easily.

3 the literature is outdated; to a minimum you have to cite the work of Illiano Coutinho when referring to D. citri antennal structure; our two papers on pheromones (please perform an in-depth literature search and update your references).

Response: Thank you very much for the good suggestions. In fact, we have read these two references (“Zanardi OZ, Volpe HXL, Favaris AP, Silva WD, Luvizotto RAG, Magnani RF, et al. Putative sex pheromone of the Asian citrus psyllid, *Diaphorina citri*, breaks down into an attranctant. Scientific Reports. 2018; 8(1): 455.”; “Laboratory and field evaluation of acetic acid-based lures for male Asian citrus

psyllid, *Diaphorina citri*. Scientific Reports. 2019; 9(1): 1-10.”), but we do not know how to refer. Our teams also work for the sex pheromone of the ACP. You can find these new references in the page 2 lines55-59.

4 Follow PLoS ONE instructions (Tables go in the Main Text).

Response: Done. Thank you!

5 Improve figure legends; some of them just have a title.

Response: Done. Thank you!

6 Bring relevant TEM figures to the main text.

Response: Thank you very much for the suggestion. However, the TEM figures of the five different nymphal stages are very poor. We do think they can meet the requirements of PLoS One.

We tried our best to improve the manuscript and made some changes in the manuscript. We appreciate for warm work of Professor Walter S. Leal earnestly, and hope that the correction will meet with approval.

Once again, thank you very much for your comments and suggestions.
