# Supplementary material for: Morphological characterization of antennae and antennal sensilla of Diaphorina citri Kuwayama (Hemiptera: Liviidae) nymphs
Source: PLoS One. 2020 Jun 3;15(6):e0234030. doi: 10.1371/journal.pone.0234030 (PMC7269239; doi:10.1371/journal.pone.0234030)
Supplement: Attachment — Submitted filename: PONE-D-19-23769_R2.pdf [file pone.0234030.s011.pdf]

PLOS ONE

Morphological characterization of nymphal antennae and antennal sensilla of Diaphorina citri Kuwayama (Hemiptera: Liviidae)

--Manuscript Draft--

**Manuscript Number:**	PONE-D-19-23769R2
**Article Type:**	Research Article
**Full Title:**	Morphological characterization of nymphal antennae and antennal sensilla of Diaphorina citri Kuwayama (Hemiptera: Liviidae)
**Short Title:**	Antennal sensilla ultrastructure of Diaphorina citri of various nymphal stages
**Corresponding Author:**	Li-Xia Zheng<br>Foshan University<br>Foshan, CHINA
**Keywords:**	Diaphorina citri Kuwayama; antennal sensilla; nymph; SEM; STRUCTURE; psyllid; morphology
**Abstract:**	Diaphorina citri Kuwayama is the most economically important citrus pest which is the primary vector of Candidatus Liberibacter spp. causing citrus greening (huanglongbing, HLB) disease. To better understand the developmental and structural changes of antennae and antennal sensilla in D. citri nymphs, we investigated the antennal morphology, structure and sensilla distribution in five nymphal stages of D. citri using scanning electron microscopy. The antennae of five different nymphal stages were filiform in shape , which consisted of two segments in the first-, second- and third-instar nymphs; three segments in the fourth- and fifth-instar nymphs. The length of their antennae was significantly increased with the increase of the instars, as well as the total number of antennal sensilla. Eleven morphological sensilla types were recorded altogether. They were the long terminal hair (TH1), short terminal hair (TH2), sensilla trichoidea (ST), cavity sensillum 1 (CvS1), cavity sensillum 2 (CvS2), sensilla basiconica 1-4 (SB1-4), sensilla campaniform (SCA) and partitioned sensory organ (PSO). Also, the distribution of antennal sensilla in each nymphal stage of D. citri was asymmetrical. The SB only occurred on the antennae of the third-, fourth- and fifth-instar nymphs. Only one CvS2 was found in the third- and fifth-instar nymphs, and one SCA in the fourth- and fifth-instar nymphs, respectively. The possible roles of the nymphal antennal sensilla in D. citri were discussed. The results could contribute to a better understanding of the development of the sensory system, and facilitate future studies on the antennal functions in D. citri nymphs.
**Order of Authors:**	Li-Xia Zheng
	Wen-Sheng Chen
**Response to Reviewers:**	We have studied the comments carefully and have made correction which we hope meet with approval. The main corrections in the paper and the responds to the comments are as flowing:<br><br>Responds to Reviewer 1 (file “review”):<br>Zheng et al observed the antennae of the Diaphorina citri nymph, and identified the sensilla on the antennae with scanning electron microscopy. The general morphology difference was clarified in different nymph stages. The number, distribution, ultrastructure of the sensilla (11 types) on the nymph antennae was recorded. The function of these sensilla was deduced by comparing with the sensilla of other insect species to explore their role of in Candidatus Liberibacter transmitting and D. citri nymph feeding. The results is very interesting. However, this manuscript need some improvements to publish, especially the quality of the photo. It is difficult to get useful information without clear photo showing the characteristic of the sensilla.<br><br>1in the method section of page 3 (line 82-83), why the author used the double-sided adhesive tape, but not a carbon conductive tape. In line 84, the author said they used a XL30, and Nova Nano 430 scanning electron microscopy at 10 kV. In figure 5,B, the photo labeled with 25kV.<br>Response: Thank you very much for your carefulness. We have performed the studies

Powered by Editorial Manager® and ProduXion Manager® from Aries Systems Corporation

of SEM both at South China Agriculture University and Centre Laboratory of the South China Botanical Garden several times, but the treatment method and process of the specimens are the same. Most of the SEM photos were got at South China Agriculture University using an SEM (XL30, FEI, Holland and Nova Nano 430, FEI, Holland) at 10kV, few SEM photos were got at Centre Laboratory of the South China Botanical Garden using an SEM (JSM-6360LV, Japan) at 25kV. The reason for the specimens were used the double-sibed adhesive tape, actually, we don't know why. The method of anchoring the specimens was provided by the researchers in these two electron microscope laboratories.

2in line 94, page 4, the length and width of various stages of long terminal hair and short terminal hair were determined. How they measured the length and the width. How many insects they measured. How to minimize the measurement differences due to the viewing angles.

Response: Thank you very much for your comments. The length and width (basal diameter) of the long terminal hair and short terminal hair are measured from the printed SEM images by a slide caliper. The starting and ending points of the measured data are all in the middle. Ten insects of each stage of D. citri were examined.

3I suggest to mark the segments of the antennae in figure 1. For example, it is difficult to find any sensillum on the second- instar nymph without any mark in figure 1A (line 115) and 1B (line 122).

Response: Thank you very much for your suggestion. The first and second segments of the antennae in five different nymphal stages are segmented clearly, and we have marked for the fourth-instar in Fig 1D. In the first- and second-instar nymphs, the total number of antennal sensilla is very small; moreover, they were distributed either on the ventral side or on the dorsal of the antennae. So, we cannot observe all of them in one picture.

4All the photos are blurry (especially the photo taken by Nova Nano 430) and it is recommended to shoot again. A good result maybe obtain by Nova Nano 430 at low voltage (for example 5Kv) without dehydrating and drying of the insect.

Response: Thank you very much for your suggestion. In order to get the high quality photos, we performed our SEM study both at South China Agriculture University and Centre Laboratory of the South China Botanical Garden several times. We even tried to take the specimens without any treatments to anchor for examining by SEM. Here, we hope our photos may meet the requirement of PLoS One.

5What is the difference between terminal hair and TH1 and TH2 (in paragraph 2, page 6). How to determine the TH1 is longer than TH2, rather than the visual illusion caused by view angle.

Response: Thank you very much for your comments. TH1 was distributed with a ST on the antennal tip of different instars of D. citri (see Fig 3), including adult females and males (see Fig 1 and Fig 2A in S1 File). In D. citri nymphs, we don't find the morphological difference between TH1 and TH2 except the length and basal diameter (width). However, there is the obvious difference found in adults (see Fig 3 in S1 File). Moreover, the length of TH1 and TH2 was significant difference in the length and width (see Table 2 in S1 File).

6What is the last ill-defined segment and partitioned sensory organ? (line 171,page 7). A concise legend is needed for figure 4 to help it stand alone (useful information can be found here, https://blog.bioturing.com/2018/05/10/how-to-craft-a-figure-legend-for-scientific-papers/)

Response: Thank you very much for your comments. Partitioned sensory organ was located on the last segment of the antennae of each ACP nymphal stage. The fourth- and fifth-instar nymphs had an ill-defined flagellum which was also the last segment of the antennae. Our expression is not very accurate, and we have revised it. Also, we have checked all of our figure legends, and thank you very much.

7What is the cavity sensillum, where it come from?

Response: Thank you very much for your comment. Cavity sensillum consisted of a sensory cavity and one (Fig 5B) or two pegs (Fig 5A). I have marked the cavity sensillum with a circle, maybe it can be easy to understand. I am very sorry that I don't really understand that "where it come from?" All sensilla in our study were distributed on the antennae of five different nymphal stages of D. citri.

Powered by Editorial Manager® and ProduXion Manager® from Aries Systems Corporation

8In the discussion section, paragraph 2, the author claimed that another name of TH is chaetica sensilla, why?
Response: In most of whiteflies and psyllids such as Trialeurodes vaporariorum, Aleyrodes proletella, Bemisia tabaci, Dialeurodes citri, Aleurodicus disperses, Psylla pyricola, Trioza apicalis and D. citri, there are one or two TH found on the antennal tip. According to Singleton-Smith et al. (1978) and Mellor and Anderson (1995), they are characterized as “chaetica sensilla”, maybe due to their location and the morphology of that they are obvious strong and long with longitudinal groove surface and inserted into a big cuticular socket. These antennal sensilla should be the first to touch and perceive the external environment because of their location and size.

(Singleton-Smith J, Chang JF, Philogène JR. Morphological differences between nymphal instars and descriptions of the antennal sensory structures of the nymphs and adults of Psylla pyricola Foerster (Homoptera: Psyllidae). Can J Zool. 1978; 56(7): 1576-1584.)
(Mellor HE, Anderson M. Antennal sensilla of whiteflies: Trialeurodes vaporariorum (Westwood), the glasshouse whitefly, Aleyrodes proletella (Linnaeus), the cabbage whitefly, and Bemisia tabaci (Gennadius), the tobacco whitefly (Homoptera: Aleyrodidae). Part 1: external morphology. International Journal of Insect Morphology and Embryology. 1995; 24(2): 133-143.)

9The reference format should be checked carefully, and keep them in consistent. 2,3, 5,17, 18,,24,37,39,41 44,
Response: Done, thank you very much.
Special thanks to you for your comments and suggestions.

Responds to Reviewer 2 (from the decision letter):
1 The manuscript suffers from the lack of an appropriate structural origination. Specifically, in the results section, while the first part focuses mainly on the nymphal instars the authors also deal solely with the presence (or absence) and distribution of various types of the sensilla without first presenting their characteristics. This definitely confuses the readers. I’d suggest the authors rewrite the results section by combining the second part of the results i.e. “Morphology and Structure of Sensilla” with the first part so that in each paragraph the description of each sensilla type should be followed by its presence (or absence), number, distribution, etc., in each nymphal stage. The authors are also encouraged to better formulate their hypothesis in the introduction (the information is there but is not very well promoted!).
Response: Thank you very much for your suggestion. We tried to revise our MS according to your suggestion, but we found a little messy after adjusting the part of ‘Result’ of MS layout. We have revised our MS carefully according to the comments, changed the argument used to justify the choice for nymphal stages of D. citri, and added some description to improve the structure and quality of our MS, such as lines 147-153.

2 Lack of TEM and/or electrophysiological studies: Usually studies of this kind are accompanied by TEM and/or electrophysiological experiments for strengthening the results. I understand that due to, e.g. lack of equipment or budget constraints, it might not be feasible to conduct the above-mentioned experiments. Hence, I’d suggest that …
Response: Thank you very much for your good suggestion, we also try to get some high quality TEM photographs. From March 2017 to September 2018, we have performed the studies of SEM and TEM at South China Agriculture University and Centre Laboratory of the South China Botanical Garden, moreover, at Institute of Food Science and Technology CAAS for TEM. Unfortunately, we did not get good TEM photographs of different nymphal stages of Diaphorina citri. For the electrophysiological studies of D. citri nymphs, we have not plan to do, but we have done the adult electrophysiological studies. ACP is a small insect, and it is not easy to connect the antennae in the adult electrophysiological studies. Moreover, the various nymphal stages of D. citri antennae are much shorter than the adults (see Table 1 in S1 File), especially the first-, second-, third- and fourth-instar nymphal antennae (Table 1). Thank you very much for your suggestion, it is very helpful for our future researches.

Powered by Editorial Manager® and ProduXion Manager® from Aries Systems Corporation

3 … the authors submit their findings to other journals (such as Micron) that publish these kinds of works so as to attract more readers interested in the SEM.
Response: Thank you very much for your suggestion, most of our teams' works about the SEM or TEM have published in Micron, Microscopy Research and Technique, as well as Plos One. ACP is the most important insect, and there are also quite a few researches about the ACP published in Plos One. We also think it is appropriate to submit our MS to Plos One.

4 I believe the following items will increase the value of the manuscript: 1) Inclusion of parameters like the size of individual flagellomeres across the nymphal instars. 2) Figuring out if there is a correlation between the nymph size within each nymphal stage and the number of sensilla? 3) Were the individuals size-matched? I'd suggest the authors include the average head-width (indicative of size of the nymphs) of each nymphal stage in table 1.
Response: 1) Thank you very much for your suggestions. It is very valuable and helpful for the important guiding significance to our future researches. 2) The length of the five different nymphal antennae was significantly increased with the increase of the nymphal instars, as well as the number of sensilla. We do not find the correlation between the nymph size within each nymphal stage and the number of sensilla. 3) Yes, it is size-matched. The classification characteristics of different stages of ACP nymphs are according to Ruan et al. (2012). The SEM photographs were just only antennae, not containing the whole head, so, it could not measure the ACP head-width of each nymphal stage. Thank you very much for your suggestions.
(Ruan CQ, Chen JL, Liu B, Duan YP, Xia YL. 2012. Morphology and behavior of Asian citrus psyllid, Diaphorina citri Kuwayama. Chinese Agricultural Science Bulletin, 28(31): 186-190)

5 Lines 14-15: "Because its ability to transmit C. Liberibacter was more efficient in nymphs" please change it to "Because the nymphal instars have been reported to more efficiently transmit C. Liberibacter".
Response: Done, thank you very much.

6 Line 25: "The distribution of antennal sensilla in each nymphal stage of D. citri was asymmetrical". Do you mean they are "randomly distributed"?
Response: No, it just means that the distribution of antennal sensilla in nymphs was not the same. The SBs, CvS2 and SCA are the examples of how the antennal sensilla in nymphs asymmetrically distribute. These sensilla are not distributed in each nymphal stage, only in the older nymphs such as third-, fourth- and fifth-instar.

7 Lines 93-97: Under statistical analysis, instead of clearly explaining how they analyzed their data, the authors refer their readers to 11 references that focus on the classification of the sensilla. Please remove the author names and instead write, "…similar structures described previously [27-35].
Response: Done, thank you very much.

8 When the antennae are mounted only one side of the antennae is facing upward. It is not clear how the number of different sensilla were counted. Please elaborate on that.
Response: Done, thank you very much. Each side of the antennae (dorsal, ventral, and two lateral surfaces of different nymphal stages of D. citri) was mounted to obverse. Each measured parameters was ten, and we have added the relevant contents in lines 90-92.

9 Figure legends must be self-explanatory. For example in figure 1 it must be clearly explained that A-C belong to the 1st through the 3rd nymphal instars with two antennal flagellomeres and D-E belongs to the 4th and 5th nymphal with three flagellomeres.
Response: Done, thank you very much.

10 Lines 139-140: basal segments and subsegments must be indicated in the figure.
Response: Done, thank you very much.

11 "Se 1" or "S1"? Please be consistent.
Response: I am very sorry for using such similar abbreviation. Actually, "Se1" is the abbreviation of the first segment of antennae, while "S1" is the abbreviation of the "S1

Fig." in the file "Supplementary data". We have renamed the supplementary.

12 Line 244: please change "trichoids hairs" to "trichoid sensilla"
Response: Done, thank you very much.
Special thanks to you for your comments and suggestions.

Responds to Reviewer 3 (from the decision letter):
1 Data partialy supports discussion and conclusions: the authors are unable to explain the absence of some sensillas in adults, since they did not assess adult antenna structures.
Response: Thank you very much. Actually, we have performed the studies of adult D. citri with SEM and TEM. When we sent our first MS to same professors which have rich experience in SEM and TEM, they also advised us to delete the research on the adult D. citri because of the similar research reporting by Onagbola et al. (2008), We agree with the opinion, and keep some adult results in the part of 'Discussion' as supporting information (S1 File and S1 Table,). In adults, eight morphologically distinct types of sensilla were found to distribute on the antennae of female and male D. citri in our study (S1 File), with seven types reminiscent of those described by Onagbola et al. (2008). The aporous sensilla trichoidea occurred on the medial portions of the scape, pedicel and flagellomeres 1, 2, 4 and 5, the chaetica sensilla and the unidentified uniporous sensilla which were reported by Onagbola et al. (2008) were absent while the SCA were found in our study (S1 File). In our study, we do not descript the morphology and structure of antennae and antennal sensilla of adult D. citri.

(Onagbola EO, Meyer WL, Boina DR, Stelinski LL. Morphological characterization of the antennal sensilla of the Asian citrus psyllid, Diaphorina citri Kuwayama (Hemiptera: Psyllidae) with reference to their probable functions. Micron. 2008; 39(8): 1184-1191).

2 I am not sure about the use/choice (parametrical/non-parametrical analyses) because the authors did not mention if data normality was tested, neither number of replicates.
Response: Thank you very much. For the statistical analysis, we consulted an expert (Prof. Wei-Jian Wu from South China Agricultural University who are also major in Biostatistics) and we also added the relevant contents in lines 90-92.

3 There are some mispelling in the text (comments and corrections in the pdf file)
Response: Thank you very much for your comments, we have corrected according to your kind comments, and you can find the one to one response to the pdf file bellow.

4 The species family is wrong. Liviidae instead Psyllidae
Response: Done. Thank you very much.

Responds to Reviewer 3 from the PDF file (PONE-D-19-23769_R1-reviewer):
1 The species was re-classified as Liviidae.
Response: Done. Thank you very much.

2 Line 12, "the most" instead of "an".
Response: Done. Thank you very much.

3 Please re-phrase the sentence: The literature supports that nymphs were more efficient to transimit Ca. Liberibacter to the host plant. Maybe the fact can be related to feeding behavior aspects. However, nymphs presents capacity to move. On the other hands, adults can move from host plant to another one, transmiting the bacterium to health plants. Other aspect is related to chemical behavior. Initially, adults prefers Ca. Liberibacter plants (high MeSa emittion) and can acquire the bacterium, however, after some days, the adults move to health plants (better nutritional qualities). So, I am not sure that nymphs show a better ability to transmit when compared to adults.
Response: Done. Thank you very much. As reviewers' comments and suggestions, it is inappropriate to justify the choice for immature stages. We have deleted the sentences, and added some discussion about these issues in the "Discussion" section.

4 I am not sure about the relation of the 'ability' of nymphs to transmit the bacterium (feed aspects) with the choice of nymphal antenna to perform the experiments. Since antenna is related with oddorants, and all behavioral studies with ACP assessed adults

behavior and role of compounds, why the authors excluded adult antenna? This study is very welcome to complement ACP behavioral studies.
Response: Thank you very much for your kind suggestion, it is very valuable and helpful for the important guiding significance to our future researches. We have changed the sentence according to the comments. Actually, we also finished the study of adult D. citri, but they were not written in our MS because of the similar research reporting by Onagbola et al. (2008). So, we decide to present our adult ACP results as supplementary data in S1 File and S1 Table.

(Onagbola EO, Meyer WL, Boina DR, Stelinski LL. Morphological characterization of the antennal sensilla of the Asian citrus psyllid, Diaphorina citri Kuwayama (Hemiptera: Psyllidae), with reference to their probable functions. Micron. 2008; 39(8): 1184-1191.)

5 It is not clear for me what you would like to tell about number of antennae segments. There are 3 segments in insect antennae (scape, pedicel and flagellum). Please clarify what you mean with ‘structures’.
Response: Thank you very much for your comment. Yes, there are 3 segments in adult insect antennae (scape, pedicel and flagellum) generally. However, in some low-aged development nymphal stages, such as Aleurodicus dispersus and D. citri, their flagellum has not yet developed. In our study, we observed two segments of the antennae in first-, second- and third-instar of D. citri nymphs. In other words, their flagellum doesn’t develop in these ACP nymphs.

6 How many SBs for the other instar? Please clarify? Same for CvS2 and SCA. Please, you need to be clear about the number of sensilla (and sensilla types) that differ among the instars.
Response: Thank you very much for your suggestion. The details of SBs, CvSs and SCA are elaborated in the “Result” section, the number, abundance and distribution of sensilla are listed in Table 2. We don’t list the detailed information in the “Abstract” section, maybe the summarizing information is ok.

7 Lines 29-31: Are you sure that antennae sensilla is related with feeding behavior and ability to carry and transmit Ca Liberibacter? Or is related with volatile perception? Maybe feeding behavior and bacteria acquisition and transmission is related with feeding behavior, interaction with gut and other microorganisms, please justify the relation.
Response: We are very sorry for putting forward the argumentative and uncertain conclusion. Thank you very much for your comments, and we have revised the expression.

8 Line 35, add “also”.
Response: Done. Thank you very much.

9 Use the complete name (Diaphorina citri) when start a phase.
Response: Done. Thank you very much.

10Line 43: associated with (note: there is no Koch’s postulate for these bacteria complex).
Response: Done. Thank you very much.

11Lines 47-48: Please, there is a controversy for this subject. Please clarify.
Response: Done. Thank you very much for your comment. We have deleted the sentence.

12Lines 49-50: You should link gustatory function of antennae with feeding behavior and infective insects in order to justify your theory. I am not convict about the relationship of antennae sensilla and HLB transmission. And why you choose nymphs instead adults (or both), since adults are responsible for.
Response: Thank you very much for your comment. We agree with you, there isn’t an exactly relationship of antennae sensilla and HLB transmission. Actually, we also finished the study of adult D. citri, but they were not written in our MS because of the similar research reporting by Onagbola et al. (2008).

(Onagbola EO, Meyer WL, Boina DR, Stelinski LL. Morphological characterization of

Powered by Editorial Manager® and ProduXion Manager® from Aries Systems Corporation

the antennal sensilla of the Asian citrus psyllid, Diaphorina citri Kuwayama (Hemiptera: Psyllidae), with reference to their probable functions. Micron. 2008; 39(8): 1184-1191.)

13Line 66: Perfect. But I am not convicted about the explanation used to justify the nymphs studies. The absence of studies is ok, but I do not agree with ability of transmission vs feeding behavior vs antenna sensilla in nymphs. The introduction needs to be completely written in order to justify the above comment.
Response: Thank you very much, and we have revised the relevant content according to the comment.

14Line 80: What is the rearing procedure adopted (cite authors). How many generations under laboratory conditions before the experiments? Please clarify the criteria adopted for instar characterization (figure of each instar) and morphological aspects (body length and width, wing buds aspects…days after egg hatch for each nymphal stage…
Response: Thank you very much for your comment. We are very sorry that we don't record the ACP generations after rearing under laboratory conditions before the experiments. Rearing under laboratory conditions, it usually takes about 15-20 days for ACP to finish one generation. So, it may be 14-18 generations when we begin to take the experiments in March 2017. We refer Ruan et al. (2012) to clarify criteria of ACP instar characterization. Besides the difference of body size between different stages of nymphs, the most important instar characterization is the difference of wing buds. There is no wing buds in the first-instar ACP nymphs. The wing buds are found on the second segment of the thorax in the second-instar ACP nymphs. The wing buds in the third-instar nymphs increase significantly. Their leading edges extend to the posterior edges of their compound eyes, while their posterior edges extend to the 3rd segment of the abdomen. Moreover, their antennal tips turn black and appear caudate beard. In fourth-instar ACP nymphs, the leading edges of wing buds exceed their compound eyes, the posterior edges exceed the 3rd segment of the abdomen. The fifth-instar ACP nymphs are significantly bigger than the other stage nymphs. Their antennae are black except the scape, and the TH1 and TH2 can be clearly observed.

Figure 1 The egg and five different nymphal stages of Diaphorina citri under 50 × magnification. (a) Egg; (b) First-instar nymphs; (c) Second-instar nymphs; (d) Third-instar nymphs; (e) Fourth-instar nymphs; (f) Fifth-instar nymphs.

(Ruan CQ, Chen JL, Liu B, Duan YP, Xia YL. 2012. Morphology and behavior of Asian citrus psyllid, Diaphorina citri Kuwayama. Chinese Agricultural Science Bulletin, 28(31): 186-190.)

15number of replications.
Response: Done. Thank you very much.
16Why parametrical and non-parametrical testes were adopted for different parameters? Please clarify the criteria (data normality…).
Response: Thank you very much. For the statistical analysis, we consulted an expert Prof. Wei-Jian Wu from South China Agricultural University who are also major in Biostatistics. The lengths and widths are conformed to normal distribution, the non-parametrical testes is ok.

17Line 105: Seems obvious: a pair of antennae is a characteristic of Insecta. This sentence is not helpful.
Response: Thank you very much. We have revised the sentence, you can find in lines 99-100.

18Lines 221-230: the authors replicated information presented in topic Results.
Response: Thank you very much for your comment. The first paragraph of "Discussion" section, it is the generalization and summarization of the whole results in our study. It may be some replicated information presented in "Results" section.

19Line 231: This information could be clarified if authors decided to include adults? SB and CvS have degenerated our Onagbola did not see these structures? Can you support the degeneration theory?
Response: Thank you very much for your comment. We found SBs and CvSs in ACP nymphs but not in adults; they were not reported by Onagbola et al. (2008) also. We

have added the general analysis of adult antennae and antennal sensilla in S1 File.

20Line 232: same comment. The authors are assuming the fact, but the fact is not proved. I suggest the addition of study with adults in order to conclude the degeneration/replacement by antennal rhinaria.
Response: Thank you very much for your comment. We add the main results of adult ACP in S1 File.

21Lines 235-237: Provide (Order: Family) for the listed species.
Response: Done. Thank you very much.

22Line 289: Point 1: However, there is an interaction between the bacteria, nutritional medium (presented in insect gut) and the gut that can explain the ability of third nymphs to adults to carry and transimit the bacteria. Point 2: there is a minimum period (feeding period for the nymph to acquire the bacteria. Point 3: there is a latent period of the bacteria before multiplication. Point 4: Thus, that is a minimum concentration of Ca Lbieribacter bacterial title to be detected by Qpcr. Point 5: first to second instar nymphs feed lower than higher nymphal stages and adults, ingesting lower concentration of bacteria (+latent period) + minimum concentration of bacterial title is necessary for qPCR detection could explain this phenomena. I am not convicted about the relationship between sensilla number/types with HLB aquistition/transition by older nymphal stages.
Response: Thank you very much for your comments. They are very valuable and helpful for the important guiding significance to improve our MS and to our future researches. We agree with your opinion, many works must be done to convict about the relationship between sensilla number/types with HLB aquistition/transition by older nymphal stages. We have revised the “Introduction” and “Discussion” section for the expression about the deduction combined with the literatures and the reviewers’ comments.

23The figure should be self-explained. Add insect species and stage (instar).
Response: Done. Thank you very much.

24Diaphorina citri instead D. citri.
Response: Done. Thank you very much.

25Line 526: Diaphorina citri instead D. Scientific tables: please use only vertical.
Response: Done. Thank you very much. The layout of tables is used according to the requirement in PLoS ONE. The horizontal line can be used in table.

26Figures: Diaporina citri. Inform instar that you used to take the photos.
Response: Done. Thank you very much.
Special thanks to you for your comments and suggestions.

Responds to the “Reviewer general comments” file:
1 Title: Asian citrus psyllid family: Liviidae instead Psyllidae
Response: Done, thank you very much.

2 The authors support the study of morphological characteristics of D. citri nymphs based on the fact that immature stages have the ability to transmit Ca. Liberibcter more efficiently than adults. This argument is used to justify the choice for immature stages. However, we cannot forget that adults can spread bacteria more efficiently due its flight (from plant to plant). Another aspect that supports the HLB spread is the fact that D. citri adults, at first, prefers HLB plants (regulated by MeSa Vocs) and after feeding on HLB plants, move the health plants (better nutritional aspects). I am not sure about the author´s sentence to justify the studies only with nymphs.
Response: Thank you very much for your good suggestion, we are extremely grateful to Reviewers for pointing out this problem. As the Reviewer’ comments said that the argument used to lead to the thesis is not very reasonable. We have re-written the relevant contents.

3 The entire literature related to ACP/chemical ecology are based on adult ACP behavior challenged by chemicals from plants or from co-specific insects and their VOCs. Some manuscripts explain the role of ACP adult antenna receptors challenged

Powered by Editorial Manager® and ProduXion Manager® from Aries Systems Corporation

by plant and/or chemical volatiles. It is not possible to understand the relationship of immature ACP stages with chemicals, since nymphs do not perform choices in response to chemicals, since nymphs do not perform movements. Thus, I still not convicted about the choice for nymphs only. However, the comparison of nymph antenna structures/sensillas with adults could be helpful for other studies and if done, could be more easy to justify the study.
Response: Thank you very much for your comments. We have performed the studies of adult D. citri with SEM and TEM, but we do not write in our MS. It may have many repeated contents with the article of Onagbola et al. (2008), if we reported the structures of antennae and antennal sensilla of adult ACP. The ACP has five nymphal stages, early stages are docile and move only when disturbed or overcrowded (Tsai & Liu, 2000), whereas older nymphs and adults are more mobile (Hall et al., 2013).

(Onagbola EO, Meyer WL, Boina DR, Stelinski LL. Morphological characterization of the antennal sensilla of the Asian citrus psyllid, Diaphorina citri Kuwayama (Hemiptera: Psyllidae)
(Tsai JH, Liu YH. 2000. Biology of Diaphorina citri (Hemiptera: Psyllidae) on four host plants. Journal of Economic Entomology, 93: 1721-1725.)
(Hall DG, Richardson ML, Ammar ED, Halbert SE. 2013. Asian citrus psyllid, Diaphorina citri, vector of citrus huanglongbing disease. Entomologia Experimentalis et Applicata, 146(12): 207-223.)

4 However, the authors cited 2 articles that assessed the antennal structures of adults. However, due to the lack of information (see your discussion topic), the authors are unable to explain the absence of these structures during the investigation of these articles. Maybe there is the absence of these structures in these articles, or the cited authors did not see the mentioned structures. In order to solve this doubt, I encourage the authors to add morphological aspects of adult antenna, since the literature was not helpful to support the findings with nymphs and comparison with adults.
Response: Thank you very much for your suggestions. We have performed the studies of adult D. citri with SEM and TEM, and add part of results as the supplementary data (see S1 File and S1 Table).

5 In general, the introduction is not justified neither well presented. I suggest that this topic could be re-written and that in M&M and Results could be added adult antennal structures.
Response: Thank you very much, and we have re-written this part according to your suggestion. For the adult antennal structures, Onagbola et al. (2008) had reported the research by SEM and TEM, so we think it does not need to write the similar results, just as the supplementary data in S1 File and S1 Table.

(Onagbola EO, Meyer WL, Boina DR, Stelinski LL. Morphological characterization of the antennal sensilla of the Asian citrus psyllid, Diaphorina citri Kuwayama (Hemiptera: Psyllidae)

6 There is a lack of substantial information that allow the replication of the protocols. There is no enough information related to the insect (rearing protocol, age, description of what the authors considered as nymph 1, nymph 2, nymph 3, nymph 4 and nymph 5 (see pdf manuscript with my notes).
Response: We are very sorry for our negligence of lacking the important information. We have added the relevant contents in lines 90-92. The classification characteristics of different stages of ACP nymphs are according to Ruan et al. (2012).
(Ruan CQ, Chen JL, Liu B, Duan YP, Xia YL. 2012. Morphology and behavior of Asian citrus psyllid, Diaphorina citri Kuwayama. Chinese Agricultural Science Bulletin, 28(31): 186-190).

7 Number of replicates.
Response: Done, thank you very much, and we have added the relevant content in lines 90-92.

8 More details about statistical analyses (see pdf manuscript with my notes).
Response: Done, thank you very much for your comments.

9 The beginning of discussion presents text that is related to results (see pdf

Powered by Editorial Manager® and ProduXion Manager® from Aries Systems Corporation

	manuscript with my notes). The authors are not convicted if antennal structures present in nymphs were degenerated or Onagbola et al did not see this structure in adults (but could be present). This fact did not allow the authors to be convincing during the discussion of the topic. A solution could be the assessment of adult antennal morphological aspects in order to be clear and consistent/convicted to explain the 'possible degeneration' of these structures in ACP adults.<br>Response: For the adult and nymphal ACP antennal structures, we have performed at least five times with SEM, three times with TEM. The results were the same, and we added some main adult results as the supplementary data in S1 File and S1 Table.<br><br>10 Figures should be self-explained (comments in pdf manuscript)<br>Response: Done, thank you very much.<br><br>We tried our best to improve the manuscript and made some changes in the manuscript. We appreciate for Editors/Reviewers' warm work earnestly, and hope that the correction will meet with approval.<br>Once again, thank you very much for your comments and suggestions.
Additional Information:	
Question	Response
**Financial Disclosure**<br><br>Enter a financial disclosure statement that describes the sources of funding for the work included in this submission. Review the submission guidelines for detailed requirements. View published research articles from *PLOS ONE* for specific examples.<br><br>This statement is required for submission and **will appear in the published article** if the submission is accepted. Please make sure it is accurate.<br><br>**Unfunded studies**<br>Enter: *The author(s) received no specific funding for this work.*<br><br>**Funded studies**<br>Enter a statement with the following details:<br>• Initials of the authors who received each award<br>• Grant numbers awarded to each author<br>• The full name of each funder<br>• URL of each funder website<br>• Did the sponsors or funders play any role in the study design, data collection and analysis, decision to publish, or preparation of the manuscript?<br>• **NO** - Include this sentence at the end of your statement: *The funders had no role in study design, data collection and analysis, decision to publish, or preparation of the manuscript.*<br>• **YES** - Specify the role(s) played.	This research was supported by the National Natural Science Foundation of China (31660517) and the Natural Science Foundation of Jiangxi Province, China (20161BAB214172).

* typeset	
**Competing Interests**<br><br>Use the instructions below to enter a competing interest statement for this submission. On behalf of all authors, disclose any competing interests that could be perceived to bias this work—acknowledging all financial support and any other relevant financial or non-financial competing interests.<br><br>This statement **will appear in the published article** if the submission is accepted. Please make sure it is accurate. View published research articles from *PLOS ONE* for specific examples.<br><br>**NO authors have competing interests**<br><br>Enter: *The authors have declared that no competing interests exist.*<br><br>**Authors with competing interests**<br><br>Enter competing interest details beginning with this statement:<br><br>*I have read the journal's policy and the authors of this manuscript have the following competing interests: [insert competing interests here]*<br><br>* typeset	The authors have declared that no competing interests exist.
**Ethics Statement**<br><br>Enter an ethics statement for this submission. This statement is required if the study involved:<br><br>• Human participants<br>• Human specimens or tissue<br>• Vertebrate animals or cephalopods<br>• Vertebrate embryos or tissues<br>• Field research<br><br>Write "N/A" if the submission does not require an ethics statement.	The research meets all applicable standards for the ethics of experimentation and research integrity.

General guidance is provided below. Consult the submission guidelines for detailed instructions. **Make sure that all information entered here is included in the Methods section of the manuscript.**<br><br>**Format for specific study types**<br><br>**Human Subject Research (involving human participants and/or tissue)**<br>• Give the name of the institutional review board or ethics committee that approved the study<br>• Include the approval number and/or a statement indicating approval of this research<br>• Indicate the form of consent obtained (written/oral) or the reason that consent was not obtained (e.g. the data were analyzed anonymously)<br><br>**Animal Research (involving vertebrate animals, embryos or tissues)**<br>• Provide the name of the Institutional Animal Care and Use Committee (IACUC) or other relevant ethics board that reviewed the study protocol, and indicate whether they approved this research or granted a formal waiver of ethical approval<br>• Include an approval number if one was obtained<br>• If the study involved *non-human primates*, add *additional details* about animal welfare and steps taken to ameliorate suffering<br>• If anesthesia, euthanasia, or any kind of animal sacrifice is part of the study, include briefly which substances and/or methods were applied<br><br>**Field Research**<br><br>Include the following details if this study involves the collection of plant, animal, or other materials from a natural setting:<br>• Field permit number<br>• Name of the institution or relevant body that granted permission	
**Data Availability**<br><br>Authors are required to make all data	Yes - all data are fully available without restriction

Powered by Editorial Manager® and ProduXion Manager® from Aries Systems Corporation

underlying the findings described fully available, without restriction, and from the time of publication. PLOS allows rare exceptions to address legal and ethical concerns. See the PLOS Data Policy and FAQ for detailed information.<br><br>A Data Availability Statement describing where the data can be found is required at submission. Your answers to this question constitute the Data Availability Statement and **will be published in the article**, if accepted.<br><br>**Important:** Stating 'data available on request from the author' is not sufficient. If your data are only available upon request, select 'No' for the first question and explain your exceptional situation in the text box.<br><br>Do the authors confirm that all data underlying the findings described in their manuscript are fully available without restriction?	
**Describe where the data may be found in full sentences. If you are copying our sample text, replace any instances of XXX with the appropriate details.**<br><br>• If the data are **held or will be held in a public repository**, include URLs, accession numbers or DOIs. If this information will only be available after acceptance, indicate this by ticking the box below. For example: *All XXX files are available from the XXX database (accession number(s) XXX, XXX.*).<br>• If the data are all contained **within the manuscript and/or Supporting Information files**, enter the following: *All relevant data are within the manuscript and its Supporting Information files.*<br>• If neither of these applies but you are able to provide **details of access elsewhere**, with or without limitations, please do so. For example:<br><br>*Data cannot be shared publicly because of [XXX]. Data are available from the*	All relevant data are within the manuscript and its Supporting Information files.

XXX Institutional Data Access / Ethics Committee (contact via XXX) for researchers who meet the criteria for access to confidential data.<br><br>*The data underlying the results presented in the study are available from (include the name of the third party and contact information or URL).*<br>• This text is appropriate if the data are owned by a third party and authors do not have permission to share the data.<br><br>* typeset	
Additional data availability information:	

Manuscript Click here to access/download;Manuscript;Manuscript.docx

Morphological characterization of ~~nymphal~~ antennae and antennal sensilla of *Diaphorina citri* Kuwayama (Hemiptera: Liviidae) nymphs

Lixia Zheng[1,2]*, Wensheng Chen[1]

1 College of Horticulture and Food Sciences, Foshan University, Foshan, China, **2** College of Agronomy, Jiangxi Agricultural University, Nanchang, China.

*lxzheng@fosu.edu.cn; lxzheng@jxau.edu.cn (LZ)

Abstract

Diaphorina citri Kuwayama is the most economically important citrus pest which is the primary vector of *Candidatus* Liberibacter spp. causing citrus greening (huanglongbing, HLB) disease. To better understand the developmental and structural changes of antennae and antennal sensilla in *D. citri* nymphs, we investigated the antennal morphology, structure and sensilla distribution ~~in~~ for the five nymphal stages of *D. citri* using scanning electron microscopy. The antennae of the five ~~different~~ nymphal stages were filiform in shape, which consisted of two segments in the first-, second- and third-instar nymphs; three segments in the fourth- and fifth-instar nymphs. The length of their antennae was significantly increased with the increase of the instars, as well as the total number of antennal sensilla. Eleven morphological sensilla types were recorded altogether. They were the long terminal hair (TH1), short terminal hair (TH2), sensilla trichoidea (ST), cavity sensillum 1 (CvS1), cavity sensillum 2 (CvS2), sensilla basiconica 1-4 (SB1-4), sensilla campaniform (SCA) and partitioned sensory organ (PSO). Also, the distribution of antennal sensilla in each nymphal stage of *D. citri* was asymmetrical. The SB only occurred on the antennae of the third-, fourth- and fifth-instar nymphs. Only one CvS2 was found in the third- and fifth-instar nymphs, and one SCA in the fourth- and fifth-instar nymphs, respectively. The

possible roles of the nymphal antennal sensilla in *D. citri* were discussed. The results could contribute to a better understanding of the development of the sensory system, and facilitate future studies on the antennal functions in *D. citri* nymphs.

Introduction

The Asian citrus psyllid (ACP), *Diaphorina citri* Kuwayama (Hemiptera: Liviidae), is the most economically important citrus pest because it is the primary vector of *Candidatus* Liberibacter spp. causing citrus greening (huanglongbing, HLB) disease, also the most serious citrus disease worldwide [1,2]. *Diaphorina citri* apparently originated in southern Asia, but now has spread to other world citrus-producing areas [2]. More than 50 species of the family Rutaceae have been recorded as hosts of *D. citri* [3]. Nymphs and adults suck phloem sap from the foliage causing leaf distortion, curling and deposition of honeydew on leaves that result in mold growth [3,4]. However, ~~the~~ most serious threat to citrus worldwide comes from its ability to transmit the ~~bacterium~~ bacteria, *C.* Liberibacter spp., associated with HLB, which causes yellowing of shoots, stem dieback, sour fruit, crop losses and eventually tree death [5,6].

For most insects, the antennae are peripheral sensory structures involved in habitat searching, host location, host discrimination, mating and oviposition [7-9]. They carry different kinds of sensilla which can be classified as chemosensory, mechanosensory and thermo-hygroreceptive sensilla based on their morphological and functional characteristics [7,10]. Studies on the interaction between ACP and host plant volatiles indicated the critical role of its olfactory system in finding host plants [11,12], mating [12-14], and oviposition [13,15]. Coutnho-Abreu et al. have recognized the ACP antennal neurons that responded strongly to odorants found in the host citrus plants using single-unit electrophysiology [16]. Recently, Zanadi et al. have identified the putative ACP sex pheromone [17], in addition to other related compounds that also generated electroantennographic (EAG) responses [17-19]. Thus, to analyze the antennal morphology, structure and sensilla distribution in *D. citri* is

important to explore their life cycle, olfactory behavior and host identification mechanisms.

Previous studies have described the external morphology and ultrastructure of antennal sensilla in adult *D. citri* [20], antennae sensory arrays in adult *D. citri* [21], antennal transcriptome in adult *D. citri* [22]. However, there is very little information about the morphology of nymphal antennae and antennal sensilla of psyllids. In this study, we investigated the antennal morphology, structure and sensilla distribution in five different nymphal stages of *D. citri* using scanning electron microscopy. The results could contribute to a better understanding of the development of the sensory system, and facilitate future studies on the antennal functions in *D. citri* nymphs.

Materials and methods

Insects

Specimens used in the study were five different nymphal stages of *D. citri*. These insects originated from a colony maintained at Jiangxi Agriculture University at Nanchang, China (N28°45'36", E115°49'43") that was initiated from insects collected in South China Agriculture University at Guangzhou, China (N23°10'7", E113°21'27") in June 2016. The ACP were fed on *Murraya exotica* seedlings at 27 ± 1 ºC, 70 ± 5% and 14:10 L:D photoperiod.

Scanning electron microscopy (SEM)

The whole bodies of five ~~different~~ nymphal stages of *D. citri* were fixed in 2.5% glutaraldehyde at 4 ºC for 24 h and post-fixed in 1% osmium tetraoxide for 2.5 h. The treatment process for the specimens including rinsing, dehydrating and drying was according to Yang et al. [23]. Finally, the specimens were anchored on a holder using double-sided adhesive tape in different orientations, sputter-coated with gold, examined and photographed either at 10 kV using an SEM (XL30, FEI, Holland and Nova Nano 430, FEI, Holland) or 25 kV by an SEM (JSM-6360LV, Japan) .

Statistical analysis

Classification of sensilla types in this work was based on the morphological characteristics of similar structures described previously [24-32].

Statistical tests were performed with SPSS 17.0. Sensilla on the dorsal, ventral and two lateral surfaces of five different nymphal stages of *D. citri* antennae were counted and measured. The Means (n=10) were analyzed by general linear model (GLM) procedure and Tukey's mean separation test. Mann-Whitney U test was used to compare the length and width of various stages between the long terminal hair (TH1) and short terminal hair (TH2).

Results

General morphology of antennae

observed when individuals were fixed in a ventral position and looking the region located

The antennae of *D. citri* nymphs were ~~found on the ventral side of the body~~ between the compound eyes. The morphology of five ~~different~~ the nymphal stages of *D. citri* antennae is illustrated in Fig 1. There were two terminal hairs at the tip of the antennae of each nymphal stage. Significant differences were found in the length of the antennae of each nymphal stage of *D. citri* (Table 1). The length of the five different nymphal antennae was significantly increased with the increase of the nymphal instars.

First-instar nymph

The antennae of the first-instar nymphs were composed of two segments (Fig 1A). (Table 1) They were the shortest, measuring 61.85 ± 1.83 μm in length. There was no sensillum found on the first segment of the antennae (Se1). Only six antennal sensilla belonging to five morphologically different types were observed on the antennae of the first-instar nymphs. They were the TH1, TH2, sensilla trichoidea (ST), partitioned sensory organ (PSO) and cavity sensillum 1 (CvS1).

Second-instar nymph

The antennae of th[illegible]cond-instar nymphs were also composed of two segments (Fig 1B). They were 35.96 μm longer than the first-instar nymphal antennae (Table 1). The sensilla occurred on the first-instar nymphal antennae were also found in the second-instar nymphs. Moreover, there was ST found on the Se1 of the second-instar nymphs.

Third-instar nymph

The antennae of the third-instar nymphs were similar to those of the first- and second-instar nymphs, which were also composed of two segments (Fig 1C). The antennae of the third-instar nymphs were about 1.5 times longer than those of the second-instar nymphs (Table 1). Ten morphologically different types of sensilla were detected on the third-instar nymphal antennae. Th[illegible] were TH1, TH2, ST, PSO, four types of sensilla basiconica (SB1-4), CvS1 and CvS2.

Fourth-instar nymph

The antennae of the fourth-instar nymphs were composed of three parts: two basal segments and the flagellum with poorly defined subsegments (Fig 1D). The types of antennal sensilla in the fourth-instar nymphs were one less, but the total number was one more than those in the third-instar nymphs (Table 2). The SB4 and CvS2 were missed, however, the sensilla campaniform (SCA) was found in the fourth-instar nymphs.

Fifth-instar nymph

The antennae of the fifth-instar nymphs were simila[illegible] those of the fourth-instar nymphs, whi[illegible]were also composed of three parts (Fig 1F). They were the longest antennae in the five different nymphal stages of *D. citri* (Table 1). The types and the total number of antennal sensilla in the fifth-instar nymphs were the most (Table 2).

Eleven morphologically different types of sensilla were detected on the fifth-instar nymphal antennae. They were TH1, TH2, ST, PSO, CvS1, CvS2, SB1-4 and SCA.

Morphology and structure of sensilla

Table 2 shows the antennal sensilla distributions in five different nymphal stages of *D. citri*. We observed the asymmetrical distribution of antennal sensilla in different nymphal stages. ST, TH1, TH2, PSO and CvS1 were found on the antennae of each nymphal stage of *D. citri*; CvS2 and SB4 only occurred on the antennae of the third- and fifth-instar nymphs, and one SCA was found in the fourth- and fifth-instar nymphs. In addition, SB1-3 was observed on the antennae of the third- fourth- and fifth-instar nymphs.

Terminal hairs

There were two bristle-like sensilla apically on the terminal antennae of the five different nymphal stages (Fig 1 ~~and 2A~~). The TH1 was strong and straight with longitudinal grooves (Fig 2A). They were inserted into a big cuticular socket. The TH2 had a similar morphology to the TH1, but much shorter and stronger (Fig 2A and 2B, Table 3). The length of TH1 was significantly longer than that of the TH2 in each development stage. The lengths of TH1 and TH2 in the second-instar nymphs were the shortest, measuring 13.38 ± 0.66 μm and 9.88 ± 0.33 μm, respectively. In addition, the width of TH2 in the fifth-instar nymphs was significantly bigger than that in other nymphal stages and that of the TH1 (Table 3).

), with an enfasis in thrid instar nymph (Fig 2A).

Sensilla trichoidea

Sensilla trichoidea (ST) was found on the antennae of each nymphal stage of *D. citri*. They were straight hairs with a smooth surface and a big conical socket (~~Fig 3~~). Only one ST occurred on the Se1 of the second-, third- and fourth-instar nymphs. There was also only one ST positioned just below the base of the TH1 in each development

Table 2 and Fig 3).

stage (Fig 3A).

Partitioned sensory organ

Partitioned sensory organ (PSO) was located on the ventral side of the ~~last~~ segment of the antennae of each nymphal stage of *D. citri*. The PSO formed with an open pit and a sheet structure from which a cavity sensillum protruded (Table 2 and Fig 4) (~~Fig 4~~). The inner side of the PSO was enclosed by the cuticular fringe (Fig 4B). One large PSO was found in the first-instar nymphs, two in the second- and third-instar nymphs, three in the fourth-instar nymphs and four in the fifth-instar nymphs, respectively.

Cavity sensillum

Only one cavity sensillum 1 (CvS1) was found below the base of the TH2 in each nymphal stage. They were an oval sensory cavity formed by invaginations of the antennal cuticle and contained two sensilla (Fig 5A and S1 Fig). The cuticular parts of the two sensilla sitting on the bottom of the cavity were formed as short pegs, terminating just below the antennal surface. The tips of two short pegs were flat, and one of them was flower-shaped.

Cavity sensillum 2 (CvS2) was only found on the ventral side of the base of the third segment of the fifth-instar nymphal antennae. Similar to the CvS1, they were a round sensory cavity containing only one sensilla peg with a flower-shaped tip (Fig 5B and 5C). The cavities were rounded, and the flower-shaped tip was not flat.

Sensilla basiconica

Only one sensilla basiconica 1 (SB1) was observed on the base of the Se1 of the third-, fourth- and fifth-instar nymphs. (Table 2) The SB1 was fitted into a tight socket with a sharp tip and was almost vertical to the longitudinal axis of the antennae (Fig 6A). It had a smooth surface, measuring 4.58 μm in length.

Sensilla basiconica 2 (SB2) was distributed on the dorsal side of the third-,

fourth- and fifth-instar nymphal antennae Table 2). The SB2 was also fitted into a big socket elevated above the cuticle, measuring 6.35 μm in length and 1.73 μm in basal diameter. They displayed a longitudinally grooved surface with a sharp tip (Fig 6B).

Sensilla basiconica 3 (SB3), similar to the SB2, was also found on the dorsal side of the third-, fourth- and fifth-instar nymphal antennae (Table 2). They were robust on the antennae, grew in a big basal socket, and measured 31.07 μm in length and 3.31 μm in basal diameter. The SB3 had multiple-pitted surfaces formed by clutter gullies, like the amygdala (Fig 6C). The number of SB3 was much more than that of the SB2 (Table 2).

Sensilla basiconica 4 (SB4) was sparsely distributed on the dorsal side of the last segment of the third- and fifth-instar nymphal antennae. Two SB4 were found in the third-instar nymphs, and one in the fifth-instar nymphs (Table 2). The SB4 had a wavy line surface, like noodles (Fig 6D). They were also robust and grew in a big basal socket, measuring 12.7 μm in length and 3.86 μm in basal diameter.

Sensilla campaniform

A single sensilla campaniform (SCA) was found on the dorsal side of the second segment of the antennae of the fourth- and fifth-instar nymphs (Table 2 and Fig 7) (~~Fig 7~~). The SCA had a dome-shaped sensory structure. Their ambient cuticle was protuberant but sunken in the middle (Fig 7A). In addition, there was a concave spot at the sunken structure in SCA (Fig 7B).

Discussion

The morphology and ultrastructure of the antennae and antennal sensilla in different nymphal stages of *D. citri* were studied. The antennae of *D. citri* were composed of two segments in the first-, second- and third-instar nymphs, and two basal segments and the flagellum with poorly defined subsegments in the fourth- and fifth-instar nymphs (Fig 1). The length of the antennae of each development stage was significantly increased with the increase of the instars (Table 1), as well as the types

Highlighted in yellow and red: Results replicated

and total number of the antennal sensilla (Table 2). At any stage of the life cycle of *D. citri*, the total number of antennal sensilla was small (Table 2 and S1 Table); moreover, their structure was rather simple. Eleven morphologically distinct types of sensilla were found on the antennae of five different nymphal stages of *D. citri* in our study. We do not find the SB1-4, CvS1 and CvS2 in adult *D. citri* (S1 File and S1 Table), as well as Onagbola et al. [20], maybe they were degenerated and missed in adults. The PSO of the nymphal instars had a 'raised edges and internal shutter-like' appearance which might be replaced by the antennal rhinaria in adult *D. citri* (S1 File).

The TH were analogous with those found in *Psylla pyricola* Förster (Hemiptera: Psyllidae) [27], *Trialeurodes vaporariorum* (Westwood) (Hemiptera: Aleyrodidae), *Aleyrodes proletella* (Linnaeus) (Hemiptera: Aleyrodidae), *Bemisia tabaci* (Gennadius) (Hemiptera: Aleyrodidae) [30,33], *Trioza apicalis* (Hemiptera: Triozidae) [31], *Dialeurodes citri* (Ashmead) (Hemiptera: Aleyrodidae) [34], *Aleurodicus dispersus* Russell (Hemiptera: Aleyrodidae) [32,35]. As is the case with other types of sensilla, the terminal hairs have been differently named by various authors. For example, Singleton-Smith et al. [27] and Mellor and Anderson [30] characterized them as “chaetica sensilla”, which can be suggested to serve as a proprioreceptor perceiving antennal movement and position or have mechano-sensory functions [20,36], due to their location and presence to membranous connections between the sensillar shaft and the antennal cuticular surfaces [37]. In addition, considering the TH1 and TH2 in adults (Fig 2 in S1 File) that revealed wall pores suggesting a plausible role in perception of olfactory stimuli.

Sensilla trichoidea have been reported to perform either or both mechano- and chemo-sensory function [7]. The ST with no cellular material and pore canal structures (S2 Fig) resembled the mechanosensory hair which positioned just below the base of the TH1 in carrot psyllid [31]. In addition, the ST present in analogous locations with the AST-2 described by Onagbola et al. [20] may also suggest to have mechanosensory functions [20,31].

The PSO was first reported in *P. pyricola* and suggested chemoreceptors [27]. Their morphology, location, and number were similar in five different nymphal instars

between *P. pyricola* and *D. citri*. Previous studies have reported that the descriptions of the plate organs (sensilla placodea) in aphids conformed closest to the large sensory organ (PSO) [25,38-40]. Therefore, if these were indeed plate organs that were placed in the category of chemoreceptor by Slifer [40], they could be involved in olfactory function.

The CvSs were the compound sensilla which consisted of a sensory cavity and the sensilla pegs, but they were only meant the sensory cavity named by Kristoffersen et al. [31] and Onagbola et al. [20] in psyllids. The difference between CvS1 and CvS2 was the number of pegs in the cavity. All of their pegs were well hidden beneath the antennal surface, and their position was far from optimal for receiving chemical stimuli. Kristoffersen et al. [31] have reported that these pegs are likely to be a necessary adaptation to prevent desiccation. Moreover, several studies have demonstrated that sensilla that are recessed from the antennal surface and located within cavities may be involved in perception of the CO_2, humidity, and temperature [41-43]. Judging by morphological features, the CvS1 and CvS2 could be responsible for the perception of any type of stimuli as described above.

The SBs only occurred on the antennae of third-, fourth- and fifth-instar nymphs. Similarly, they were not reported in other psyllids, no matter nymphs or adults [20,27,31]. In our study, the SBs were inserted into a big inflexible socket and exhibited different morphological surfaces (Fig 6). Judging by the morphological surface features, four subtypes of this sensillum can be identified. According to Schneider [25], sensilla basiconica are the most common and important chemoreceptors found on the antennae of insects. Moreover, Altner and Prillinger [44] and Zacharuk [45] suggested that it would be impossible for sensilla with an inflexible socket to act as a mechanoreceptor.

The SCA has been found in many insects, including whiteflies [36] and psyllids [27]. On the antennae of the fourth- and fifth-instar nymphs, only one SCA was presented on the second segment of the antennae. The SCA was seemed to occur in aging nymphs (the fourth-instar nymph of *A. dispersus* and the fifth-instar nymph of *P. pyricola*) [27,32] or adults (*Tetrastichus howardi* and *A. dispersus*) [35,46]. Previous

studies have reported that SCA with no pores in their cuticular structure play the role of mechanoreceptors [29,37,47-51], whereas the SCA with pores was involved in gustatory function were highly susceptible to humidity [52]. Here they probably acted as mechanoreceptors, because they were few and located near the segmental joints [7,26,53,54].

In our study, the types and total number of the antennal sensilla obviously increased in the third-, fourth- and fifth-instar nymphs. According to Hung et al. [55], *C.* Liberibacter asiaticus (Las) persists in the ACP vector but is not transovarially transmitted by the vector. The pathogen can only be transmitted by the third-, fourth- and fifth-instar nymphs and adults which play a major role in the spread of the pathogen because of their dispersal capabilities [2,55-57], but the nymphs were found to be more efficient [4]. Further studies are needed to determine whether the antennal sensilla in the third-, fourth- and fifth-instar nymphs (especially the SBs) are related to the feeding behavior on the citrus with Las by these nymphs. In conclusion, we have provided an extensive description of the antennae and antennal sensilla in different nymphal stages of *D. citri* using SEM. This information can be a great help for revealing the developmental course of psyllid's antennae and antennal sensilla and allow us to better understand the host location mechanism in *D. citri*.

Acknowledge

The authors are grateful to Wei-Chun Li from Jiangxi Agricultural University for English revision and critical reading of the manuscript. We would like to thank Ms Xiao-Yin Hu from the Centre Laboratory of the South China Botanical Garden for her valuable assistance with the treatment for the specimens in SEM. Special thanks to Wei-Jian Wu from South China Agricultural University for statistical analysis.

Reference

1. Bové JM. Huanglongbing: a destructive, newly-emerging, century-old disease of citrus. Journal of Plant Pathology. 2006; 88: 7-37.

2. Grafton-Caldwell EE, Stelinski LL, Stansly PA. Biology and management of Asian citrus psyllid, vector of the Huanglongbing pathogens. Annual Review of Entomology. 2013; 58: 413-432.

3. Halbert SE, Manjunath KL. Asian citrus psyllids (Sternorrhyncha: Psyllidae) and greening disease of citrus: a literature review and assessment of risk in Florida. Fla Entomol. 2004; 87: 330-353.

4. Ammar ED, Alessandro R, Shatters JrRG, Hall DG. Behavioral, ultrastructural and chemical studies on the honeydew and waxy secretions by nymphs and adults of Asian citrus psyllid *Diaphorina citri* (Hemiptera: Psyllidae). PloS One. 2013; 8(6): e64938.

5. McClean PD, Schwarz RE. Greening or blotchy mottle disease of citrus. Phytophylactica. 1970; 2: 177-194.

6. Dagulo L, Danyluk MD, Spann TM, Valim MF, Goodrich-Schneider R, Sims C, et al. Chemical characterization of orange juice from trees infected with citrus greening (Huanglongbing). Journal of Food Science. 2010; 75(2): C199-C207.

7. Zacharuk RY. Antennae and sensilla. In: Kerkut GA, Gilbert LJ, editors. Comprehensive Insect Physiology, Biochemistry, and Pharmacology, Sensory. Oxford: Pergamon; 1985. p.1-69.

8. Bland RG. Antennal sensilla of acrididae (Orthoptera) in relation to subfamily and food preference. Annals of the Entomological Society of America. 1989; 82: 368-384.

9. Blaney WM, Simmonds MSJ. The chemoreceptors. In: Chapman RE, Joern A, editors. Biology of Grasshoppers. New York: Wiley; 1989. p.1-37.

10. Gullan PJ, Cranston PS. The Insects: an Outline of Entomology, third ed. London: Blackwell Publishing Ltd; 2004. p. 86-105.

11. Patt JM, Sétamou M. Responses of the Asian citrus psyllid to volatiles emitted by flushing shoots of its rutaceous host plants. Environmental Entomology. 2010; 39: 618-624.

12. Moghbeli Gharaei A, Ziaaddini M, Jalali MA, Michaud JP. Sex-specific responses of Asian citrus psyllid to volatiles of conspecific and host-plant origin. Journal of

Applied Entomology. 2014; 138(7): 500-509.

13. Wenninger EJ, Hall DG. Daily timing of mating and age at reproductive matruity in *Diaphorina citri* (Hemiptera: Psyllidae). Florida Entomologist. 2007; 90: 715-722.

14. Wenninger EJ, Stelinski LL, Hall DG. Behavioral evidence for a female-produced sex attractant in *Diaphorina citri* (Hemiptera: Psyllidae). Entomol Exp Appl. 2008; 128: 450-459.

15. Moran VC, Buchan PR. Oviposition by the citrus psylla, *Trioza erytreae* (Homoptera: Psyllidae), in relation of leaf hardiness. Entomologia Experimental et Applicata. 1975; 18: 96-104.

16. Coutinho-Abreu IV, McInally S, Forster L, Luck R, Ray A. Odor coding in a disease-transmitting herbivorous insect, Asian citrus psyllid. Chemical Senses. 2014; 39: 539-549.

17. Zanardi OZ, Volpe HXL, Favaris AP, Silva WD, Luvizotto RAG, Magnani RF, et al. Putative sex pheromone of the Asian citrus psyllid, *Diaphorina citri*, breaks down into an attranctant. Scientific Reports. 2018; 8(1): 455.

18. George J, Robbins PS, Alessandro RT, Stelinski LL, Lapointe SL. Formic and acetic acids in degradation products of plant volatiles elicit olfactory and behavioral reponses from an insect vector. Chemical Senses. 2016; 41: 325-338.

19. Zanardi OZ, Volpe HXL, Luvizotto RAG, Magnani RF, Gonzalez F, Calvo C, et al. Laboratory and field evaluation of acetic acid-based lures for male Asian citrus psyllid, *Diaphorina citri*. Scientific Reports. 2019; 9(1): 1-10.

20. Onagbola EO, Meyer WL, Boina DR, Stelinski LL. Morphological characterization of the antennal sensilla of the Asian citrus psyllid, *Diaphorina citri* Kuwayama (Hemiptera: Psyllidae), with reference to their probable functions. Micron. 2008; 39(8): 1184-1191.

21. Arras J, Hunter W, Bextine B. Comparative analysis of antennae sensory arrays in Asian citrus psyllid, *Diaphorina citri*, and potato psyllid, *Bactericera cockerelli* (Hemiptera). Southwestern Entomologist. 2012; 37(1): 1-12.

22. Wu ZZ, Zhang H, Bin SY, Chen L, Han QX, Lin JT. Antennal and abdominal

transcriptomes reveal chemosensory genes in the Asian citrus psyllid, *Diaphorina citri*. PloS One. 2016; 11(7): e0159372.

23. Yang HY, Zheng LX, Zhang ZF, Zhang Y, Wu WJ. The structure and morphologic changes of antennae of *Cyrtorhinus lividipennis* (Hemiptera: Miridae: Orthotylinae) in different instars. PloS One. 2018; 13(11): e0207551.
24. Dethier VG. The physiology of insect senses. London: Methuen and Co. Ltd; 1963.
25. Schneider D. Insect antenna. Annual Review of Entomology.1964; 9: 103-122.
26. Moran VC, Brown RP. The antennae, host plant chemoreception and probing activity of citrus psylla, *Trioza erytreae* (Del Guercio) (Homoptera: Psyllidae). Journal of the Entomological Society of Southern Africa. 1973; 36(2): 191-202.
27. Singleton-Smith J, Chang JF, Philogène JR. Morphological differences between nymphal instars and descriptions of the antennal sensory structures of the nymphs and adults of *Psylla pyricola* Foerster (Homoptera: Psyllidae). Canadian Journal of Zoology-Revue Canadienne de Zoologie. 1978; 56(7): 1576-1584.
28. Bromley AK, Dunn JA, Anderson M. Ultrastructure of the antennal sensilla of aphids I. coeloconic and placoid sensilla. Cell and Tissue Research. 1979; 203: 427-442.
29. Zacharuk RY, Shields VD. Sensilla of immature insects. Annual Review of Entomology. 1991; 36: 331-354.
30. Mellor HE, Anderson M. Antennal sensilla of whiteflies: *Trialeurodes vaporariorum* (Westwood), the glasshouse whitefly, *Aleyrodes proletella* (Linnaeus), the cabbage whitefly, and *Bemisia tabaci* (Gennadius), the tobacco whitefly (Homoptera: Aleyrodidae). Part 1: external morphology. International Journal of Insect Morphology and Embryology. 1995; 24(2): 133-143.
31. Kristoffersen L, Hallberg E, Wallén R, Anderbrant O. Sparse sensillar array on *Trioza apicalis* (Homoptera, Triozidae) antennae——an adaptation to high stimulus levels? Arthropod Structure and Development. 2006; 35: 85-92.
32. Zheng LX, Wu WJ, Liang GW, Fu YG. Nymphal antennae and antennal sensilla in *Aleurodicus dispersus* (Hemiptera: Aleyrodidae). Bulletin of Entomological

Research. 2014; 104: 622-630.

33. Zhang XM, Wang S, Li S, Luo C, Li YX, Zhang F. Comparison of the antennal sensilla ultrastructure of two cryptic species in *Bemisia tabaci*. PLoS One. 2015; 10(3): e0121820.

34. Zheng LX, Xiao TT, Wu WJ, Cui ZX, Fu YG. Ultra-structure observation on the antenna sensilla of adult citrus whitefly *Dialeurodes citri*. Journal of Fruit Science. 2011; 28(2): 344-347.

35. Zheng LX, Wu WJ, Fu YG. Sensilla on antenna of adult spiraling whitefly *Aleurodicus dispersus*. Chinese Bulletin of Entomology. 2010; 47(3): 525-528.

36. Ochieng SA, Park KC, Zhu JW, Baker TC. Functional morphology of antennal chemoreceptors of the parasitoid *Microplitis croceipes* (Hymenoptera: Braconidae). Arthropod Structure and Development. 2000; 29: 231-240.

37. Chapman RF, Chapman RF. The insects: structure and function, Cambridge University Press; 1998.

38. Slifer EH, Sekhon SS, Lees AD. The sense organs on the antennal flagellum of aphids (Homoptera), with special reference to the plate organs. Quarterly Journal of Microscopical Science. 1964; 105: 21-30.

39. Imms AD. A general textbook of entomology, ninth ed. London: Methuen and Co. Ltd; 1970.

40. Slifer EH. The structure of arthropod chemoreceptors. Annual Review of Entomolpgy. 1970; 15: 121-142.

41. Steinbrecht RA. Arthropods: Chemo-, thermo-, and hygro-receptor. In: Bereiter-Hahn J, Matolsty AG, Richards KS, editors. Biology of the intergument, Vol. 1. Berlin: Spring-Verlag; 1984. p. 523-553.

42. Stange G, Stowe S. Carbon-dioxide sensing structures in terrestrial arthropods. Microscopy Research and Technique. 1999; 17: 416-427.

43. Onagbola EO, Fadamiro HY. Scanning electron microscopy studies of antennal sensilla of *Pteromalus cerealellae* (Hymenoptera: Pteromalidae). Micron. 2008; 39: 526-535.

44. Altner H, Prillinger L. Ultrastructure of invertebrate chemo-, thermo-, and

hygroreceptors and its functional significance. International Review of Cytology. 1980; 67: 69-139.

45. Zacharuk RY. Ultrastructure and function of insect chemosensilla. Annual Review of Entomolpgy. 1980; 25: 27-47.

46. Zheng YH, Zheng LX, Liao YL, Wu WJ. Sexual dimorphism in antennal morphology and sensilla ultrastructure of a pupal endoparasitoid *Tetrastichus howardi* (Olliff) (Hymenoptera: Eulophidae). Microscopy Research and Technique. 2016; 79: 374-384.

47. Slifer EH, Sekhon SS. Fine structure of the sense organs on the antennal flagellum of the honey bee, *Apis mellifera*. Linnaeus. Journal of Morphology. 1961; 109: 351-381.

48. Stort CA, Barelli N. Genetic study of olfactory structures in the antennae of two *Apis mellifera* subspecies. Journal of the Kansas Entomological Society. 1981; 54: 352.

49. Hashimoto Y. Unique features of sensilla on the antennae of Formicidae (Hymenoptera). Journal of Applied Entomology and Zoology. 1990; 25: 491-501.

50. Olson DM, Andow DA. Antennal sensilla of female *Trichogramma nubilale* (Ertle and Davis) (Hymenoptera: Trichogrammatidae) and comparisons with other parasitic Hymenoptera. International Journal of Insect Morphology & Embryology. 1993; 22: 507-520.

51. Kleineidam C, Romani R, Tautz J, Izidoro N. Ultrastructure and physiology of the CO_2 sensitive sensillum ampullaceum in the leaf-cutting ant *Atta sexdens*. Arthropod Structure and Development. 2000; 29: 43-55.

52. Dietz A, Humphreys WJ. Scanning electron microscopic studies of antennal receptors of the worker honey bee, including sensilla campaniformia. Annals of the Entomological Society of America. 1971; 64: 919-925.

53. McIver SB. Mechanoreception. In: Kerkut GA, Gilbert LI, editors. Comparative Insect Physiology, Biochemistry and Pharmacology, Pergamon Press, Oxford; 1985. p. 71-132.

54. Liang AP, Fletcher JM. Morphology of the antennal sensilla in four Australian spittlebug species (Hemiptera: Ceropidae) with implications for phylogeny. Australian Journal of Entomology. 2002; 41: 39-44.

55. Hung TH, Hung SC, Chen CN, Hsu MH, Su HJ. Detection by PCR of *Candidatus* Liberibacter asiaticus, the bacterium causing citrus huanglongbing in vector psyllids: application to the study of vector pathogen relationships. Plant Pathology. 2004; 53: 96-102.

56. Boina DR, Rogers ME, Wang N, Stelinski LL. Effect of pyriproxyfen, a juvenile hormone mimic, on egg hatch, nymph development, adult emergence and reproduction of the Asian citrus psyllid, *Diaphorina citri* Kuwayama. Pest Management Science. 2010; 66: 349-357.

57. Cen Y, Yang C, Holford P, Beattie GAC, Spooner-Hart RN, Liang G, et al. Feeding behaviour of the Asiatic citrus psyllid, *Diaphorina citri*, on healthy and hunaglongbing infected citrus. Entomologia Experimental et Applicata. 2012; 143: 13-22.

Supporting information

S1 Fig. Transmission electron microscopy micrograph of the transect through the cavity sensillum 1 of the first-instar *Diaphorina citri*, showing the two sensilla in the cavity (arrows).

(DOCX)

S2 Fig. Transmission electron microscopy micrograph of the sensilla trichoidea positioned below the base of the TH1 in the third-instar *Diaphorina citri* nymphs.

(DOCX)

S1 Table. Abundance and distribution of sensilla on the antennae of adult *Diaphorina citri*.

(DOCX)

S1 File. General analysis of the antennae and antennal sensilla of adult *Diaphorian citri*.

(DOCX)

the five nymphal

Table 1. Length (mean ± SE) of ~~various~~ nymphal stages of *Diaphorina citri* antennae.

Nymphal stages	*N*	Length (μm)
First-instar	10	61.85 ± 1.83e
Second-instar	10	97.81 ± 1.98d
Third-instar	10	150.83 ± 4.39c
Fourth-instar	10	188.38 ± 20.69b
Fifth-instar	10	316.11 ± 3.22a

Add a column Growth um and add analyses

Means with same letters in the same column are not significantly different (GLM, Tukey, P>0.05).

Table 2. Abundance and distribution of sensilla on the antennae ~~of various~~ for the five nymphal stages of *Diaphorina citri*.

Nymphal stages		Sensilla											Total	
		ST	TH1	TH2	PSO	CvS1	CvS2	SCA	SB1	SB2	SB3	SB4		
First-instar	Se1	—	—	—	—	—	—	—	—	—	—	—	—	6
	Se2	2	1	1	1	1	—	—	—	—	—	—	6	
Second-instar	Se1	1	—	—	—	—	—	—	—	—	—	—	1	9
	Se2	3	1	1	2	1	—	—	—	—	—	—	8	
Third-instar	Se1	1	—	—	—	—	—	—	1	—	—	—	2	17
	Se2	3	1	1	2	1	1	—	—	1	3	2	15	
Fourth-instar	Se1	1	—	—	—	—	—	1	1	—	—	—	3	18
	Se2	—	—	—	—	—	—	—	—	1	1	—	2	
	Se3	4	1	1	3	1	—	—	—	1	2	—	13	
Fifth-instar	Se1	4	—	—	—	—	—	1	1	—	—	—	6	31
	Se2	3	—	—	—	—	—	—	—	—	3	—	6	
	Se3	5	1	1	4	1	1	—	—	3	2	1	19	

Number and location of the various sensilla observed on the antennae of various nymphal stages of *D. citri*. Se1, Se2 and Se3, the first, second and third segment of the antennae; ST, sensilla trichoidea; TH1 and TH2, the long and short terminal hair at the tip of the antennae, respectively; PSO, partitioned sensory organ; CvS1 and CvS2, cavity sensillum 1 and 2, respectively; SCA, sensilla campaniform; SB1, SB2, SB3 and SB4, sensilla basiconica 1, 2, 3 and 4, respectively; '—', indicates absent.

Table 3. Length and width (mean ± SE, *n*=10) of TH1 and TH2 of various nymphal stages of *Diaphorina citri*.

Stages	Length (μm)		Width(μm)	
	TH1	TH2	TH1	TH2
First-instar	16.03±0.62ABa	13.60±0.68ABb	2.16±0.14Ca	1.99±0.14Ca
Second-instar	13.38±0.66Ba	9.88±0.33Bb	2.37±0.20Ca	2.45±0.09Ca
Third-instar	16.34±0.85ABa	10.30±0.83Bb	2.53±0.09Ca	2.73±0.19BCa
Fourth-instar	18.87±1.33ABa	13.00±0.51ABb	2.90±0.25BCa	3.65±0.30Ba
Fifth-instar	21.89±1.32Aa	15.13±1.23Ab	4.05±0.49ABa	4.81±0.26Ab

Means in columns with same uppercase letters are not significantly different (GLM, Tukey, *P*>0.05). Means in rows with same lowercase letters are not significantly different (*P*>0.05) in Mann–Whitney *U* test.

Figure
Click here to access/download;Figure;Figures.doc

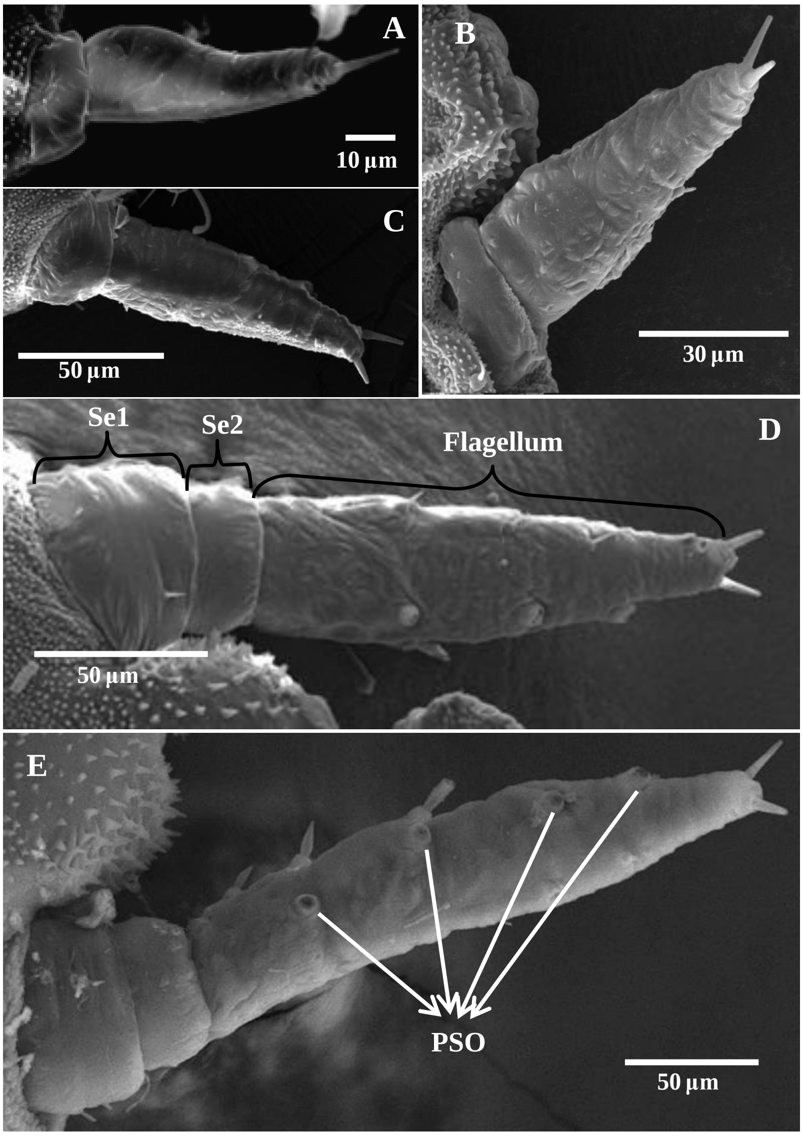

Fig 1. General morphology of antennae of immature *Diaphorina citri*. (A-C) SEM photographs in the first-, second- and third-instar of *D. citri* nymphal antennae with two segments. (D-~~H~~ E????) SEM photographs in the fourth- and fifth-instar of *D. citri* nymphal antennae with three segments. PSO, partitioned sensory organ; Se1, the first segment of the antennae; Se2, the second segment of the antennae.

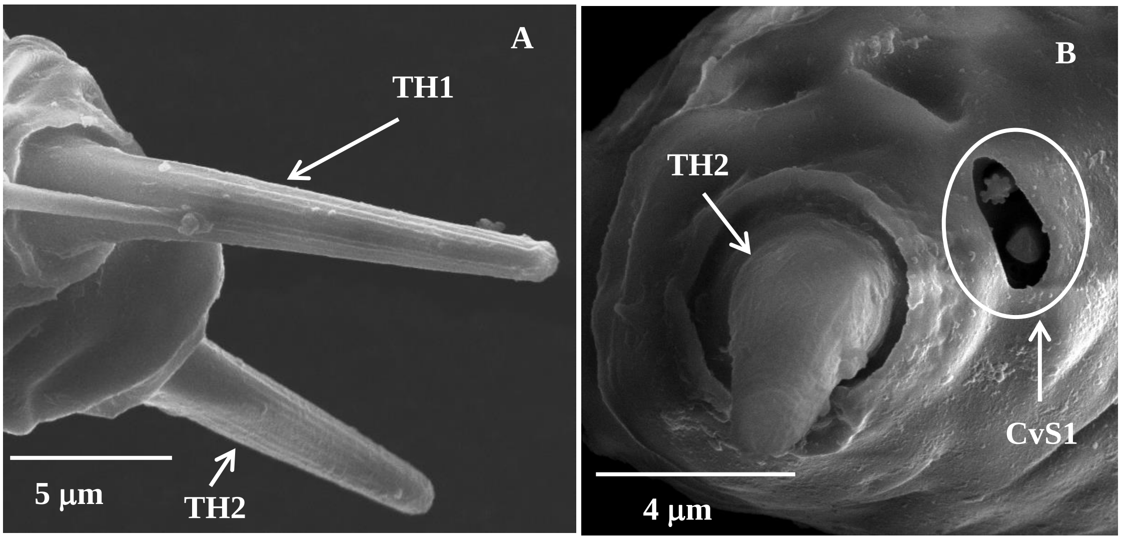

Fig 2. The antennal tip of the third-instar *Diaphorina citri* nymphs. (A) Two terminal hairs and sensilla trichoidea. (B) The short terminal hair and cavity sensillum 1. TH1, the long terminal hair; TH2, the short terminal hair; CvS1, cavity sensillum 1.

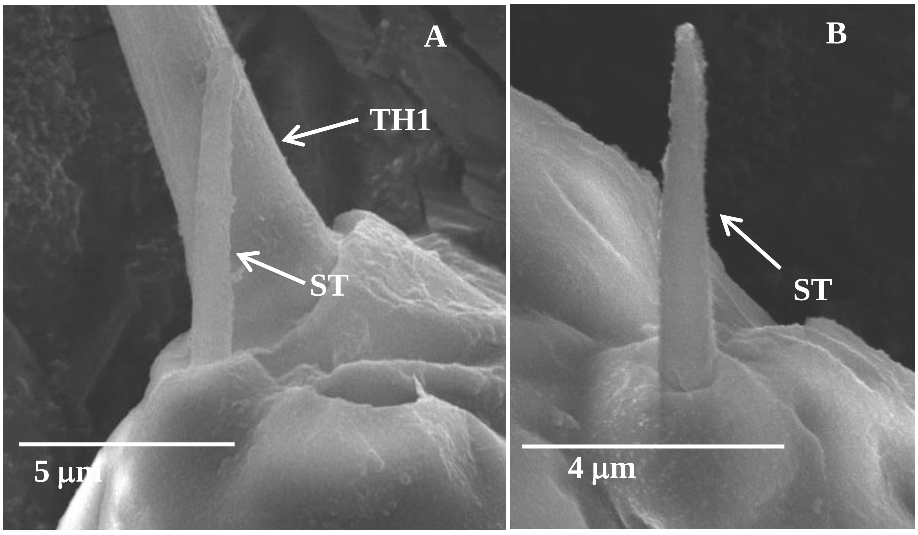

Fig 3. Sensilla trichoidea in the third-instar *Diaphorina citri* nymphs. (A) The long terminal hair (TH1) and sensilla trichoidea (ST). (B) Sensilla trichoidea.

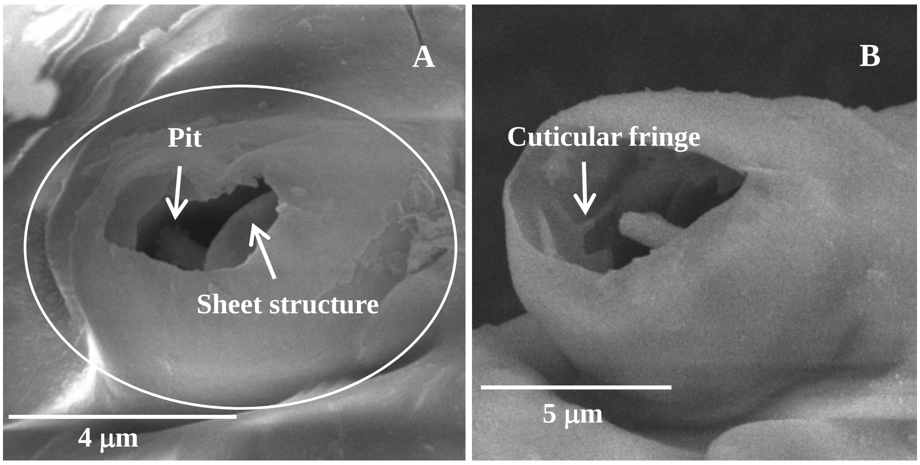

Fig 4. Partitioned sensory organ in the fifth-instar *Diaphorina citri* nymphs. (A) Partitioned sensory organ, showing the pit and sheet structure. (B) Partitioned sensory organ, showing the cuticular fringe.

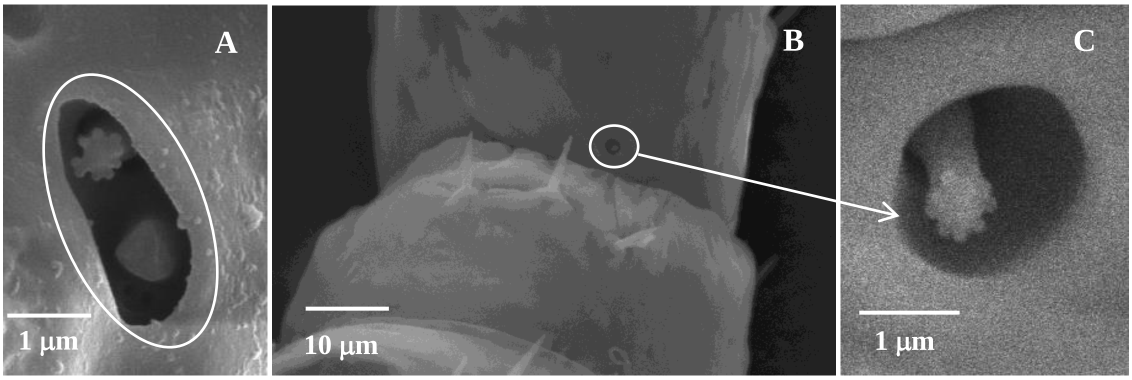

Fig 5. Cavity sensillum in the fifth-instar *Diaphorina citri* nymphs. (A) Cavity sensillum 1, containing an oval sensory cavity and two pegs. (B) Cavity sensillum 2, containing a round sensory cavity and one peg. (C) The high magnification picture of cavity sensillum 2.

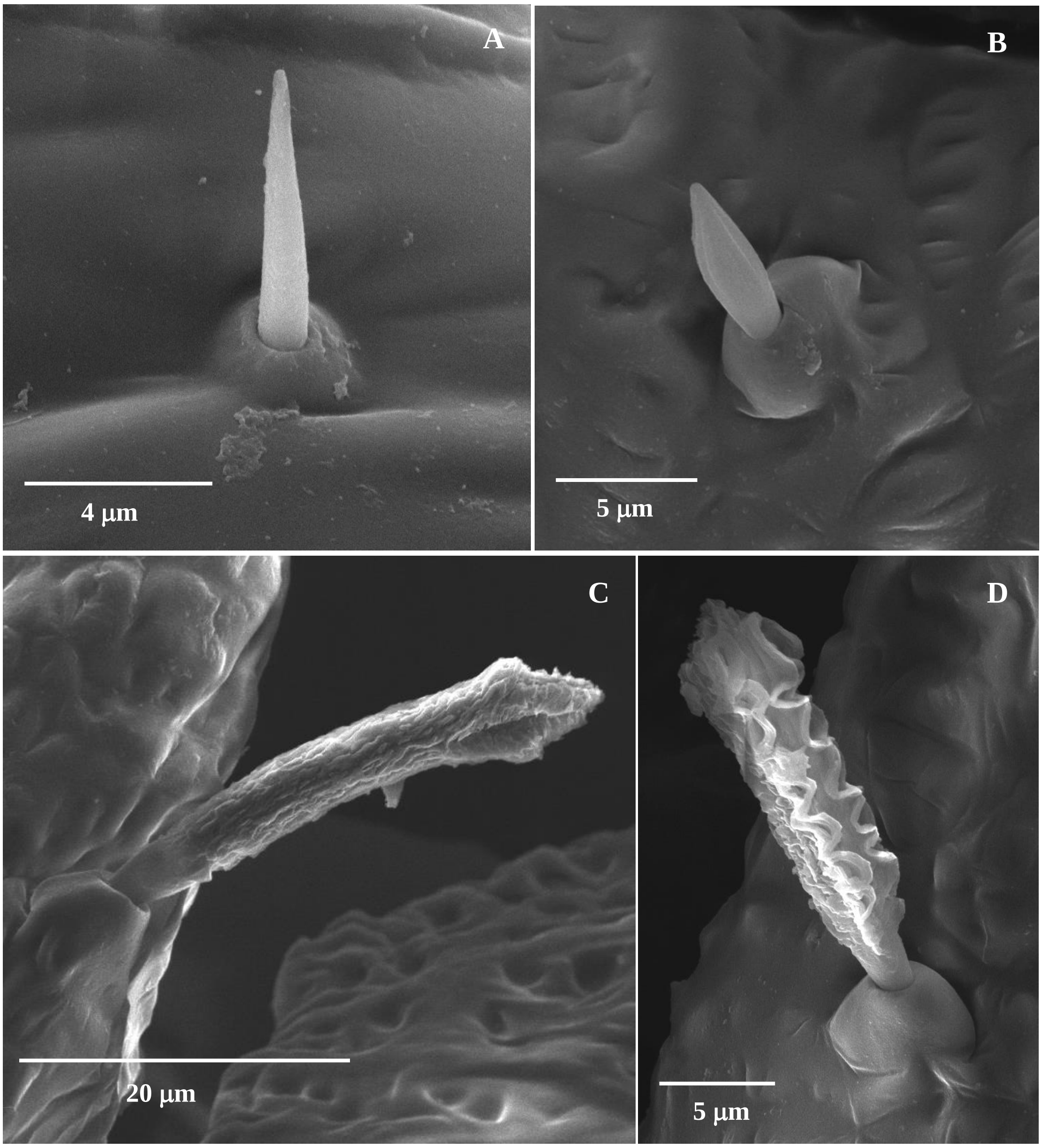

Fig 6. Sensilla basiconica in the fourth-instar *Diaphorina citri* nymphs. (A) Sensilla basiconica 1. (B) Sensilla basiconica 2. (C) Sensilla basiconica 3. (D) Sensilla basiconica 4.

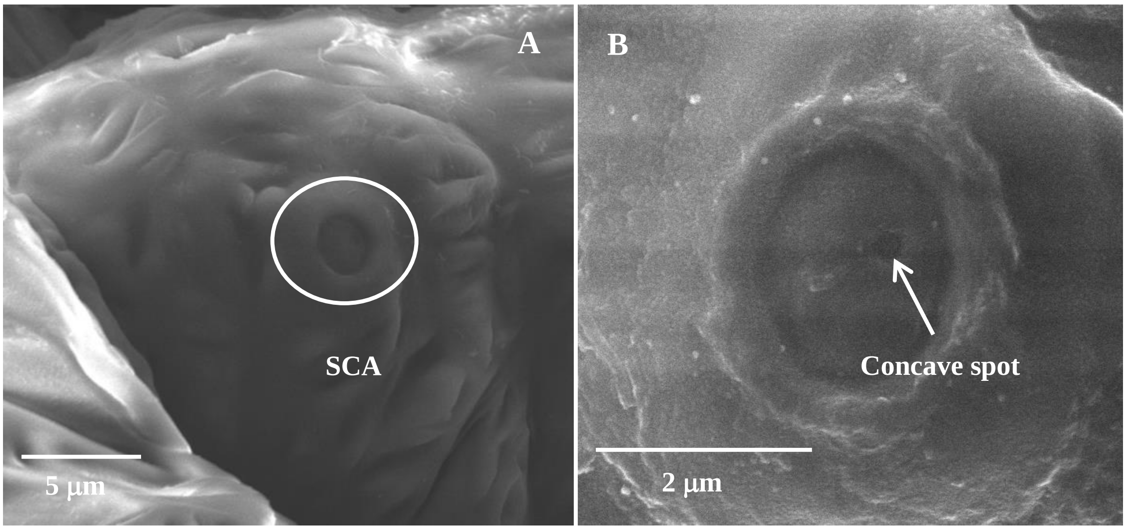

Fig 7. Sensilla campaniform (SCA) in fourth-instar *Diaphorina citri* nymphs. (A) Sensilla campaniform on the second segment of the antennae. (B) Sensilla campaniforn, showing the concave spot.

S2 Fig

Click here to access/download
Supporting Information
S2 Fig.docx

Click here to access/download
Supporting Information
S1 File.doc

S1 Table

Click here to access/download
Supporting Information
S1 Table.docx

S1 Fig

Click here to access/download
Supporting Information
S1 Fig.doc

Morphological characterization of nymphal antennae and antennal sensilla of *Diaphorina citri* Kuwayama (Hemiptera: Liviidae)

Lixia Zheng[1,2]*, Wensheng Chen[1]

1 College of Horticulture and Food Sciences, Foshan University, Foshan, China, **2** College of Agronomy, Jiangxi Agricultural University, Nanchang, China.

*lxzheng@fosu.edu.cn; lxzheng@jxau.edu.cn (LZ)

Abstract

Diaphorina citri Kuwayama is the most economically important citrus pest which is the primary vector of *Candidatus* Liberibacter spp. causing citrus greening (huanglongbing, HLB) disease. To better understand the developmental and structural changes of antennae and antennal sensilla in *D. citri* nymphs ~~Because its ability to transmit *C.* Liberibacter was more efficient in nymphs~~, we investigated the antennal morphology, structure and sensilla distribution in five nymphal stages of *D. citri* using scanning electron microscopy. The antennae of five different nymphal stages were filiform in shape, which consisted of two segments in the first-, second- and third-instar nymphs; three segments in the fourth- and fifth-instar nymphs. The length of their antennae was significantly increased with the increase of the instars, as well as the total number of antennal sensilla. Eleven morphological sensilla types were recorded altogether. They were the long terminal hair (TH1), short terminal hair (TH2), sensilla trichoidea (ST), cavity sensillum 1 (CvS1), cavity sensillum 2 (CvS2), sensilla basiconica 1-4 (SB1-4), sensilla campaniform (SCA) and partitioned sensory organ (PSO). Also, the distribution of antennal sensilla in each nymphal stage of *D. citri* was asymmetrical. The SB only occurred on the antennae of the third-, fourth- and fifth-instar nymphs. Only one CvS2 was found in the third- and fifth-instar

nymphs, and one SCA in the fourth- and fifth-instar nymphs, respectively. The possible roles of the nymphal antennal sensilla in *D. citri* were discussed. The results could contribute to a better understanding of the development of the sensory system, and facilitate future studies on the antennal functions in *D. citri* nymphs.~~The results could contribute to further explore the relationship between the ability to carry and transmit *C.* Liberibacter and the feeding behavior of the nymphal *D. citri*.~~

Introduction

The Asian citrus psyllid (ACP), *Diaphorina citri* Kuwayama (Hemiptera: Liviidae), is the most economically important citrus pest because it is the primary vector of *Candidatus* Liberibacter spp. causing citrus greening (huanglongbing, HLB) disease, also the most serious citrus disease worldwide [1,2]. *Diaphorina citri* apparently originated in southern Asia, but now has spread to other world citrus-producing areas ~~of the world~~ [2]. More than 50 species of the family Rutaceae have been recorded as hosts of *D. citri* [3]. Nymphs and adults suck phloem sap from the foliage causing leaf distortion, curling and deposition of honeydew on leaves that result in mold growth [3,4]. However, the most serious threat to citrus worldwide comes from its ability to transmit the bacterium, *C.* Liberibacter spp., ~~the causal organism of~~ associated with HLB which causes yellowing of shoots, stem dieback, sour fruit, crop losses and eventually tree death [5,6]. ~~The nymphs and adults of *D. citri* feed on plant sap and the pathogen can only be transmitted by the third-, fourth- and fifth-instar nymphs and adults which play a major role in the spread of the pathogen because of their dispersal capabilities [2,7-9], but the nymphs were found to be more efficient [4]~~.

For most insects, the antennae are peripheral sensory structures involved in habitat searching, host location, host discrimination, mating and oviposition [710-912]. They carry different kinds of sensilla which can be classified as chemosensory, mechanosensory and thermo-hygroreceptive sensilla based on their morphological and functional characteristics [710,103]. Studies on the interaction between ACP and host plant volatiles indicated the critical role of its olfactory system in finding host

plants [114,125], mating [125-147], and oviposition~~spawning~~ [136,158]. Coutnho-Abreu et al. have recognized the ACP antennal neurons that responded strongly to odorants found in the host citrus plants using single-unit electrophysiology [169]. Recently, Zanadi et al. have identified the putative ACP sex pheromone [1720], in addition to other related compounds that also generated electroantennographic (EAG) responses [1720-1922]. Thus, to analyze the antennal morphology, structure and sensilla distribution in *D. citri* is important to explore their life cycle, olfactory behavior and host identification mechanisms.

Previous studies have described the external morphology and ultrastructure of antennal sensilla in adult *D. citri* [203], antennae sensory arrays in adult *D. citri* [214], antennal transcriptome in adult *D. citri* [225]. However, there is very little information about the morphology of nymphal antennae and antennal sensilla of psyllids. In this study, ~~Because its ability to transmit *C.* Liberibacter was more efficient in nymphs,~~ we investigated the antennal morphology, structure and sensilla distribution in five different nymphal stages of *D. citri* using scanning electron microscopy. The results could contribute to a better understanding of the development of the sensory system, and facilitate future studies on the antennal functions in ~~further explore the relationship between the ability to carry and transimit *C.* Liberibacter and the feeding behavior of~~ *D. citri* nymphs.

Materials and methods

Insects

Specimens used in the study were five different nymphal stages of *D. citri*. These insects originated from a colony maintained at Jiangxi Agriculture University at Nanchang, China (N28°45'36", E115°49'43") that was initiated from insects collected in South China Agriculture University at Guangzhou, China (N23°10'7", E113°21'27") in June 2016. The ACP were fed on *Murraya exotica* seedlings at 27 ± 1 ºC, 70 ± 5% and 14:10 L:D photoperiod.

Scanning electron microscopy (SEM)

The whole bodies of five different nymphal stages of *D. citri* were fixed in 2.5% glutaraldehyde at 4 ºC for 24 h and post-fixed in 1% osmium tetraoxide for 2.5 h. The treatment process for the specimens including rinsing, dehydrating and drying was according to Yang et al. [23~~6~~]. Finally, the specimens were anchored on a holder using double-sided adhesive tape in different orientations, sputter-coated with gold, examined and photographed either at 10 kV using an SEM (XL30, FEI, Holland and Nova Nano 430, FEI, Holland) or 25 kV by an SEM (JSM-6360LV, Japan).

Statistical analysis

Classification of sensilla types in this work was based on the morphological characteristics of similar structures described previously [24-32]~~ by Dethier [27], Schneider [28], Moran and Brown [29], Singleton-Smith et al. [30], Bromley et al. [31], Zacharuk [10], Zacharuk and Shields [32], Mellor and Anderson [33], Kristoffersen et al. [34], Onagbola et al. [23] and Zheng et al. [35]~~.

Statistical tests were performed with SPSS 17.0. Sensilla on the dorsal, ventral and two lateral surfaces of five different nymphal stages of *D. citri* antennae were counted and measured. Means (n=10) were analyzed by general linear model (GLM) procedure and Tukey's mean separation test. Mann-Whitney *U* test was used to compare the length and width of various stages between the long terminal hair (TH1) and short terminal hair (TH2).

Results

General morphology of antennae

The antennae of *D. citri* nymphs were found on~~A view from~~ the ventral side of the ~~insect~~ body~~, there was a pair of antennae observed~~ between the compound eyes~~ in nymphal *D. citri*~~. The ~~morpholog~~morphology~~y~~ of five different nymphal stages of *D. citri* antennae is illustrated in Fig 1. ~~The first, second and third instar nymphal~~

~~antennae were composed of two segments 换到第二段(Fig 1A-C), while the fourth- and fifth-instar nymphal antennae consisted of three segments (Fig 1D and 1E). There were s~~Significant differences were found in the length of the antennae of each nymphal stage of *D. citri* (Table 1). The length of the five different nymphal antennae was significantly increased with the increase of the nymphal instars.

First-instar nymph

The antennae of the first-instar nymphs were composed of two segments (Fig 1A). They were the shortest, measuring 61.85 ± 1.83 μm in length. There was no sensillum found on the first segment of the antennae (Se1). Only six antennal sensilla belonging to five morphologically different types were observed on the antennae of the first-instar nymphs. They were the TH1, TH2, sensilla trichoidea (ST), partitioned sensory organ (PSO) and cavity sensillum 1 (CvS1).

Second-instar nymph

The antennae of the second-instar nymphs were also composed of two segments (Fig 1B). They were 35.96 μm longer than the first-instar nymphal antennae (Table 1). The sensilla occurred on the first-instar nymphal antennae were also found in the second-instar nymphs. Moreover, there was ST found on the Se1 of the second-instar nymphs.

Third-instar nymph

The antennae of the third-instar nymphs were similar to those of the first- and second-instar nymphs, which were also composed of two segments (Fig 1C). The antennae of the third-instar nymphs were about 1.5 times longer than those of the second-instar nymphs (Table 1). Ten morphologically different types of sensilla were detected on the third-instar nymphal antennae. They were TH1, TH2, ST, PSO, four types of sensilla basiconica (SB1-4), CvS1 and CvS2.

Fourth-instar nymph

The antennae of the fourth-instar nymphs were composed of three parts: two basal segments and the flagellum with poorly defined subsegments (Fig 1D). The types of antennal sensilla in the fourth-instar nymphs were one less, but the total number was one more than those in the third-instar nymphs (Table 2). The SB4 and CvS2 were missed, however, the sensilla campaniform (SCA) was found in the fourth-instar nymphs.

Fifth-instar nymph

The antennae of the fifth-instar nymphs were similar to those of the fourth-instar nymphs, which were also composed of three parts (Fig 1F). They were the longest antennae in the five different nymphal stages of *D. citri* (Table 1). The types and the total number of antennal sensilla in the fifth-instar nymphs were the most (Table 2). Eleven morphologically different types of sensilla were detected on the fifth-instar nymphal antennae. They were TH1, TH2, ST, PSO, CvS1, CvS2, SB1-4 and SCA.

Morphology and structure of sensilla

Table 2 shows the antennal sensilla distributions in five different nymphal stages of *D. citri*. We observed the asymmetrical distribution of antennal sensilla in different nymphal stages. ST, TH1, TH2, PSO and CvS1 were found on the antennae of each nymphal stage of *D. citri*; CvS2 and SB4 only occurred on the antennae of the third- and fifth-instar nymphs, and one SCA was found in the fourth- and fifth-instar nymphs. In addition, SB1-3 was observed on the antennae of the third- fourth- and fifth-instar nymphs.

Formatted: Underline, Font color: Blue

Formatted: Font: 12 pt, Not Bold

Terminal hairs

There were two bristle-like sensilla apically on the terminal antennae of the five different nymphal stages (Fig 1 and 2A). The TH1 was strong and straight with

longitudinal grooves (Fig 2A). They were inserted into a big cuticular socket. The TH2 had a similar morphology to the TH1, but much shorter and stronger (Fig 2A and 2B, Table 3). The length of TH1 was significantly longer than that of the TH2 in each development stage. The lengths of TH1 and TH2 in the second-instar nymphs were the shortest, measuring 13.38 ± 0.66 μm and 9.88 ± 0.33 μm, respectively. In addition, the width of TH2 in the fifth-instar nymphs was significantly bigger than that in other nymphal stages and that of the TH1 (Table 3).–

Sensilla trichoidea

Sensilla trichoidea (ST) was found on the antennae of each nymphal stage of *D. citri*. They were straight hairs with a smooth surface and a big conical socket (Fig 3). Only one ST occurred on the Se1 of the second-, third- and fourth-instar nymphs. There was also only one ST positioned just below the base of the TH1 in each development stage (Fig 3A).

Partitioned sensory organ

Partitioned sensory organ (PSO) was located on the ventral side of the last ~~ill-defined~~ segment of the antennae of each nymphal stage of *D. citri*. The PSO formed with an open pit and a sheet structure from which a cavity sensillum protruded (Fig 4). The inner side of the PSO was enclosed by the cuticular fringe (Fig 4B). One large PSO was found in the first-instar nymphs, two in the second- and third-instar nymphs, three in the fourth-instar nymphs and four in the fifth-instar nymphs, respectively.

Cavity sensillum

Only one cavity sensillum 1 (CvS1) was found below the base of the TH2 in each nymphal stage. They were an oval sensory cavity formed by invaginations of the antennal cuticle and contained two sensilla (Fig 5A and S1 Fig). The cuticular parts of the two sensilla sitting on the bottom of the cavity were formed as short pegs,

terminating just below the antennal surface. The tips of two short pegs were flat, and one of them was flower-shaped.

Cavity sensillum 2 (CvS2) was only found on the ventral side of the base of the third segment of the fifth-instar nymphal antennae. Similar to the CvS1, they were a round sensory cavity containing only one sensilla peg with a flower-shaped tip (Fig 5B and 5C). The cavities were rounded, and the flower-shaped tip was not flat.

Sensilla basiconica

Only one sensilla basiconica 1 (SB1) was observed on the base of the Se1 of the third-, fourth- and fifth-instar nymphs. The SB1 was fitted into a tight socket with a sharp tip and was almost vertical to the longitudinal axis of the antennae (Fig 6A). It had a smooth surface, measuring 4.58 μm in length.

Sensilla basiconica 2 (SB2) was distributed on the dorsal side of the third-, fourth- and fifth-instar nymphal antennae. The SB2 was also fitted into a big socket elevated above the cuticle, measuring 6.35 μm in length and 1.73 μm in basal diameter. They displayed a longitudinally grooved surface with a sharp tip (Fig 6B).

Sensilla basiconica 3 (SB3), similar to the SB2, was also found on the dorsal side of the third-, fourth- and fifth-instar nymphal antennae. They were robust on the antennae, grew in a big basal socket, and measured 31.07 μm in length and 3.31 μm in basal diameter. The SB3 had multiple-pitted surfaces formed by clutter gullies, like the amygdala (Fig 6C). The number of SB3 was much more than that of the SB2 (Table 2).

Sensilla basiconica 4 (SB4) was sparsely distributed on the dorsal side of the last segment of the third- and fifth-instar nymphal antennae. Two SB4 were found in the third-instar nymphs, and one in the fifth-instar nymphs. The SB4 had a wavy line surface, like noodles (Fig 6D). They were also robust and grew in a big basal socket, measuring 12.7 μm in length and 3.86 μm in basal diameter.

Sensilla campaniform

A single sensilla campaniform (SCA) was found on the dorsal side of the second segment of the antennae~~Sc2~~ of the fourth- and fifth-instar nymphs (Fig 7). The SCA had a dome-shaped sensory structure. Their ambient cuticle was protuberant but sunken in the middle (Fig 7A). In addition, there was a concave spot at the sunken structure in SCA (Fig 7B).

Discus~~r~~sion

The morphology and ultrastructure of the antennae and antennal sensilla in different nymphal stages of *D. citri* were studied. The antennae of *D. citri* were composed of two segments in the first-, second- and third-instar nymphs, and two basal segments and the flagellum with poorly defined subsegments in the fourth- and fifth-instar nymphs (Fig 1). The length of the antennae of each development stage was significantly increased with the increase of the instars (Table 1), as well as the types and total number of the antennal sensilla (Table 2). At any stage of the life cycle of *D. citri*, the total number of antennal sensilla was small (Table 2 and S1 Table); moreover, their structure was rather simple. Eleven morphologically distinct types of sensilla were found~~ to distribute~~ on the antennae of five different nymphal stages of *D. citri* in our study. We do not find t~~T~~he SB1-4, CvS1 and CvS2 in adult *D. citri* (S1 File and S1 Table), as well as ~~were not reported by~~ Onagbola et al. [20~~3~~]~~ in adult *D. citri*~~, maybe they were degenerated and missed in adults. The PSO of the nymphal instars had a 'raised edges and internal shutter-like' appearance which might be replaced by the antennal rhinaria in adult *D. citri* (S1 File)~~(see S2 and S3)~~.

The TH were analogous with those found in *Psylla pyricola* Förster (Hemiptera: Psyllidae) [27~~30~~], *Trialeurodes vaporariorum* (Westwood) (Hemiptera: Aleyrodidae), *Aleyrodes proletella* (Linnaeus) (Hemiptera: Aleyrodidae), *Bemisia tabaci* (Gennadius) (Hemiptera: Aleyrodidae) [30~~3~~,33~~6~~], *Trioza apicalis* (Hemiptera: Triozidae) [31~~4~~], *Dialeurodes citri* (Ashmead) (Hemiptera: Aleyrodidae) [34~~7~~], *Aleurodicus dispersus* Russell (Hemiptera: Aleyrodidae) [32~~5~~,35~~8~~]. As is the case with other types of sensilla, the terminal hairs have been differently named by various authors. For example,

Singleton-Smith et al. [2730] and Mellor and Anderson [303] characterized them as “chaetica sensilla”, which can be suggested to serve as a proprioreceptor perceiving antennal movement and position or have mechano-sensory functions [203,369], due to their location and presence to membranous connections between the sensillar shaft and the antennal cuticular surfaces [3740]. In addition, considering the TH1 and TH2 in adults (Fig 2 in S1 File) that revealed wall pores suggesting a plausible role in perception of olfactory stimuli.

Trichoids hairs sensilla have been reported to perform either or both mechano- and chemo-sensory function [710]. The ST with no cellular material and pore canal structures (see S2 Fig4) resembled the mechanosensory hair which positioned just below the base of the TH1 in carrot psyllid [314]. In addition, the ST present in analogous locations with the AST-2 described by Onagbola et al. [203] may also suggest to have mechanosensory functions [203,314].

The PSO was first reported in *P. pyricola* and suggested chemoreceptors [2730]. Their morphology, location, and number were similar in five different nymphal instars between *P. pyricola* and *D. citri*. Previous studies have reported that the descriptions of the plate organs (sensilla placodea) in aphids conformed closest to the large sensory organ (PSO) [258,3841-403]. Therefore, if these were indeed plate organs that were placed in the category of chemoreceptor by Slifer [403], they could be involved in olfactory function.

The CvSs wereas the compound sensilla which consisted of a sensory cavity and the sensilla pegs, but they were only meant the sensory cavity named by Kristoffersen et al. [314] and Onagbola et al. [203] in psyllids. The difference between CvS1 and CvS2 was the number of pegs in the cavity. All of their pegs were well hidden beneath the antennal surface, and their position was far from optimal for receiving chemical stimuli. Kristoffersen et al. [314] have reported that these pegs are likely to be a necessary adaptation to prevent desiccation. Moreover, several studies have demonstrated that sensilla that are recessed from the antennal surface and located within cavities may be involved in perception of the CO_2, humidity, and temperature [414-436]. Judging by morphological features, the CvS1 and CvS2 could be

responsible for the perception of any type of stimuli as described above.

The SBs only occurred on the antennae of third-, fourth- and fifth-instar nymphs. Similarly, they were not reported in other psyllids, no matter nymphs or adults [203,2730,314]. In our study, the SBs wereas inserted into a big inflexible socket and exhibited different morphological surfaces (Fig 6). Judging by the morphological surface features, four subtypes of this sensillum can be identified. The SB3 and SB4 were the reported. According to Schneider [258], sensilla basiconica are the most common and important chemoreceptors found on the antennae of insects. Moreover, Altner and Prillinger [447] and Zacharuk [458] suggested that it would be impossible for sensilla with an inflexible socket to act as a mechanoreceptor.

The SCA has been found in many insects, including whiteflies [369] and psyllids [2730]. On the antennae of the fourth- and fifth-instar nymphs, only one SCA was presented on the second segment of the antennaeSe2. The SCA was seemed to occur in aging nymphs (the fourth-instar nymph of *A. dispersus* and the fifth-instar nymph of *P. pyricola*) [2730,325] or adults (*Tetrastichus howardi* and *A. dispersus*) [358,469]. Previous studies have reported that SCA with no pores in their cuticular structure play the role of mechanoreceptors [2932,3740,4750-514], whereas the SCA with pores was involved in gustatory function were highly susceptible to humidity [525]. Here they probably acted as mechanoreceptors, because they were few and located near the segmental joints [710,269,536,547].

In our study, the types and total number of the antennal sensilla obviously increased in the third-, fourth- and fifth-instar nymphs. According to Hung et al. [557], *C.* Liberibacter asiaticus (Las) persists in the ACP vector but is not transovarially transmitted by the vector. The pathogen can only be transmitted by the third-, fourth- and fifth-instar nymphs and adults which play a major role in the spread of the pathogen because of their dispersal capabilities [2,55-57], but the nymphs were found to be more efficient [4]. Only the third-, fourth- and fifth-instar nymphs and adults can carry and transmit Las, and Las in these nymphs can transmit to the adults. In our study, the types and number of the antennal sensilla increased in the third-, fourth- and fifth-instar nymphs. Further studies are needed to determine whether the antennal

Formatted: Underline, Font color: Blue

sensilla in the third-, fourth- and fifth-instar nymphs (especially the SBs) are related to the feeding behavior on the citrus with Las by these nymphs. In conclusion, we have provided an extensive description of the antennae and antennal sensilla in different nymphal stages of *D. citri* using SEM. This information can be a great help for revealing the developmental course of psyllid's antennae and antennal sensilla and allow us to better understand the host location mechanism in *D. citri*.

Acknowledge

The authors are grateful to Wei-Chun Li from Jiangxi Agricultural University for English revision and critical reading of the manuscript. We would like to thank Ms Xiao-Yin Hu from the Centre Laboratory of the South China Botanical Garden for her valuable assistance with the treatment for the specimens in SEM. Special thanks to Wei-Jian Wu from South China Agricultural University for statistical analysis.

Reference

1. ~~1.~~ Bové JM. Huanglongbing: a destructive, newly-emerging, century-old disease of citrus. Journal of Plant Pathology. 2006; 88: 7-37.

~~1.~~2. Grafton-Caldwell EE, Stelinski LL, Stansly PA. Biology and management of Asian citrus psyllid, vector of the Huanglongbing pathogens. Annual Review of Entomology. 2013; 58: 413-432.

~~2. Grafton-Caldwell EE, Stelinski LL, Stansly PA. Biology and management of Asian citrus psyllid, vector of the Huanglongbing pathogens. Ann Rev Entomol. 2013; 58: 413-432.~~

3. Halbert SE, Manjunath KL. Asian citrus psyllids (Sternorrhyncha: Psyllidae) and greening disease of citrus: a literature review and assessment of risk in Florida. Fla Entomol. 2004; 87: 330-353.

4. Ammar ED, Alessandro R, Shatters JrRG, Hall DG. Behavioral, ultrastructural and chemical studies on the honeydew and waxy secretions by nymphs and adults of Asian citrus psyllid *Diaphorina citri* (Hemiptera: Psyllidae). PloS One. 2013;

Formatted: Font: Times New Roman, 12 pt

Formatted: Indent: First line: 0 ch

Formatted: Font: Times New Roman, 12 pt

Formatted: Font: Times New Roman, 12 pt

8(6): e64938.

5. McClean PD, Schwarz RE. Greening or blotchy mottle disease of citrus. Phytophylactica. 1970; 2: 177-194.

6. Dagulo L, Danyluk MD, Spann TM, Valim MF, Goodrich-Schneider R, Sims C, et al. Chemical characterization of orange juice from trees infected with citrus greening (Huanglongbing). Journal of Food Science. 2010; 75(2): C199-C207.

~~7. Hung TH, Hung SC, Chen CN, Hsu MH, Su HJ. Detection by PCR of *Candidatus* Liberibacter asiaticus, the bacterium causing citrus huanglongbing in vector psyllids: application to the study of vector pathogen relationships. Plant Pathol. 2004; 53: 96-102.~~

~~8. Boina DR, Rogers ME, Wang N, Stelinski LL. Effect of pyriproxyfen, a juvenile hormone mimic, on egg hatch, nymph development, adult emergence and reproduction of the Asian citrus psyllid, *Diaphorina citri* Kuwayama. Pest Manag Sci. 2010; 66: 349-357.~~

~~9. Cen Y, Yang C, Holford P, Beattie GAC, Spooner-Hart RN, Liang G, et al. Feeding behaviour of the Asiatic citrus psyllid, *Diaphorina citri*, on healthy and hunaglongbing infected citrus. Entomol Exp Applic. 2012; 143: 13-22.~~

7~~10~~. Zacharuk RY. Antennae and sensilla. In: Kerkut GA, Gilbert LJ, editors. Comprehensive Insect Physiology, Biochemistry, and Pharmacology, Sensory. Oxford: Pergamon; 1985. p.1-69.

8~~11~~. Bland RG. Antennal sensilla of acrididae (Orthoptera) in relation to subfamily and food preference. Annals of the Entomological Society of America. 1989; 82: 368-384.

9~~12~~. Blaney WM, Simmonds MSJ. The chemoreceptors. In: Chapman RE, Joern A, editors. Biology of Grasshoppers. New York: Wiley; 1989. p.1-37.

10~~3~~. Gullan PJ, Cranston PS. The Insects: an Outline of Entomology, third ed. London: Blackwell Publishing Ltd; 2004. p. 86-105.

11~~4~~. Patt JM, Sétamou M. Responses of the Asian citrus psyllid to volatiles emitted by flushing shoots of its rutaceous host plants. Environmental Entomology. 2010;

39: 618-624.

12~~5~~. Moghbeli Gharaei A, Ziaaddini M, Jalali MA, Michaud JP. Sex-specific responses of Asian citrus psyllid to volatiles of conspecific and host-plant origin. Journal of Applied Entomology. 2014; 138(7): 500-509.

13~~6~~. Wenninger EJ, Hall DG. Daily timing of mating and age at reproductive matruity in *Diaphorina citri* (Hemiptera: Psyllidae). Florida~~a~~ Entomologist. 2007; 90: 715-722.

14~~7~~. Wenninger EJ, Stelinski LL, Hall DG. Behavioral evidence for a female-produced sex attractant in *Diaphorina citri* (Hemiptera: Psyllidae). Entomol Exp Appl. 2008; 128: 450-459.

15. Moran VC, Buchan PR. Oviposition by the citrus psylla, *Trioza erytreae* (Homoptera: Psyllidae), in relation of leaf hardiness. Entomologia Experimental et Applicata. 1975; 18: 96-104.

~~18. Moran VC, Buchan PR. Oviposition by the citrus psylla, *Trioza erytreae* (Homoptera: Psyllidae), in relation of leaf hardiness. Entomol Exp Appl. 1975; 18: 96-104.~~

16~~9~~. Coutinho-Abreu IV, McInally S, Forster L, Luck R, Ray A. Odor coding in a disease-transmitting herbivorous insect, Asian citrus psyllid. Chemical Senses. 2014; 39: 539-549.

17~~20~~. Zanardi OZ, Volpe HXL, Favaris AP, Silva WD, Luvizotto RAG, Magnani RF, et al. Putative sex pheromone of the Asian citrus psyllid, *Diaphorina citri*, breaks down into an attranctant. Scientific Reports. 2018; 8(1): 455.

18~~21~~. George J, Robbins PS, Alessandro RT, Stelinski LL, Lapointe SL. Formic and acetic acids in degradation products of plant volatiles elicit olfactory and behavioral reponses from an insect vector. Chemical Senses. 2016; 41: 325-338.

19~~22~~. Zanardi OZ, Volpe HXL, Luvizotto RAG, Magnani RF, Gonzalez F, Calvo C, et al. Laboratory and field evaluation of acetic acid-based lures for male Asian citrus psyllid, *Diaphorina citri*. Scientific Reports. 2019; 9(1): 1-10.

20~~3~~. Onagbola EO, Meyer WL, Boina DR, Stelinski LL. Morphological characterization of the antennal sensilla of the Asian citrus psyllid, *Diaphorina*

citri Kuwayama (Hemiptera: Psyllidae), with reference to their probable functions. Micron. 2008; 39(8): 1184-1191.

21~~4~~. Arras J, Hunter W, Bextine B. Comparative analysis of antennae sensory arrays in Asian citrus psyllid, *Diaphorina citri*, and potato psyllid, *Bactericera cockerelli* (Hemiptera). Southwestern Entomologist. 2012; 37(1): 1-12.

22~~5~~. Wu ZZ, Zhang H, Bin SY, Chen L, Han QX, Lin JT. Antennal and abdominal transcriptomes reveal chemosensory genes in the Asian citrus psyllid, *Diaphorina citri*. PloS One. 2016; 11(7): e0159372.

23~~6~~. Yang HY, Zheng LX, Zhang ZF, Zhang Y, Wu WJ. The structure and morphologic ~~—~~changes of antennae of *Cyrtorhinus lividipennis* (Hemiptera: Miridae: ~~—~~Orthotylinae) in different instars. PloS One. 2018; 13(11): e0207551.

24~~7~~. Dethier VG. The physiology of insect senses. London: Methuen and Co. Ltd; 1963.

25~~8~~. Schneider D. Insect antenna. Annual Review of Entomology.1964; 9: 103-122.

26~~9~~. Moran VC, Brown RP. The antennae, host plant chemoreception and probing activity of citrus psylla, *Trioza erytreae* (Del Guercio) (Homoptera: Psyllidae). Journal of the Entomological Society of Southern Africa. 1973; 36(2): 191-202.

27~~30~~. Singleton-Smith J, Chang JF, Philogène JR. Morphological differences between nymphal instars and descriptions of the antennal sensory structures of the nymphs and adults of *Psylla pyricola* Foerster (Homoptera: Psyllidae). Canadian Journal of Zoology-Revue Canadienne de Zoologie. 1978; 56(7): 1576-1584.

28~~31~~. Bromley AK, Dunn JA, Anderson M. Ultrastructure of the antennal sensilla of aphids I. coeloconic and placoid sensilla. Cell and Tissue Research. 1979; 203: 427-442.

29~~32~~. Zacharuk RY, Shields VD. Sensilla of immature insects. Annual Review of Entomology. 1991; 36: 331-354.

30~~3~~. Mellor HE, Anderson M. Antennal sensilla of whiteflies: *Trialeurodes vaporariorum* (Westwood), the glasshouse whitefly, *Aleyrodes proletella* (Linnaeus), the cabbage whitefly, and *Bemisia tabaci* (Gennadius), the tobacco

whitefly (Homoptera: Aleyrodidae). Part 1: external morphology. International Journal of Insect Morphology and Embryology. 1995; 24(2): 133-143.

314. Kristoffersen L, Hallberg E, Wallén R, Anderbrant O. Sparse sensillar array on *Trioza apicalis* (Homoptera, Triozidae) antennae——an adaptation to high stimulus levels? Arthropod Structure and Development. 2006; 35: 85-92.

325. Zheng LX, Wu WJ, Liang GW, Fu YG. Nymphal antennae and antennal sensilla in *Aleurodicus dispersus* (Hemiptera: Aleyrodidae). Bulletin of Entomological Research. 2014; 104: 622-630.

336. Zhang XM, Wang S, Li S, Luo C, Li YX, Zhang F. Comparison of the antennal sensilla ultrastructure of two cryptic species in *Bemisia tabaci*. PLoS One. 2015; 10(3): e0121820.

347. Zheng LX, Xiao TT, Wu WJ, Cui ZX, Fu YG. Ultra-structure observation on the antenna sensilla of adult citrus whitefly *Dialeurodes citri*. Journal of Fruit Science. 2011; 28(2): 344-347.

358. Zheng LX, Wu WJ, Fu YG. Sensilla on antenna of adult spiraling whitefly *Aleurodicus dispersus*. Chinese Bulletin of Entomology. 2010; 47(3): 525-528.

369. Ochieng SA, Park KC, Zhu JW, Baker TC. Functional morphology of antennal chemoreceptors of the parasitoid *Microplitis croceipes* (Hymenoptera: Braconidae). Arthropod Structure and Development. 2000; 29: 231-240.

3740. Chapman RF, Chapman RF. The insects: structure and function, Cambridge University Press; 1998.

3841. Slifer EH, Sekhon SS, Lees AD. The sense organs on the antennal flagellum of aphids (Homoptera), with special reference to the plate organs. Quarterly Journal of Microscopical Science. 1964; 105: 21-30.

3942. Imms AD. A general textbook of entomology, ninth ed. London: Methuen and Co. Ltd; 1970.

403. Slifer EH. The structure of arthropod chemoreceptors. Annual Review of Entomolpgy. 1970; 15: 121-142.

414. Steinbrecht RA. Arthropods: Chemo-, thermo-, and hygro-receptor. In: Bereiter-Hahn J, Matolsty AG, Richards KS, editors. Biology of the intergument,

Vol. 1. Berlin: Spring-Verlag; 1984. p. 523-553.

425. Stange G, Stowe S. Carbon-dioxide sensing structures in terrestrial arthropods. Microscopy Research and Technique. 1999; 17: 416-427.

436. Onagbola EO, Fadamiro HY. Scanning electron microscopy studies of antennal sensilla of *Pteromalus cerealellae* (Hymenoptera: Pteromalidae). Micron. 2008; 39: 526-535.

447. Altner H, Prillinger L. Ultrastructure of invertebrate chemo-, thermo-, and hygroreceptors and its functional significance. International Review of Cytology. 1980; 67: 69-139.

458. Zacharuk RY. Ultrastructure and function of insect chemosensilla. Annual Review of EntomolpgyAnnu Rev Entomol. 1980; 25: 27-47.

469. Zheng YH, Zheng LX, Liao YL, Wu WJ. Sexual dimorphism in antennal morphology and sensilla ultrastructure of a pupal endoparasitoid *Tetrastichus howardi* (Olliff) (Hymenoptera: Eulophidae). Microscopy Research and Technique. 2016; 79: 374-384.

4750. Slifer EH, Sekhon SS. Fine structure of the sense organs on the antennal flagellum of the honey bee, *Apis mellifera*. Linnaeus. Journal of Morphology. 1961; 109: 351-381.

4851. Stort CA, Barelli N. Genetic study of olfactory structures in the antennae of two *Apis mellifera* subspecies. Journal of the Kansas Entomological Society. 1981; 54: 352.

4952. Hashimoto Y. Unique features of sensilla on the antennae of Formicidae (Hymenoptera). Journal of Applied Entomology and Zoology. 1990; 25: 491-501.

503. Olson DM, Andow DA. Antennal sensilla of female *Trichogramma nubilale* (Ertle and Davis) (Hymenoptera: Trichogrammatidae) and comparisons with other parasitic Hymenoptera. International Journal of Insect Morphology & Embryology. 1993; 22: 507-520.

514. Kleineidam C, Romani R, Tautz J, Izidoro N. Ultrastructure and physiology of the CO_2 sensitive sensillum ampullaceum in the leaf-cutting ant *Atta sexdens*.

Arthropod Structure and& Development. 2000; 29: 43-55.

525. Dietz A, Humphreys WJ. Scanning electron microscopic studies of antennal receptors of the worker honey bee, including sensilla campaniformia. Annals of the Entomological Society of America. 1971; 64: 919-925.

536. McIver SB. Mechanoreception. In: Kerkut GA, Gilbert LI, editors. Comparative Insect Physiology, Biochemistry and Pharmacology, Pergamon Press, Oxford; 1985. p. 71-132.

547. Liang AP, Fletcher JM. Morphology of the antennal sensilla in four Australian spittlebug species (Hemiptera: Ceropidae) with implications for phylogeny. Australian Journal of Entomology. 2002; 41: 39-44.

55. Hung TH, Hung SC, Chen CN, Hsu MH, Su HJ. Detection by PCR of *Candidatus* Liberibacter asiaticus, the bacterium causing citrus huanglongbing in vector psyllids: application to the study of vector pathogen relationships. Plant Pathology. 2004; 53: 96-102.

56. Boina DR, Rogers ME, Wang N, Stelinski LL. Effect of pyriproxyfen, a juvenile hormone mimic, on egg hatch, nymph development, adult emergence and reproduction of the Asian citrus psyllid, *Diaphorina citri* Kuwayama. Pest Management Science. 2010; 66: 349-357.

57. Cen Y, Yang C, Holford P, Beattie GAC, Spooner-Hart RN, Liang G, et al. Feeding behaviour of the Asiatic citrus psyllid, *Diaphorina citri*, on healthy and hunaglongbing infected citrus. Entomologia Experimental et Applicata. 2012; 143: 13-22.

Supporting information

S1 Fig. Transmission electron microscopy micrograph of the transect through the cavity sensillum 1 of the first-instar *Diaphorina citri*, showing the two sensilla in the cavity (arrows).

(DOCX)

S2 Fig. Transmission electron microscopy micrograph of the sensilla trichoidea positioned below the base of the TH1 in the third-instar *Diaphorina citri* nymphs.General morphology of antennae of adult *D. citri*. (A) SEM photograph of the dorsal view of female *D. citri* antennae, showing the scape, pedicel, and flagellum;

~~(B) SEM photograph of the ventral view of male *D. citri* antennae. F1, F2, F3, F4, F5, F6, F7, and F8 mean the eight flagella subsegments comprised of the flagellum; AR, antennal rhinarium; TH1, the long terminal hair; TH2, the short terminal hair.~~

(DOCX)

S1 Table. Abundance and distribution of sensilla on the antennae of adult *Diaphorina citri*.~~**S3 Fig. Antennal rhinarium (AR) in males.** (A) Antennal rhinarium, showing the cuticular fringe. (B) Antennal rhinarium, showing the pores.~~

(DOCX)

S1 File. General analysis of the antennae and antennal sensilla of adult *Diaphorian citri*.~~**S4 Fig. Transmission electron microscopy micrograph of the sensilla trichoidea positioned below the base of the TH1 in the third instar nymphs.**~~

(DOCX)

Table 1. Length (mean ± SE) of various nymphal stages of *Diaphorina*~~*D.*~~ *citri* antennae.

Formatted: Justified

Nymphal stages	*N*	Length (μm)
First-instar	10	61.85 ± 1.83e
Second-instar	10	97.81 ± 1.98d
Third-instar	10	150.83 ± 4.39c
Fourth-instar	10	188.38 ± 20.69b
Fifth-instar	10	316.11 ± 3.22a

Means with same letters in the same column are not significantly different (GLM, Tukey, *P*>0.05).

Table 2. Abundance and distribution of sensilla on the antennae of various

Formatted: Justified

nymphal stages of *Diaphorina~~D.~~ citri*.

Nymphal stages		Sensilla											Total	
		ST	TH1	TH2	PSO	CvS1	CvS2	SCA	SB1	SB2	SB3	SB4		
First-instar	Se1	—	—	—	—	—	—	—	—	—	—	—	—	6
	Se2	2	1	1	1	1	—	—	—	—	—	—	6	
Second-instar	Se1	1	—	—	—	—	—	—	—	—	—	—	1	9
	Se2	3	1	1	2	1	—	—	—	—	—	—	8	
Third-instar	Se1	1	—	—	—	—	—	—	1	—	—	—	2	17
	Se2	3	1	1	2	1	1	—	—	1	3	2	15	
Fourth-instar	Se1	1	—	—	—	—	—	1	1	—	—	—	3	18
	Se2	—	—	—	—	—	—	—	—	1	1	—	2	
	Se3	4	1	1	3	1	—	—	—	1	2	—	13	
Fifth-instar	Se1	4	—	—	—	—	—	1	1	—	—	—	6	31
	Se2	3	—	—	—	—	—	—	—	—	3	—	6	
	Se3	5	1	1	4	1	1	—	—	3	2	1	19	

Formatted Table

Number and location of the various sensilla observed on the antennae of various nymphal stages of *D. citri*. Se1, Se2 and Se3,~~:~~ the first, second and third segment of the antennae ~~segment 1, 2 and 3~~; ST,~~:~~ sensilla trichoidea; TH1 and TH2,~~:~~ the long and short terminal hair at the tip of the antennae, respectively; PSO,~~:~~ partitioned sensory organ; CvS1 and CvS2,~~:~~ cavity sensillum 1 and 2, respectively; SCA,~~:~~ sensilla campaniform; SB1, SB2, SB3 and SB4,~~:~~ sensilla basiconica 1, 2, 3 and 4, respectively; '—', indicates absent.

Table 3. Length and width (mean ± SE, *n*=10) of TH1 and TH2 of various nymphal stages of *Diaphorina*~~*D.*~~ *citri*.

Stages	Length (µm)		W~~w~~idth(µm)	
	TH1	TH2	TH1	TH2
First-instar	16.03±0.62ABa	13.60±0.68ABb	2.16±0.14Ca	1.99±0.14Ca
Second-instar	13.38±0.66Ba	9.88±0.33Bb	2.37±0.20Ca	2.45±0.09Ca
Third-instar	16.34±0.85ABa	10.30±0.83Bb	2.53±0.09Ca	2.73±0.19BCa
Fourth-instar	18.87±1.33ABa	13.00±0.51ABb	2.90±0.25BCa	3.65±0.30Ba
Fifth-instar	21.89±1.32Aa	15.13±1.23Ab	4.05±0.49ABa	4.81±0.26Ab

Means in columns with same uppercase letters are not significantly different (GLM, Tukey, *P*>0.05). Means in rows with same lowercase letters are not significantly different (*P*>0.05) in Mann–Whitney *U* test.

Formatted: Justified

Formatted: Font: Not Italic

Formatted: Font: Bold

Formatted: Font: Bold

We have studied the comments carefully and have made correction which we hope meet with approval. The main corrections in the paper and the responds to the comments are as flowing:

Responds to Reviewer 1 (file "review"):

Zheng et al observed the antennae of the *Diaphorina citri* nymph, and identified the sensilla on the antennae with scanning electron microscopy. The general morphology difference was clarified in different nymph stages. The number, distribution, ultrastructure of the sensilla (11 types) on the nymph antennae was recorded. The function of these sensilla was deduced by comparing with the sensilla of other insect species to explore their role of in Candidatus Liberibacter transmitting and *D. citri* nymph feeding. The results is very interesting. However, this manuscript need some improvements to publish, especially the quality of the photo. It is difficult to get useful information without clear photo showing the characteristic of the sensilla.

1 in the method section of page 3 (line 82-83), why the author used the double-sided adhesive tape, but not a carbon conductive tape. In line 84, the author said they used a XL30, and Nova Nano 430 scanning electron microscopy at 10 kV. In figure 5,B, the photo labeled with 25kV.

Response: Thank you very much for your carefulness. We have performed the studies of SEM both at South China Agriculture University and Centre Laboratory of the South China Botanical Garden several times, but the treatment method and process of the specimens are the same. Most of the SEM photos were got at South China Agriculture University using an SEM (XL30, FEI, Holland and Nova Nano 430, FEI, Holland) at 10kV, few SEM photos were got at Centre Laboratory of the South China Botanical Garden using an SEM (JSM-6360LV, Japan) at 25kV. The reason for the specimens were used the double-sibed adhesive tape, actually, we don't know why. The method of anchoring the specimens was provided by the researchers in these two electron microscope laboratories.

2 in line 94, page 4, the length and width of various stages of long terminal hair and short terminal hair were determined. How they measured the length and the width. How many insects they measured. How to minimize the measurement differences due to the viewing angles.

Response: Thank you very much for your comments. The length and width (basal diameter) of the long terminal hair and short terminal hair are measured from the printed SEM images by a slide caliper. The starting and ending points of the measured data are all in the middle. Ten insects of each stage of *D. citri* were examined.

3 I suggest to mark the segments of the antennae in figure 1. For example, it is difficult to find any sensillum on the second- instar nymph without any mark in figure 1A (line 115) and 1B (line 122).

Response: Thank you very much for your suggestion. The first and second segments of the antennae in five different nymphal stages are segmented clearly, and we have marked for the fourth-instar in Fig 1D. In the first- and second-instar nymphs, the total number of antennal sensilla is very small; moreover, they were distributed either on the ventral side or on the dorsal of the antennae. So, we cannot observe all of them in one picture.

4 All the photos are blurry (especially the photo taken by Nova Nano 430) and it is recommended to shoot again. A good result maybe obtain by Nova Nano 430 at low voltage (for example 5Kv) without dehydrating and drying of the insect.

Response: Thank you very much for your suggestion. In order to get the high quality photos, we performed our SEM study both at South China Agriculture University and Centre Laboratory of the South China Botanical Garden several times. We even tried to take the specimens without any treatments to anchor for examining by SEM. Here, we hope our photos may meet the requirement of PLoS One.

5 What is the difference between terminal hair and TH1 and TH2 (in paragraph 2, page 6). How to determine the TH1 is longer than TH2, rather than the visual illusion caused by view angle.

Response: Thank you very much for your comments. TH1 was distributed with a ST on the antennal tip of different instars of *D. citri* (see Fig 3), including adult females and males (see Fig 1 and Fig 2A in S1 File). In *D. citri* nymphs, we don't find the morphological difference between TH1 and TH2 except the length and basal diameter (width). However, there is the obvious difference found in adults (see Fig 3 in S1 File). Moreover, the length of TH1 and TH2 was significant difference in the length and width (see Table 2 in S1 File).

6 What is the last ill-defined segment and partitioned sensory organ? (line 171,page 7). A concise legend is needed for figure 4 to help it stand alone (useful information can be found here, https://blog.bioturing.com/2018/05/10/how-to-craft-a-figure-legend-for-scientific-papers/)

Response: Thank you very much for your comments. Partitioned sensory organ was located on the last segment of the antennae of each ACP nymphal stage. The fourth- and fifth-instar nymphs had an ill-defined flagellum which was also the last segment of the antennae. Our expression is not very accurate, and we have revised it. Also, we have checked all of our figure legends, and thank you very much.

7 What is the cavity sensillum, where it come from?

Response: Thank you very much for your comment. Cavity sensillum consisted of a sensory cavity and one (Fig 5B) or two pegs (Fig 5A). I have marked the cavity sensillum with a circle, maybe it can be easy to understand. I am very sorry that I don't really understand that "where it come from?" All sensilla in our study were distributed on the antennae of five different

nymphal stages of *D. citri*.

8 In the discussion section, paragraph 2, the author claimed that another name of TH is chaetica sensilla, why?

Response: In most of whiteflies and psyllids such as *Trialeurodes vaporariorum*, *Aleyrodes proletella*, *Bemisia tabaci*, *Dialeurodes citri*, *Aleurodicus disperses*, *Psylla pyricola*, *Trioza apicalis* and *D. citri*, there are one or two TH found on the antennal tip. According to Singleton-Smith et al. (1978) and Mellor and Anderson (1995), they are characterized as “chaetica sensilla”, maybe due to their location and the morphology of that they are obvious strong and long with longitudinal groove surface and inserted into a big cuticular socket. These antennal sensilla should be the first to touch and perceive the external environment because of their location and size.

(Singleton-Smith J, Chang JF, Philogène JR. Morphological differences between nymphal instars and descriptions of the antennal sensory structures of the nymphs and adults of *Psylla pyricola* Foerster (Homoptera: Psyllidae). Can J Zool. 1978; 56(7): 1576-1584.)

(Mellor HE, Anderson M. Antennal sensilla of whiteflies: *Trialeurodes vaporariorum* (Westwood), the glasshouse whitefly, *Aleyrodes proletella* (Linnaeus), the cabbage whitefly, and *Bemisia tabaci* (Gennadius), the tobacco whitefly (Homoptera: Aleyrodidae). Part 1: external morphology. International Journal of Insect Morphology and Embryology. 1995; 24(2): 133-143.)

9 The reference format should be checked carefully, and keep them in consistent. 2,3, 5,17, 18,,24,37,39,41 44,

Response: Done, thank you very much.

Special thanks to you for your comments and suggestions.

Responds to Reviewer 2 (from the decision letter):

1 The manuscript suffers from the lack of an appropriate structural origination. Specifically, in the results section, while the first part focuses mainly on the nymphal instars the authors also deal solely with the presence (or absence) and distribution of various types of the sensilla without first presenting their characteristics. This definitely confuses the readers. I'd suggest the authors rewrite the results section by combining the second part of the results i.e. "Morphology and Structure of Sensilla" with the first part so that in each paragraph the description of each sensilla type should be followed by its presence (or absence), number, distribution, etc., in each nymphal stage. The authors are also encouraged to better formulate their hypothesis in the introduction (the information is there but is not very well promoted!).

Response: Thank you very much for your suggestion. We tried to revise our MS according to your suggestion, but we found a little messy after adjusting the part of 'Result' of MS layout. We have revised our MS carefully according to the comments, changed the argument used to justify the choice for nymphal stages of *D. citri*, and added some description to improve the structure and quality of our MS, such as lines 147-153.

2 Lack of TEM and/or electrophysiological studies: Usually studies of this kind are accompanied by TEM and/or electrophysiological experiments for strengthening the results. I understand that due to, e.g. lack of equipment or budget constraints, it might not be feasible to conduct the above-mentioned experiments. Hence, I'd suggest that …

Response: Thank you very much for your good suggestion, we also try to get some high quality TEM photographs. From March 2017 to September 2018, we have performed the studies of SEM and TEM at South China Agriculture University and Centre Laboratory of the South China Botanical Garden, moreover, at Institute of Food Science and Technology CAAS for TEM. Unfortunately, we did not get good TEM photographs of different nymphal

stages of *Diaphorina citri*. For the electrophysiological studies of *D. citri* nymphs, we have not plan to do, but we have done the adult electrophysiological studies. ACP is a small insect, and it is not easy to connect the antennae in the adult electrophysiological studies. Moreover, the various nymphal stages of *D. citri* antennae are much shorter than the adults (see Table 1 in S1 File), especially the first-, second-, third- and fourth-instar nymphal antennae (Table 1). Thank you very much for your suggestion, it is very helpful for our future researches.

3 … the authors submit their findings to other journals (such as Micron) that publish these kinds of works so as to attract more readers interested in the SEM.

Response: Thank you very much for your suggestion, most of our teams' works about the SEM or TEM have published in Micron, Microscopy Research and Technique, as well as Plos One. ACP is the most important insect, and there are also quite a few researches about the ACP published in Plos One. We also think it is appropriate to submit our MS to Plos One.

4 I believe the following items will increase the value of the manuscript: 1) Inclusion of parameters like the size of individual flagellomeres across the nymphal instars. 2) Figuring out if there is a correlation between the nymph size within each nymphal stage and the number of sensilla? 3) Were the individuals size-matched? I'd suggest the authors include the average head-width (indicative of size of the nymphs) of each nymphal stage in table 1.

Response: 1) Thank you very much for your suggestions. It is very valuable and helpful for the important guiding significance to our future researches. 2) The length of the five different nymphal antennae was significantly increased with the increase of the nymphal instars, as well as the number of sensilla. We do not find the correlation between the nymph size within each nymphal stage and the number of sensilla. 3) Yes, it is size-matched.

The classification characteristics of different stages of ACP nymphs are according to Ruan et al. (2012). The SEM photographs were just only antennae, not containing the whole head, so, it could not measure the ACP head-width of each nymphal stage. Thank you very much for your suggestions.

(Ruan CQ, Chen JL, Liu B, Duan YP, Xia YL. 2012. Morphology and behavior of Asian citrus psyllid, *Diaphorina citri* Kuwayama. Chinese Agricultural Science Bulletin, 28(31): 186-190)

5 Lines 14-15: "Because its ability to transmit C. Liberibacter was more efficient in nymphs" please change it to "Because the nymphal instars have been reported to more efficiently transmit C. Liberibacter".

Response: Done, thank you very much.

6 Line 25: "The distribution of antennal sensilla in each nymphal stage of *D. citri* was asymmetrical". Do you mean they are "randomly distributed"?

Response: No, it just means that the distribution of antennal sensilla in nymphs was not the same. The SBs, CvS2 and SCA are the examples of how the antennal sensilla in nymphs asymmetrically distribute. These sensilla are not distributed in each nymphal stage, only in the older nymphs such as third-, fourth- and fifth-instar.

7 Lines 93-97: Under statistical analysis, instead of clearly explaining how they analyzed their data, the authors refer their readers to 11 references that focus on the classification of the sensilla. Please remove the author names and instead write, "…similar structures described previously [27-35].

Response: Done, thank you very much.

8 When the antennae are mounted only one side of the antennae is facing upward. It is

not clear how the number of different sensilla were counted. Please elaborate on that.

Response: Done, thank you very much. Each side of the antennae (dorsal, ventral, and two lateral surfaces of different nymphal stages of *D. citri*) was mounted to obverse. Each measured parameters was ten, and we have added the relevant contents in lines 90-92.

9 Figure legends must be self-explanatory. For example in figure 1 it must be clearly explained that A-C belong to the 1st through the 3rd nymphal instars with two antennal flagellomeres and D-E belongs to the 4th and 5th nymphal with three flagellomeres.

Response: Done, thank you very much.

10 Lines 139-140: basal segments and subsegments must be indicated in the figure.

Response: Done, thank you very much.

11 “Se 1” or “S1”? Please be consistent.

Response: I am very sorry for using such similar abbreviation. Actually, “Se1” is the abbreviation of the first segment of antennae, while “S1” is the abbreviation of the “S1 Fig.” in the file “Supplementary data”. We have renamed the supplementary.

12 Line 244: please change “trichoids hairs” to “trichoid sensilla”

Response: Done, thank you very much.

Special thanks to you for your comments and suggestions.

Responds to Reviewer 3 (from the decision letter):

1 Data partialy supports discussion and conclusions: the authors are unable to explain the absence of some sensillas in adults, since they did not assess adult antenna structures.

Response: Thank you very much. Actually, we have performed the studies of adult *D. citri* with SEM and TEM. When we sent our first MS to same professors which have rich experience in SEM and TEM, they also advised us to delete the research on the adult *D. citri* because of the similar research reporting by Onagbola et al. (2008), We agree with the opinion, and keep some adult results in the part of 'Discussion' as supporting information (S1 File and S1 Table,). In adults, eight morphologically distinct types of sensilla were found to distribute on the antennae of female and male *D. citri* in our study (S1 File), with seven types reminiscent of those described by Onagbola et al. (2008). The aporous sensilla trichoidea occurred on the medial portions of the scape, pedicel and flagellomeres 1, 2, 4 and 5, the chaetica sensilla and the unidentified uniporous sensilla which were reported by Onagbola et al. (2008) were absent while the SCA were found in our study (S1 File). In our study, we do not descript the morphology and structure of antennae and antennal sensilla of adult *D. citri*.

(Onagbola EO, Meyer WL, Boina DR, Stelinski LL. Morphological characterization of the antennal sensilla of the Asian citrus psyllid, *Diaphorina citri* Kuwayama (Hemiptera: Psyllidae) with reference to their probable functions. Micron. 2008; 39(8): 1184-1191).

2 I am not sure about the use/choice (parametrical/non-parametrical analyses) because the authors did not mention if data normality was tested, neither number of replicates.

Response: Thank you very much. For the statistical analysis, we consulted an expert (Prof. Wei-Jian Wu from South China Agricultural University who are also major in Biostatistics) and we also added the relevant contents in lines 90-92.

3 There are some mispelling in the text (comments and corrections in the pdf file)

Response: Thank you very much for your comments, we have corrected according to your kind comments, and you can find the one to one response to the pdf file bellow.

4 The species family is wrong. Liviidae instead Psyllidae

Response: Done. Thank you very much.

Responds to Reviewer 3 from the PDF file (PONE-D-19-23769_R1-reviewer):

1 The species was re-classified as Liviidae.

Response: Done. Thank you very much.

2 Line 12, “the most” instead of “an”.

Response: Done. Thank you very much.

3 Please re-phrase the sentence: The literature supports that nymphs were more efficient to transimit Ca. Liberibacter to the host plant. Maybe the fact can be related to feeding behavior aspects. However, nymphs presents capacity to move. On the other hands, adults can move from host plant to another one, transmiting the bacterium to health plants. Other aspect is related to chemical behavior. Initially, adults prefers Ca. Liberibacter plants (high MeSa emittion) and can acquire the bacterium, however, after some days, the adults move to health plants (better nutritional qualities). So, I am not sure that nymphs show a better ability to transmit when compared to adults.

Response: Done. Thank you very much. As reviewers’ comments and suggestions, it is inappropriate to justify the choice for immature stages. We have deleted the sentences, and added some discussion about these issues in the “Discussion” section.

4 I am not sure about the relation of the ‘ability’ of nymphs to transmit the bacterium (feed aspects) with the choice of nymphal antenna to perform the experiments.

Since antenna is related with oddorants, and all behavioral studies with ACP assessed adults behavior and role of compounds, why the authors excluded adult antenna? This study is very welcome to complement ACP behavioral studies.

Response: Thank you very much for your kind suggestion, it is very valuable and helpful for the important guiding significance to our future researches. We have changed the sentence according to the comments. Actually, we also finished the study of adult *D. citri*, but they were not written in our MS because of the similar research reporting by Onagbola et al. (2008). So, we decide to present our adult ACP results as supplementary data in S1 File and S1 Table.

(Onagbola EO, Meyer WL, Boina DR, Stelinski LL. Morphological characterization of the antennal sensilla of the Asian citrus psyllid, *Diaphorina citri* Kuwayama (Hemiptera: Psyllidae), with reference to their probable functions. Micron. 2008; 39(8): 1184-1191.)

5 It is not clear for me what you would like to tell about number of antennae segments. There are 3 segments in insect antennae (scape, pedicel and flagellum). Please clarify what you mean with 'structures'.

Response: Thank you very much for your comment. Yes, there are 3 segments in adult insect antennae (scape, pedicel and flagellum) generally. However, in some low-aged development nymphal stages, such as *Aleurodicus dispersus* and *D. citri*, their flagellum has not yet developed. In our study, we observed two segments of the antennae in first-, second- and third-instar of *D. citri* nymphs. In other words, their flagellum doesn't develop in these ACP nymphs.

6 How many SBs for the other instar? Please clarify? Same for CvS2 and SCA. Please, you need to be clear about the number of sensilla (and sensilla types) that differ among the instars.

Response: Thank you very much for your suggestion. The details of SBs, CvSs and SCA are elaborated in the “Result” section, the number, abundance and distribution of sensilla are listed in Table 2. We don’t list the detailed information in the “Abstract” section, maybe the summarizing information is ok.

7 Lines 29-31: Are you sure that antennae sensilla is related with feeding behavior and ability to carry and transmit Ca Liberibacter? Or is related with volatile perception? Maybe feeding behavior and bacteria acquisition and transmission is related with feeding behavior, interaction with gut and other microorganisms, please justify the relation.

Response: We are very sorry for putting forward the argumentative and uncertain conclusion. Thank you very much for your comments, and we have revised the expression.

8 Line 35, add “also”.

Response: Done. Thank you very much.

9 Use the complete name (*Diaphorina citri*) when start a phase.

Response: Done. Thank you very much.

10 Line 43: associated with (note: there is no Koch’s postulate for these bacteria complex).

Response: Done. Thank you very much.

11 Lines 47-48: Please, there is a controversy for this subject. Please clarify.

Response: Done. Thank you very much for your comment. We have deleted the sentence.

12 Lines 49-50: You should link gustatory function of antennae with feeding behavior

and infective insects in order to justify your theory. I am not convict about the relationship of antennae sensilla and HLB transmission. And why you choose nymphs instead adults (or both), since adults are responsible for.

Response: Thank you very much for your comment. We agree with you, there isn't an exactly relationship of antennae sensilla and HLB transmission. Actually, we also finished the study of adult *D. citri*, but they were not written in our MS because of the similar research reporting by Onagbola et al. (2008).

(Onagbola EO, Meyer WL, Boina DR, Stelinski LL. Morphological characterization of the antennal sensilla of the Asian citrus psyllid, *Diaphorina citri* Kuwayama (Hemiptera: Psyllidae), with reference to their probable functions. Micron. 2008; 39(8): 1184-1191.)

13 Line 66: Perfect. But I am not convicted about the explanation used to justify the nymphs studies. The absence of studies is ok, but I do not agree with ability of transmission vs feeding behavior vs antenna sensilla in nymphs. The introduction needs to be completely written in order to justify the above comment.

Response: Thank you very much, and we have revised the relevant content according to the comment.

14 Line 80: What is the rearing procedure adopted (cite authors). How many generations under laboratory conditions before the experiments? Please clarify the criteria adopted for instar characterization (figure of each instar) and morphological aspects (body length and width, wing buds aspects…days after egg hatch for each nymphal stage…

Response: Thank you very much for your comment. We are very sorry that we don't record the ACP generations after rearing under laboratory conditions before the experiments. Rearing under laboratory conditions, it usually takes about 15-20 days for ACP to finish one generation. So, it may be 14-18 generations when we begin to take the experiments in March 2017.

We refer Ruan et al. (2012) to clarify criteria of ACP instar characterization. Besides the difference of body size between different stages of nymphs, the most important instar characterization is the difference of wing buds. There is no wing buds in the first-instar ACP nymphs. The wing buds are found on the second segment of the thorax in the second-instar ACP nymphs. The wing buds in the third-instar nymphs increase significantly. Their leading edges extend to the posterior edges of their compound eyes, while their posterior edges extend to the 3rd segment of the abdomen. Moreover, their antennal tips turn black and appear caudate beard. In fourth-instar ACP nymphs, the leading edges of wing buds exceed their compound eyes, the posterior edges exceed the 3rd segment of the abdomen. The fifth-instar ACP nymphs are significantly bigger than the other stage nymphs. Their antennae are black except the scape, and the TH1 and TH2 can be clearly observed.

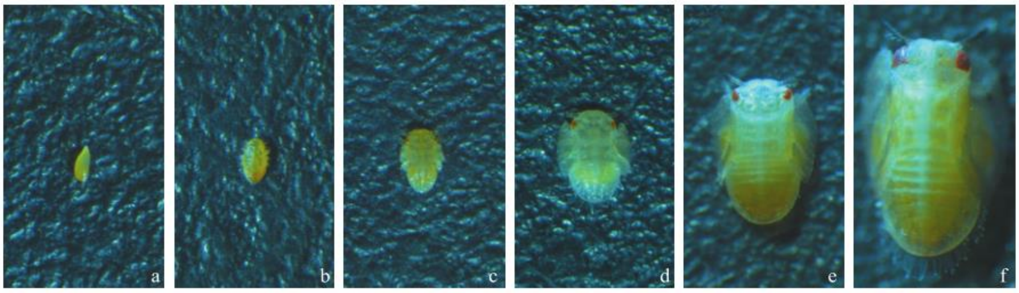

Figure 1 The egg and five different nymphal stages of *Diaphorina citri* under 50 × magnification. (a) Egg; (b) First-instar nymphs; (c) Second-instar nymphs; (d) Third-instar nymphs; (e) Fourth-instar nymphs; (f) Fifth-instar nymphs.

(Ruan CQ, Chen JL, Liu B, Duan YP, Xia YL. 2012. Morphology and behavior of Asian citrus psyllid, *Diaphorina citri* Kuwayama. Chinese Agricultural Science Bulletin, 28(31): 186-190.)

15 number of replications.

Response: Done. Thank you very much.

16 Why parametrical and non-parametrical testes were adopted for different parameters? Please clarify the criteria (data normality…).

Response: Thank you very much. For the statistical analysis, we consulted an expert Prof. Wei-Jian Wu from South China Agricultural University who are also major in Biostatistics. The lengths and widths are conformed to normal distribution, the non-parametrical testes is ok.

17 Line 105: Seems obvious: a pair of antennae is a characteristic of Insecta. This sentence is not helpful.

Response: Thank you very much. We have revised the sentence, you can find in lines 99-100.

18 Lines 221-230: the authors replicated information presented in topic Results.

Response: Thank you very much for your comment. The first paragraph of "Discussion" section, it is the generalization and summarization of the whole results in our study. It may be some replicated information presented in "Results" section.

19 Line 231: This information could be clarified if authors decided to include adults? SB and CvS have degenerated our Onagbola did not see these structures? Can you support the degeneration theory?

Response: Thank you very much for your comment. We found SBs and CvSs in ACP nymphs but not in adults; they were not reported by Onagbola et al. (2008) also. We have added the general analysis of adult antennae and antennal sensilla in S1 File.

20 Line 232: same comment. The authors are assuming the fact, but the fact is not proved. I suggest the addition of study with adults in order to conclude the degeneration/replacement by antennal rhinaria.

Response: Thank you very much for your comment. We add the main results of adult

ACP in S1 File.

21 Lines 235-237: Provide (Order: Family) for the listed species.

Response: Done. Thank you very much.

22 Line 289: Point 1: However, there is an interaction between the bacteria, nutritional medium (presented in insect gut) and the gut that can explain the ability of third nymphs to adults to carry and transimit the bacteria. Point 2: there is a minimum period (feeding period for the nymph to acquire the bacteria. Point 3: there is a latent period of the bacteria before multiplication. Point 4: Thus, that is a minimum concentration of Ca Lbieribacter bacterial title to be detected by Qpcr. Point 5: first to second instar nymphs feed lower than higher nymphal stages and adults, ingesting lower concentration of bacteria (+latent period) + minimum concentration of bacterial title is necessary for qPCR detection could explain this phenomena. I am not convicted about the relationship between sensilla number/types with HLB aquistition/transition by older nymphal stages.

Response: Thank you very much for your comments. They are very valuable and helpful for the important guiding significance to improve our MS and to our future researches. We agree with your opinion, many works must be done to convict about the relationship between sensilla number/types with HLB aquistition/transition by older nymphal stages. We have revised the "Introduction" and "Discussion" section for the expression about the deduction combined with the literatures and the reviewers' comments.

23 The figure should be self-explained. Add insect species and stage (instar).

Response: Done. Thank you very much.

24 *Diaphorina citri* instead *D. citri*.

Response: Done. Thank you very much.

25 Line 526: *Diaphorina citri* instead D. Scientific tables: please use only vertical.

Response: Done. Thank you very much. The layout of tables is used according to the requirement in PLoS ONE. The horizontal line can be used in table.

26 Figures: *Diaporina citri*. Inform instar that you used to take the photos.

Response: Done. Thank you very much.

Special thanks to you for your comments and suggestions.

Responds to the "Reviewer general comments" file:

1 **Title:** Asian citrus psyllid family: Liviidae instead Psyllidae

Response: Done, thank you very much.

2 The authors support the study of morphological characteristics of *D. citri* nymphs based on the fact that immature stages have the ability to transmit *Ca.* Liberibcter more efficiently than adults. This argument is used to justify the choice for immature stages. However, we cannot forget that adults can spread bacteria more efficiently due its flight (from plant to plant). Another aspect that supports the HLB spread is the fact that *D. citri* adults, at first, prefers HLB plants (regulated by MeSa Vocs) and after feeding on HLB plants, move the health plants (better nutritional aspects). I am not sure about the author´s sentence to justify the studies only with nymphs.

Response: Thank you very much for your good suggestion, we are extremely grateful to Reviewers for pointing out this problem. As the Reviewer' comments said that the argument used to lead to the thesis is not very reasonable. We have re-written the relevant contents.

3 The entire literature related to ACP/chemical ecology are based on adult ACP behavior challenged by chemicals from plants or from co-specific insects and their VOCs. Some manuscripts explain the role of ACP adult antenna receptors

challenged by plant and/or chemical volatiles. It is not possible to understand the relationship of immature ACP stages with chemicals, since nymphs do not perform choices in response to chemicals, since nymphs do not perform movements. Thus, I still not convicted about the choice for nymphs only. However, the comparison of nymph antenna structures/sensillas with adults could be helpful for other studies and if done, could be more easy to justify the study.

Response: Thank you very much for your comments. We have performed the studies of adult *D. citri* with SEM and TEM, but we do not write in our MS. It may have many repeated contents with the article of Onagbola et al. (2008), if we reported the structures of antennae and antennal sensilla of adult ACP. The ACP has five nymphal stages, early stages are docile and move only when disturbed or overcrowded (Tsai & Liu, 2000), whereas older nymphs and adults are more mobile (Hall et al., 2013).

(Onagbola EO, Meyer WL, Boina DR, Stelinski LL. Morphological characterization of the antennal sensilla of the Asian citrus psyllid, *Diaphorina citri* Kuwayama (Hemiptera: Psyllidae)

(Tsai JH, Liu YH. 2000. Biology of *Diaphorina citri* (Hemiptera: Psyllidae) on four host plants. Journal of Economic Entomology, 93: 1721-1725.)

(Hall DG, Richardson ML, Ammar ED, Halbert SE. 2013. Asian citrus psyllid, *Diaphorina citri*, vector of citrus huanglongbing disease. Entomologia Experimentalis et Applicata, 146(12): 207-223.)

4 However, the authors cited 2 articles that assessed the antennal structures of adults. However, due to the lack of information (see your discussion topic), the authors are unable to explain the absence of these structures during the investigation of these articles. Maybe there is the absence of these structures in these articles, or the cited authors did not see the mentioned structures. In order to solve this doubt, I encourage the authors to add morphological aspects of adult antenna, since the literature was not helpful to support the findings with nymphs and comparison with

adults.

Response: Thank you very much for your suggestions. We have performed the studies of adult *D. citri* with SEM and TEM, and add part of results as the supplementary data (see S1 File and S1 Table).

5 In general, the introduction is not justified neither well presented. I suggest that this topic could be re-written and that in M&M and Results could be added adult antennal structures.

Response: Thank you very much, and we have re-written this part according to your suggestion. For the adult antennal structures, Onagbola et al. (2008) had reported the research by SEM and TEM, so we think it does not need to write the similar results, just as the supplementary data in S1 File and S1 Table.

(Onagbola EO, Meyer WL, Boina DR, Stelinski LL. Morphological characterization of the antennal sensilla of the Asian citrus psyllid, *Diaphorina citri* Kuwayama (Hemiptera: Psyllidae)

6 There is a lack of substantial information that allow the replication of the protocols. There is no enough information related to the insect (rearing protocol, age, description of what the authors considered as nymph 1, nymph 2, nymph 3, nymph 4 and nymph 5 (see pdf manuscript with my notes).

Response: We are very sorry for our negligence of lacking the important information. We have added the relevant contents in lines 90-92. The classification characteristics of different stages of ACP nymphs are according to Ruan et al. (2012).

(Ruan CQ, Chen JL, Liu B, Duan YP, Xia YL. 2012. Morphology and behavior of Asian citrus psyllid, *Diaphorina citri* Kuwayama. Chinese Agricultural Science Bulletin, 28(31): 186-190).

7 Number of replicates.

Response: Done, thank you very much, and we have added the relevant content in lines 90-92.

8 More details about statistical analyses (see pdf manuscript with my notes).

Response: Done, thank you very much for your comments.

9 The beginning of discussion presents text that is related to results (see pdf manuscript with my notes). The authors are not convicted if antennal structures present in nymphs were degenerated or Onagbola et al did not see this structure in adults (but could be present). This fact did not allow the authors to be convincing during the discussion of the topic. A solution could be the assessment of adult antennal morphological aspects in order to be clear and consistent/convicted to explain the 'possible degeneration' of these structures in ACP adults.

Response: For the adult and nymphal ACP antennal structures, we have performed at least five times with SEM, three times with TEM. The results were the same, and we added some main adult results as the supplementary data in S1 File and S1 Table.

10 Figures should be self-explained (comments in pdf manuscript)

Response: Done, thank you very much.

We tried our best to improve the manuscript and made some changes in the manuscript. We appreciate for Editors/Reviewers' warm work earnestly, and hope that the correction will meet with approval.

Once again, thank you very much for your comments and suggestions.
