# Supplementary material for: Morphological characterization of antennae and antennal sensilla of Diaphorina citri Kuwayama (Hemiptera: Liviidae) nymphs
Source: PLoS One. 2020 Jun 3;15(6):e0234030. doi: 10.1371/journal.pone.0234030 (PMC7269239; doi:10.1371/journal.pone.0234030)
Supplement: Attachment — Submitted filename: Revision 3.pdf [file pone.0234030.s014.pdf]

Revision 3 - -Morphological characterization of antennae and antennal sensilla of *Diaphorina citri* Kuwayama (Hemiptera: Liviidae) nymphs

After a careful review, I consider that the manuscript was improved and deserves to be published in Plos One. However, some “minor appointments” should be addressed before publication (see below).

Page 2 (line 53): Coutinho-Abreu instead Coutnho-Abreu

Page 2 (line 55): Zanardi instead Zanadi

Page 3 (Line 62): add “and” after [21],

Page 4 (line 92): means instead Means

Page 4 (line 105): I think that you refer to Fig 1D and 1E instead Fig1C and 1D – please check

Page 5 (lines 129-138): You should provide information related to terminal hairs width. This information was presented in table, but not in text.

Page 5 (lines 134-135): “The lengths of TH1 and TH2 in the second-instar nymphs were the shortest”: However, your mean comparison don´t say that: For TH1: only nymph2 and nymph 5 are significantly different. For TH2: nymph5 is higher than nymph2 and 3. (Please re-phrase the results based on the statistics, not based in numerical information). When writing width terminal hair, please do the same.

Pages 5 and 6 (Lines 141 – 145): You did not mention Fig 3B. Is this figure relevant? (You need to add in the text or delete the figure 3B).

Page 6 (line 150): Fig 4 (Are you referring to Fig 4A)? Because You did not mention this figure in results topic. Add Fig 4A in line 150 or delete Fig 4A.

Page 6 (line 153): Add Table 2 after “respectively”.

Page 18 (line 498): Table 1 caption: Add “and growth” after “Length”.

Page 20 (Line 528): Table 3 caption: Terminal hairs (TH1 abd TH2) instead “TH1 and TH2”

Fig 3: Cite fig 3B in the text or delete it.
