# Supplementary material for: Morphological characterization of antennae and antennal sensilla of Diaphorina citri Kuwayama (Hemiptera: Liviidae) nymphs
Source: PLoS One. 2020 Jun 3;15(6):e0234030. doi: 10.1371/journal.pone.0234030 (PMC7269239; doi:10.1371/journal.pone.0234030)
Supplement: Attachment — Submitted filename: Respone to Reviewers.docx [file pone.0234030.s005.docx]

We have studied the comments carefully and have made correction which we hope meet with approval. The main corrections in the paper and the responds to the comments are as flowing:

Responds:

1 Change into black all red letters left in the m/s.

Response: Thank you very much. We have done and checked several times.

2 send the m/s for a second cycle of editorial processing; there are many errors, such as DiscuRsion, flower shape vs. flower shaped, The PSO formed with an opened..., the first segment of the antennae was absent sensilla, citrus psylla (the official common name for *D. citri* is Asian Citrus Psyllid (ACP), Acknowledge, to cite a few (please have a thorough review).

Response: We are grateful to Professor Walter S. Leal for the nice and good comments. We are very sorry for the errors which should not be made. So, we have corrected the errors not only listed by Professor Walter S. Leal, but also revised some other errors. Revised portion can be found in the file “Revised Manuscript with Track Changes” easily.

3 the literature is outdated; to a minimum you have to cite the work of Illiano Coutinho when referring to D. citri antennal structure; our two papers on pheromones (please perform an in-depth literature search and update your references).

Response: Thank you very much for the good suggestions. In fact, we have read these two references (“Zanardi OZ, Volpe HXL, Favaris AP, Silva WD, Luvizotto RAG, Magnani RF, et al. Putative sex pheromone of the Asian citrus psyllid, *Diaphorina citri*, breaks down into an attranctant. Scientific Reports. 2018; 8(1): 455.”; “Laboratory and field evaluation of acetic acid-based lures for male Asian citrus psyllid, *Diaphorina citri*. Scientific Reports. 2019; 9(1): 1-10.”), but we do not know how to refer. Our teams also work for the sex pheromone of the ACP. You can find these new references in the page 2 lines55-59.

4 Follow PLoS ONE instructions (Tables go in the Main Text).

Response: Done. Thank you!

5 Improve figure legends; some of them just have a title.

Response: Done. Thank you!

6 Bring relevant TEM figures to the main text.

Response: Thank you very much for the suggestion. However, the TEM figures of the five different nymphal stages are very poor. We do think they can meet the requirements of PLoS One.

We tried our best to improve the manuscript and made some changes in the manuscript. We appreciate for warm work of Professor Walter S. Leal earnestly, and hope that the correction will meet with approval.

Once again, thank you very much for your comments and suggestions.
