# Supplementary material for: Morphological characterization of antennae and antennal sensilla of Diaphorina citri Kuwayama (Hemiptera: Liviidae) nymphs
Source: PLoS One. 2020 Jun 3;15(6):e0234030. doi: 10.1371/journal.pone.0234030 (PMC7269239; doi:10.1371/journal.pone.0234030)
Supplement: Attachment — Submitted filename: review.docx [file pone.0234030.s006.docx]

Zheng et al observed the antennae of the *Diaphorina citri* nymph, and identified the sensilla on the antennae with scanning electron microscopy. The general morphology difference was clarified in different nymph stages. The number, distribution, ultrastructure of the sensilla (11 types) on the nymph antennae was recorded. The function of these sensilla was deduced by comparing with the sensilla of other insect species to explore their role of in Candidatus Liberibacter transmitting and *D. citri* nymph feeding. The results is very interesting. However, this manuscript need some improvements to publish, especially the quality of the photo. It is difficult to get useful information without clear photo showing the characteristic of the sensilla.

1 in the method section of page 3 (line 82-83), why the author used the double-sided adhesive tape, but not a carbon conductive tape. In line 84, the author said they used a XL30, and Nova Nano 430 scanning electron microscopy at 10 kV. In figure 5,B, the photo labeled with 25kV.
2 in line 94, page 4, the length and width of various stages of long terminal hair and short terminal hair were determined. How they measured the length and the width. How many insects they measured. How to minimize the measurement differences due to the viewing angles.
3 I suggest to mark the segments of the antennae in figure 1. For example, it is difficult to find any sensillum on the second- instar nymph without any mark in figure 1A (line 115)and 1B (line 122).
4 All the photos are blurry (especially the photo taken by Nova Nano 430) and it is recommended to shoot again. A good result maybe obtain by Nova Nano 430 at low voltage (for example 5Kv) without dehydrating and drying of the insect.
5 What is the difference between terminal hair and TH1 and TH2 (in paragraph 2, page 6). How to determine the TH1 is longer than TH2,rather than the visual illusion caused by view angle.
6 What is the last ill-defined segment and partitioned sensory organ? (line 171,page 7). A concise legend is needed for figure 4 to help it stand alone ( useful information can be found here, https://blog.bioturing.com/2018/05/10/how-to-craft-a-figure-legend-for-scientific-papers/)
7 What is the cavity sensillum, where it come from?
8 In the discussion section, paragraph 2, the author claimed that another name of TH is chaetica sensilla, why?
9 The reference format should be checked carefully, and keep them in consistent. 2,3, 5,17, 18,,24,37,39,41 44,
