# Supplementary material for: Morphological characterization of antennae and antennal sensilla of Diaphorina citri Kuwayama (Hemiptera: Liviidae) nymphs
Source: PLoS One. 2020 Jun 3;15(6):e0234030. doi: 10.1371/journal.pone.0234030 (PMC7269239; doi:10.1371/journal.pone.0234030)
Supplement: Attachment — Submitted filename: Respone to Reviewers.docx [file pone.0234030.s009.docx]

We have studied the comments carefully and have made correction which we hope meet with approval. The main corrections in the paper and the responds to the comments are as flowing:

**Responds to Reviewer 1 (file “review”):**

Zheng et al observed the antennae of the *Diaphorina citri* nymph, and identified the sensilla on the antennae with scanning electron microscopy. The general morphology difference was clarified in different nymph stages. The number, distribution, ultrastructure of the sensilla (11 types) on the nymph antennae was recorded. The function of these sensilla was deduced by comparing with the sensilla of other insect species to explore their role of in Candidatus Liberibacter transmitting and *D. citri* nymph feeding. The results is very interesting. However, this manuscript need some improvements to publish, especially the quality of the photo. It is difficult to get useful information without clear photo showing the characteristic of the sensilla.

1 in the method section of page 3 (line 82-83), why the author used the double-sided adhesive tape, but not a carbon conductive tape. In line 84, the author said they used a XL30, and Nova Nano 430 scanning electron microscopy at 10 kV. In figure 5,B, the photo labeled with 25kV.

**Response:** Thank you very much for your carefulness. We have performed the studies of SEM both at South China Agriculture University and Centre Laboratory of the South China Botanical Garden several times, but the treatment method and process of the specimens are the same. Most of the SEM photos were got at South China Agriculture University using an SEM (XL30, FEI, Holland and Nova Nano 430, FEI, Holland) at 10kV, few SEM photos were got at Centre Laboratory of the South China Botanical Garden using an SEM (JSM-6360LV, Japan) at 25kV. The reason for the specimens were used the double-sibed adhesive tape, actually, we don’t know why. The method of anchoring the specimens was provided by the researchers in these two electron microscope laboratories.

2 in line 94, page 4, the length and width of various stages of long terminal hair and short terminal hair were determined. How they measured the length and the width. How many insects they measured. How to minimize the measurement differences due to the viewing angles.

**Response:** Thank you very much for your comments. The length and width (basal diameter) of the long terminal hair and short terminal hair are measured from the printed SEM images by a slide caliper. The starting and ending points of the measured data are all in the middle. Ten insects of each stage of *D. citri* were examined.

3 I suggest to mark the segments of the antennae in figure 1. For example, it is difficult to find any sensillum on the second- instar nymph without any mark in figure 1A (line 115) and 1B (line 122).

**Response:** Thank you very much for your suggestion. The first and second segments of the antennae in five different nymphal stages are segmented clearly, and we have marked for the fourth-instar in Fig 1D. In the first- and second-instar nymphs, the total number of antennal sensilla is very small; moreover, they were distributed either on the ventral side or on the dorsal of the antennae. So, we cannot observe all of them in one picture.

4 All the photos are blurry (especially the photo taken by Nova Nano 430) and it is recommended to shoot again. A good result maybe obtain by Nova Nano 430 at low voltage (for example 5Kv) without dehydrating and drying of the insect.

**Response:** Thank you very much for your suggestion. In order to get the high quality photos, we performed our SEM study both at South China Agriculture University and Centre Laboratory of the South China Botanical Garden several times. We even tried to take the specimens without any treatments to anchor for examining by SEM. Here, we hope our photos may meet the requirement of PLoS One.

5 What is the difference between terminal hair and TH1 and TH2 (in paragraph 2, page 6). How to determine the TH1 is longer than TH2, rather than the visual illusion caused by view angle.

**Response:** Thank you very much for your comments. TH1 was distributed with a ST on the antennal tip of different instars of *D. citri* (see Fig 3), including adult females and males (see Fig 1 and Fig 2A in S1 File). In *D. citri* nymphs, we don’t find the morphological difference between TH1 and TH2 except the length and basal diameter (width). However, there is the obvious difference found in adults (see Fig 3 in S1 File). Moreover, the length of TH1 and TH2 was significant difference in the length and width (see Table 2 in S1 File).

6 What is the last ill-defined segment and partitioned sensory organ? (line 171,page 7). A concise legend is needed for figure 4 to help it stand alone ( useful information can be found here, https://blog.bioturing.com/2018/05/10/how-to-craft-a-figure-legend-for-scientific-papers/)

**Response:** Thank you very much for your comments. Partitioned sensory organ was located on the last segment of the antennae of each ACP nymphal stage. The fourth- and fifth-instar nymphs had an ill-defined flagellum which was also the last segment of the antennae. Our expression is not very accurate, and we have revised it. Also, we have checked all of our figure legends, and thank you very much.

7 What is the cavity sensillum, where it come from?

**Response:** Thank you very much for your comment. Cavity sensillum consisted of a sensory cavity and one (Fig 5B) or two pegs (Fig 5A). I have marked the cavity sensillum with a circle, maybe it can be easy to understand. I am very sorry that I don’t really understand that “where it come from?” All sensilla in our study were distributed on the antennae of five different nymphal stages of *D. citri*.

8 In the discussion section, paragraph 2, the author claimed that another name of TH is chaetica sensilla, why?

**Response:** In most of whiteflies and psyllids such as *Trialeurodes vaporariorum*, *Aleyrodes proletella*, *Bemisia tabaci*, *Dialeurodes citri*, *Aleurodicus disperses*, *Psylla pyricola*, *Trioza apicalis* and *D. citri*, there are one or two TH found on the antennal tip. According to Singleton-Smith et al. (1978) and Mellor and Anderson (1995), they are characterized as “chaetica sensilla”, maybe due to their location and the morphology of that they are obvious strong and long with longitudinal groove surface and inserted into a big cuticular socket. These antennal sensilla should be the first to touch and perceive the external environment because of their location and size.

(Singleton-Smith J, Chang JF, Philogène JR. Morphological differences between nymphal instars and descriptions of the antennal sensory structures of the nymphs and adults of *Psylla pyricola* Foerster (Homoptera: Psyllidae). Can J Zool. 1978; 56(7): 1576-1584.)

(Mellor HE, Anderson M. Antennal sensilla of whiteflies: *Trialeurodes vaporariorum* (Westwood), the glasshouse whitefly, *Aleyrodes proletella* (Linnaeus), the cabbage whitefly, and *Bemisia tabaci* (Gennadius), the tobacco whitefly (Homoptera: Aleyrodidae). Part 1: external morphology. International Journal of Insect Morphology and Embryology. 1995; 24(2): 133-143.)

9 The reference format should be checked carefully, and keep them in consistent. 2,3, 5,17, 18,,24,37,39,41 44,

**Response:** Done, thank you very much.

Special thanks to you for your comments and suggestions.

**Responds to Reviewer 2 (from the decision letter):**

1 The manuscript suffers from the lack of an appropriate structural origination. Specifically, in the results section, while the first part focuses mainly on the nymphal instars the authors also deal solely with the presence (or absence) and distribution of various types of the sensilla without first presenting their characteristics. This definitely confuses the readers. I’d suggest the authors rewrite the results section by combining the second part of the results i.e. “Morphology and Structure of Sensilla” with the first part so that in each paragraph the description of each sensilla type should be followed by its presence (or absence), number, distribution, etc., in each nymphal stage. The authors are also encouraged to better formulate their hypothesis in the introduction (the information is there but is not very well promoted!).

**Response:** Thank you very much for your suggestion. We tried to revise our MS according to your suggestion, but we found a little messy after adjusting the part of ‘Result’ of MS layout. We have revised our MS carefully according to the comments, changed the argument used to justify the choice for nymphal stages of *D. citri*, and added some description to improve the structure and quality of our MS, such as lines 147-153.

2 Lack of TEM and/or electrophysiological studies: Usually studies of this kind are accompanied by TEM and/or electrophysiological experiments for strengthening the results. I understand that due to, e.g. lack of equipment or budget constraints, it might not be feasible to conduct the above-mentioned experiments. Hence, I’d suggest that …

**Response:** Thank you very much for your good suggestion, we also try to get some high quality TEM photographs. From March 2017 to September 2018, we have performed the studies of SEM and TEM at South China Agriculture University and Centre Laboratory of the South China Botanical Garden, moreover, at Institute of Food Science and Technology CAAS for TEM. Unfortunately, we did not get good TEM photographs of different nymphal stages of *Diaphorina citri*. For the electrophysiological studies of *D. citri* nymphs, we have not plan to do, but we have done the adult electrophysiological studies. ACP is a small insect, and it is not easy to connect the antennae in the adult electrophysiological studies. Moreover, the various nymphal stages of *D. citri* antennae are much shorter than the adults (see Table 1 in S1 File), especially the first-, second-, third- and fourth-instar nymphal antennae (Table 1). Thank you very much for your suggestion, it is very helpful for our future researches.

3 … the authors submit their findings to other journals (such as Micron) that publish these kinds of works so as to attract more readers interested in the SEM.

**Response:** Thank you very much for your suggestion, most of our teams’ works about the SEM or TEM have published in Micron, Microscopy Research and Technique, as well as Plos One. ACP is the most important insect, and there are also quite a few researches about the ACP published in Plos One. We also think it is appropriate to submit our MS to Plos One.

4 I believe the following items will increase the value of the manuscript: 1) Inclusion of parameters like the size of individual flagellomeres across the nymphal instars. 2) Figuring out if there is a correlation between the nymph size within each nymphal stage and the number of sensilla? 3) Were the individuals size-matched? I’d suggest the authors include the average head-width (indicative of size of the nymphs) of each nymphal stage in table 1.

**Response:** 1) Thank you very much for your suggestions. It is very valuable and helpful for the important guiding significance to our future researches. 2) The length of the five different nymphal antennae was significantly increased with the increase of the nymphal instars, as well as the number of sensilla. We do not find the correlation between the nymph size within each nymphal stage and the number of sensilla. 3) Yes, it is size-matched. The classification characteristics of different stages of ACP nymphs are according to Ruan et al. (2012). The SEM photographs were just only antennae, not containing the whole head, so, it could not measure the ACP head-width of each nymphal stage. Thank you very much for your suggestions.

(Ruan CQ, Chen JL, Liu B, Duan YP, Xia YL. 2012. Morphology and behavior of Asian citrus psyllid, *Diaphorina citri* Kuwayama. Chinese Agricultural Science Bulletin, 28(31): 186-190)

5 Lines 14-15: “Because its ability to transmit C. Liberibacter was more efficient in nymphs” please change it to “Because the nymphal instars have been reported to more efficiently transmit C. Liberibacter”.

**Response:** Done, thank you very much.

6 Line 25: “The distribution of antennal sensilla in each nymphal stage of *D. citri* was asymmetrical”. Do you mean they are “randomly distributed”?

**Response:** No, it just means that the distribution of antennal sensilla in nymphs was not the same. The SBs, CvS2 and SCA are the examples of how the antennal sensilla in nymphs asymmetrically distribute. These sensilla are not distributed in each nymphal stage, only in the older nymphs such as third-, fourth- and fifth-instar.

7 Lines 93-97: Under statistical analysis, instead of clearly explaining how they analyzed their data, the authors refer their readers to 11 references that focus on the classification of the sensilla. Please remove the author names and instead write, “…similar structures described previously [27-35].

**Response:** Done, thank you very much.

8 When the antennae are mounted only one side of the antennae is facing upward. It is not clear how the number of different sensilla were counted. Please elaborate on that.

**Response:** Done, thank you very much. Each side of the antennae (dorsal, ventral, and two lateral surfaces of different nymphal stages of *D. citri*) was mounted to obverse. Each measured parameters was ten, and we have added the relevant contents in lines 90-92.

9 Figure legends must be self-explanatory. For example in figure 1 it must be clearly explained that A-C belong to the 1st through the 3rd nymphal instars with two antennal flagellomeres and D-E belongs to the 4th and 5th nymphal with three flagellomeres.

**Response:** Done, thank you very much.

10 Lines 139-140: basal segments and subsegments must be indicated in the figure.

**Response:** Done, thank you very much.

11 “Se 1” or “S1”? Please be consistent.

**Response:** I am very sorry for using such similar abbreviation. Actually, “Se1” is the abbreviation of the first segment of antennae, while “S1” is the abbreviation of the “S1 Fig.” in the file “Supplementary data”. We have renamed the supplementary.

12 Line 244: please change “trichoids hairs” to “trichoid sensilla”

**Response:** Done, thank you very much.

Special thanks to you for your comments and suggestions.

**Responds to Reviewer 3 (from the decision letter):**

1 Data partialy supports discussion and conclusions: the authors are unable to explain the absence of some sensillas in adults, since they did not assess adult antenna structures.

**Response:** Thank you very much. Actually, we have performed the studies of adult *D. citri* with SEM and TEM. When we sent our first MS to same professors which have rich experience in SEM and TEM, they also advised us to delete the research on the adult *D. citri* because of the similar research reporting by Onagbola et al. (2008), We agree with the opinion, and keep some adult results in the part of ‘Discussion’ as supporting information (S1 File and S1 Table,). In adults, eight morphologically distinct types of sensilla were found to distribute on the antennae of female and male *D. citri* in our study (S1 File), with seven types reminiscent of those described by Onagbola et al. (2008). The aporous sensilla trichoidea occurred on the medial portions of the scape, pedicel and flagellomeres 1, 2, 4 and 5, the chaetica sensilla and the unidentified uniporous sensilla which were reported by Onagbola et al. (2008) were absent while the SCA were found in our study (S1 File). In our study, we do not descript the morphology and structure of antennae and antennal sensilla of adult *D. citri*.

(Onagbola EO, Meyer WL, Boina DR, Stelinski LL. Morphological characterization of the antennal sensilla of the Asian citrus psyllid, *Diaphorina citri* Kuwayama (Hemiptera: Psyllidae) with reference to their probable functions. Micron. 2008; 39(8): 1184-1191).

2 I am not sure about the use/choice (parametrical/non-parametrical analyses) because the authors did not mention if data normality was tested, neither number of replicates.

**Response:** Thank you very much. For the statistical analysis, we consulted an expert (Prof. Wei-Jian Wu from South China Agricultural University who are also major in Biostatistics) and we also added the relevant contents in lines 90-92.

3 There are some mispelling in the text (comments and corrections in the pdf file)

**Response:** Thank you very much for your comments, we have corrected according to your kind comments, and you can find the one to one response to the pdf file bellow.

4 The species family is wrong. Liviidae instead Psyllidae

**Response:** Done. Thank you very much.

**Responds to Reviewer 3 from the PDF file (PONE-D-19-23769_R1-reviewer):**

1 The species was re-classified as Liviidae.

**Response:** Done. Thank you very much.

2 Line 12, “the most” instead of “an”.

**Response:** Done. Thank you very much.

3 Please re-phrase the sentence: The literature supports that nymphs were more efficient to transimit Ca. Liberibacter to the host plant. Maybe the fact can be related to feeding behavior aspects. However, nymphs presents capacity to move. On the other hands, adults can move from host plant to another one, transmiting the bacterium to health plants. Other aspect is related to chemical behavior. Initially, adults prefers Ca. Liberibacter plants (high MeSa emittion) and can acquire the bacterium, however, after some days, the adults move to health plants (better nutritional qualities). So, I am not sure that nymphs show a better ability to transmit when compared to adults.

**Response:** Done. Thank you very much. As reviewers’ comments and suggestions, it is inappropriate to justify the choice for immature stages. We have deleted the sentences, and added some discussion about these issues in the “Discussion” section.

4 I am not sure about the relation of the ‘ability’ of nymphs to transmit the bacterium (feed aspects) with the choice of nymphal antenna to perform the experiments. Since antenna is related with oddorants, and all behavioral studies with ACP assessed adults behavior and role of compounds, why the authors excluded adult antenna? This study is very welcome to complement ACP behavioral studies.

**Response:** Thank you very much for your kind suggestion, it is very valuable and helpful for the important guiding significance to our future researches. We have changed the sentence according to the comments. Actually, we also finished the study of adult *D. citri*, but they were not written in our MS because of the similar research reporting by Onagbola et al. (2008). So, we decide to present our adult ACP results as supplementary data in S1 File and S1 Table.

(Onagbola EO, Meyer WL, Boina DR, Stelinski LL. Morphological characterization of the antennal sensilla of the Asian citrus psyllid, *Diaphorina citri* Kuwayama (Hemiptera: Psyllidae), with reference to their probable functions. Micron. 2008; 39(8): 1184-1191.)

5 It is not clear for me what you would like to tell about number of antennae segments. There are 3 segments in insect antennae (scape, pedicel and flagellum). Please clarify what you mean with ‘structures’.

**Response:** Thank you very much for your comment. Yes, there are 3 segments in adult insect antennae (scape, pedicel and flagellum) generally. However, in some low-aged development nymphal stages, such as *Aleurodicus dispersus* and *D. citri*, their flagellum has not yet developed. In our study, we observed two segments of the antennae in first-, second- and third-instar of *D. citri* nymphs. In other words, their flagellum doesn’t develop in these ACP nymphs.

6 How many SBs for the other instar? Please clarify? Same for CvS2 and SCA. Please, you need to be clear about the number of sensilla (and sensilla types) that differ among the instars.

**Response:** Thank you very much for your suggestion. The details of SBs, CvSs and SCA are elaborated in the “Result” section, the number, abundance and distribution of sensilla are listed in Table 2. We don’t list the detailed information in the “Abstract” section, maybe the summarizing information is ok.

7 Lines 29-31: Are you sure that antennae sensilla is related with feeding behavior and ability to carry and transmit Ca Liberibacter? Or is related with volatile perception? Maybe feeding behavior and bacteria acquisition and transmission is related with feeding behavior, interaction with gut and other microorganisms, please justify the relation.

**Response:** We are very sorry for putting forward the argumentative and uncertain conclusion. Thank you very much for your comments, and we have revised the expression.

8 Line 35, add “also”.

**Response:** Done. Thank you very much.

9 Use the complete name (*Diaphorina citri*) when start a phase.

**Response:** Done. Thank you very much.

10 Line 43: associated with (note: there is no Koch’s postulate for these bacteria complex).

**Response:** Done. Thank you very much.

11 Lines 47-48: Please, there is a controversy for this subject. Please clarify.

**Response:** Done. Thank you very much for your comment. We have deleted the sentence.

12 Lines 49-50: You should link gustatory function of antennae with feeding behavior and infective insects in order to justify your theory. I am not convict about the relationship of antennae sensilla and HLB transmission. And why you choose nymphs instead adults (or both), since adults are responsible for.

**Response:** Thank you very much for your comment. We agree with you, there isn’t an exactly relationship of antennae sensilla and HLB transmission. Actually, we also finished the study of adult *D. citri*, but they were not written in our MS because of the similar research reporting by Onagbola et al. (2008).

(Onagbola EO, Meyer WL, Boina DR, Stelinski LL. Morphological characterization of the antennal sensilla of the Asian citrus psyllid, *Diaphorina citri* Kuwayama (Hemiptera: Psyllidae), with reference to their probable functions. Micron. 2008; 39(8): 1184-1191.)

13 Line 66: Perfect. But I am not convicted about the explanation used to justify the nymphs studies. The absence of studies is ok, but I do not agree with ability of transmission vs feeding behavior vs antenna sensilla in nymphs. The introduction needs to be completely written in order to justify the above comment.

**Response:** Thank you very much, and we have revised the relevant content according to the comment.

14 Line 80: What is the rearing procedure adopted (cite authors). How many generations under laboratory conditions before the experiments? Please clarify the criteria adopted for instar characterization (figure of each instar) and morphological aspects (body length and width, wing buds aspects…days after egg hatch for each nymphal stage…

**Response:** Thank you very much for your comment. We are very sorry that we don’t record the ACP generations after rearing under laboratory conditions before the experiments. Rearing under laboratory conditions, it usually takes about 15-20 days for ACP to finish one generation. So, it may be 14-18 generations when we begin to take the experiments in March 2017. We refer Ruan et al. (2012) to clarify criteria of ACP instar characterization. Besides the difference of body size between different stages of nymphs, the most important instar characterization is the difference of wing buds. There is no wing buds in the first-instar ACP nymphs. The wing buds are found on the second segment of the thorax in the second-instar ACP nymphs. The wing buds in the third-instar nymphs increase significantly. Their leading edges extend to the posterior edges of their compound eyes, while their posterior edges extend to the 3^rd^ segment of the abdomen. Moreover, their antennal tips turn black and appear caudate beard. In fourth-instar ACP nymphs, the leading edges of wing buds exceed their compound eyes, the posterior edges exceed the 3^rd^ segment of the abdomen. The fifth-instar ACP nymphs are significantly bigger than the other stage nymphs. Their antennae are black except the scape, and the TH1 and TH2 can be clearly observed.

**Figure 1 The egg and five different nymphal stages of *Diaphorina citri* under 50 × magnification.** (a) Egg; (b) First-instar nymphs; (c) Second-instar nymphs; (d) Third-instar nymphs; (e) Fourth-instar nymphs; (f) Fifth-instar nymphs.

(Ruan CQ, Chen JL, Liu B, Duan YP, Xia YL. 2012. Morphology and behavior of Asian citrus psyllid, *Diaphorina citri* Kuwayama. Chinese Agricultural Science Bulletin, 28(31): 186-190.)

15 number of replications.

**Response:** Done. Thank you very much.

16 Why parametrical and non-parametrical testes were adopted for different parameters? Please clarify the criteria (data normality…).

**Response:** Thank you very much. For the statistical analysis, we consulted an expert Prof. Wei-Jian Wu from South China Agricultural University who are also major in Biostatistics. The lengths and widths are conformed to normal distribution, the non-parametrical testes is ok.

17 Line 105: Seems obvious: a pair of antennae is a characteristic of Insecta. This sentence is not helpful.

**Response:** Thank you very much. We have revised the sentence, you can find in lines 99-100.

18 Lines 221-230: the authors replicated information presented in topic Results.

**Response:** Thank you very much for your comment. The first paragraph of “Discussion” section, it is the generalization and summarization of the whole results in our study. It may be some replicated information presented in “Results” section.

19 Line 231: This information could be clarified if authors decided to include adults? SB and CvS have degenerated our Onagbola did not see these structures? Can you support the degeneration theory?

**Response:** Thank you very much for your comment. We found SBs and CvSs in ACP nymphs but not in adults; they were not reported by Onagbola et al. (2008) also. We have added the general analysis of adult antennae and antennal sensilla in S1 File.

20 Line 232: same comment. The authors are assuming the fact, but the fact is not proved. I suggest the addition of study with adults in order to conclude the degeneration/replacement by antennal rhinaria.

**Response:** Thank you very much for your comment. We add the main results of adult ACP in S1 File.

21 Lines 235-237: Provide (Order: Family) for the listed species.

**Response:** Done. Thank you very much.

22 Line 289: Point 1: However, there is an interaction between the bacteria, nutritional medium (presented in insect gut) and the gut that can explain the ability of third nymphs to adults to carry and transimit the bacteria. Point 2: there is a minimum period (feeding period for the nymph to acquire the bacteria. Point 3: there is a latent period of the bacteria before multiplication. Point 4: Thus, that is a minimum concentration of Ca Lbieribacter bacterial title to be detected by Qpcr. Point 5: first to second instar nymphs feed lower than higher nymphal stages and adults, ingesting lower concentration of bacteria (+latent period) + minimum concentration of bacterial title is necessary for qPCR detection could explain this phenomena. I am not convicted about the relationship between sensilla number/types with HLB aquistition/transition by older nymphal stages.

**Response:** Thank you very much for your comments. They are very valuable and helpful for the important guiding significance to improve our MS and to our future researches. We agree with your opinion, many works must be done to convict about the relationship between sensilla number/types with HLB aquistition/transition by older nymphal stages. We have revised the “Introduction” and “Discussion” section for the expression about the deduction combined with the literatures and the reviewers’ comments.

23 The figure should be self-explained. Add insect species and stage (instar).

**Response:** Done. Thank you very much.

24 *Diaphorina citri* instead *D. citri*.

**Response:** Done. Thank you very much.

25 Line 526: *Diaphorina citri* instead D. Scientific tables: please use only vertical.

**Response:** Done. Thank you very much. The layout of tables is used according to the requirement in PLoS ONE. The horizontal line can be used in table.

26 Figures: *Diaporina citri*. Inform instar that you used to take the photos.

**Response:** Done. Thank you very much.

Special thanks to you for your comments and suggestions.

**Responds to the “Reviewer general comments” file:**

1 **Title:** Asian citrus psyllid family: Liviidae instead Psyllidae

**Response:** Done, thank you very much.

2 The authors support the study of morphological characteristics of *D. citri* nymphs based on the fact that immature stages have the ability to transmit *Ca.* Liberibcter more efficiently than adults. This argument is used to justify the choice for immature stages. However, we cannot forget that adults can spread bacteria more efficiently due its flight (from plant to plant). Another aspect that supports the HLB spread is the fact that *D. citri* adults, at first, prefers HLB plants (regulated by MeSa Vocs) and after feeding on HLB plants, move the health plants (better nutritional aspects). I am not sure about the author´s sentence to justify the studies only with nymphs.

**Response:** Thank you very much for your good suggestion, we are extremely grateful to Reviewers for pointing out this problem. As the Reviewer’ comments said that the argument used to lead to the thesis is not very reasonable. We have re-written the relevant contents.

3 The entire literature related to ACP/chemical ecology are based on adult ACP behavior challenged by chemicals from plants or from co-specific insects and their VOCs. Some manuscripts explain the role of ACP adult antenna receptors challenged by plant and/or chemical volatiles. It is not possible to understand the relationship of immature ACP stages with chemicals, since nymphs do not perform choices in response to chemicals, since nymphs do not perform movements. Thus, I still not convicted about the choice for nymphs only. However, the comparison of nymph antenna structures/sensillas with adults could be helpful for other studies and if done, could be more easy to justify the study.

**Response:** Thank you very much for your comments. We have performed the studies of adult *D. citri* with SEM and TEM, but we do not write in our MS. It may have many repeated contents with the article of Onagbola et al. (2008), if we reported the structures of antennae and antennal sensilla of adult ACP. The ACP has five nymphal stages, early stages are docile and move only when disturbed or overcrowded (Tsai & Liu, 2000), whereas older nymphs and adults are more mobile (Hall et al., 2013).

(Onagbola EO, Meyer WL, Boina DR, Stelinski LL. Morphological characterization of the antennal sensilla of the Asian citrus psyllid, *Diaphorina citri* Kuwayama (Hemiptera: Psyllidae)

(Tsai JH, Liu YH. 2000. Biology of *Diaphorina citri* (Hemiptera: Psyllidae) on four host plants. Journal of Economic Entomology, 93: 1721-1725.)

(Hall DG, Richardson ML, Ammar ED, Halbert SE. 2013. Asian citrus psyllid, *Diaphorina citri*, vector of citrus huanglongbing disease. Entomologia Experimentalis et Applicata, 146(12): 207-223.)

4 However, the authors cited 2 articles that assessed the antennal structures of adults. However, due to the lack of information (see your discussion topic), the authors are unable to explain the absence of these structures during the investigation of these articles. Maybe there is the absence of these structures in these articles, or the cited authors did not see the mentioned structures. In order to solve this doubt, I encourage the authors to add morphological aspects of adult antenna, since the literature was not helpful to support the findings with nymphs and comparison with adults.

**Response:** Thank you very much for your suggestions. We have performed the studies of adult *D. citri* with SEM and TEM, and add part of results as the supplementary data (see S1 File and S1 Table).

5 In general, the introduction is not justified neither well presented. I suggest that this topic could be re-written and that in M&M and Results could be added adult antennal structures.

**Response:** Thank you very much, and we have re-written this part according to your suggestion. For the adult antennal structures, Onagbola et al. (2008) had reported the research by SEM and TEM, so we think it does not need to write the similar results, just as the supplementary data in S1 File and S1 Table.

(Onagbola EO, Meyer WL, Boina DR, Stelinski LL. Morphological characterization of the antennal sensilla of the Asian citrus psyllid, *Diaphorina citri* Kuwayama (Hemiptera: Psyllidae)

6 There is a lack of substantial information that allow the replication of the protocols. There is no enough information related to the insect (rearing protocol, age, description of what the authors considered as nymph 1, nymph 2, nymph 3, nymph 4 and nymph 5 (see pdf manuscript with my notes).

**Response:** We are very sorry for our negligence of lacking the important information. We have added the relevant contents in lines 90-92. The classification characteristics of different stages of ACP nymphs are according to Ruan et al. (2012).

(Ruan CQ, Chen JL, Liu B, Duan YP, Xia YL. 2012. Morphology and behavior of Asian citrus psyllid, *Diaphorina citri* Kuwayama. Chinese Agricultural Science Bulletin, 28(31): 186-190).

7 Number of replicates.

**Response:** Done, thank you very much, and we have added the relevant content in lines 90-92.

8 More details about statistical analyses (see pdf manuscript with my notes).

**Response:** Done, thank you very much for your comments.

9 The beginning of discussion presents text that is related to results (see pdf manuscript with my notes). The authors are not convicted if antennal structures present in nymphs were degenerated or Onagbola et al did not see this structure in adults (but could be present). This fact did not allow the authors to be convincing during the discussion of the topic. A solution could be the assessment of adult antennal morphological aspects in order to be clear and consistent/convicted to explain the ‘possible degeneration’ of these structures in ACP adults.

**Response:** For the adult and nymphal ACP antennal structures, we have performed at least five times with SEM, three times with TEM. The results were the same, and we added some main adult results as the supplementary data in S1 File and S1 Table.

10 Figures should be self-explained (comments in pdf manuscript)

**Response:** Done, thank you very much.

We tried our best to improve the manuscript and made some changes in the manuscript. We appreciate for Editors/Reviewers’ warm work earnestly, and hope that the correction will meet with approval.

Once again, thank you very much for your comments and suggestions.
