# Supplementary material for: Morphological characterization of antennae and antennal sensilla of Diaphorina citri Kuwayama (Hemiptera: Liviidae) nymphs
Source: PLoS One. 2020 Jun 3;15(6):e0234030. doi: 10.1371/journal.pone.0234030 (PMC7269239; doi:10.1371/journal.pone.0234030)
Supplement: Attachment — Submitted filename: Plos one Review 26-01-2020.docx [file pone.0234030.s010.docx]

The manuscript has been greatly improved, and researcher can benefit from this research on the antennae ultrastructure of ACP. However, there is no more figures to show the pores which are hypothesized where the semiochemicals can enter the antennae. It will be difficult to support the claim that some sensilla are involved (or function)in “olfactory behavior and host identification”. I strong suggested that the authors should add more figures to show the pores on the sensilla surface with SEM or TEM. There is another paper on Micron which showed that they observed the multiparous structure with the same pest <https://www.sciencedirect.com/science/article/pii/S0968432808001182>. Thus I suggest the authors should make a more interesting story to publish their findings.
