# Supplementary material for: Morphological characterization of antennae and antennal sensilla of Diaphorina citri Kuwayama (Hemiptera: Liviidae) nymphs
Source: PLoS One. 2020 Jun 3;15(6):e0234030. doi: 10.1371/journal.pone.0234030 (PMC7269239; doi:10.1371/journal.pone.0234030)
Supplement: Attachment — Submitted filename: Respone to Reviewers.docx [file pone.0234030.s013.docx]

We very much appreciate the careful reading of our MS and valuable suggestions of the reviewers. We have carefully considered the comments and have revised the MS accordingly. The main corrections in the paper and the responds to the comments are as following:

**Responds to Reviewer 1 (from the decision letter)**

1. The manuscript has been greatly improved, and the researcher can benefit from this interesting research on the antennae ultrastructure of ACP. However, there is no more figures to show the pores which are hypothesized where the semiochemicals can enter the antennae. It will be difficult to support the claim that some sensilla are involved (or function) in “olfactory behavior and host identification”. I strongly suggested that the authors should add more figures to show the pores on the sensilla surface with SEM or TEM. There is another paper on Micron which showed that they observed the multiparous structure with the same pest https://www.sciencedirect.com/science/article/pii/S0968432808001182. Thus, I suggest the authors should make a more interesting story to publish their findings.

**Response:** Thank you very much for your comments. We also know that it will well improve the quality of our MS using SEM and TEM together. We have tried our best to get some high quality TEM photographs. Unfortunately, we failed. We performed the TEM studies at least three times at three different electron microscopy centers including Centre Laboratory of the South China Botanical Garden where we successfully got the good quality TEM photographs in 2015. Our teams have done a lot of studies about SEM (Zheng et al., 2014; 2016; Xue et al., 2015; Hang et al., 2018) and TEM (Zheng et al., 2016), but only one time to get the perfect TEM photographs (Zheng et al., 2016). Because of the outbreak of the novel coronavirus in our country, we can’t perform the TEM study within these days. The other reason we don’t want to try it again is because its cost is too expensive to perform these studies in the electron microscopy centers. The costs of SEM and TEM are often more than 10000 CNY (about 1430 $) for one time. The total costs of SEM and TEM (three times together) was close to 35000 CNY (about 5000 $). We are very sorry that we decide not to try it again for this situation. Thank you very much for your kind suggestion, it is very helpful and valuable for our future researches.

Han-Ying Yang^#^, **Li-Xia Zheng^#^**, Zhen-Fei Zhang, Yang Zhang, Wei-Jian Wu. The structure and morphologic changes of antennae of *Cyrtorhinus lividipennis* (Hemiptera: Miridae: Orthotylinae) in different instars. PLoS ONE, 2018,13(11): e0207551.

Xue H, **Zheng LX**, Wu WJ. Morphometry of compound eyes of three *Bactrocera* (Diptera: Tephritidae) species. Florida Entomologist, 2015, 98(2): 806-809.

**Zheng LX**, Wu WJ, Liang GW, Fu YG. Nymphal antennae and antennal sensilla in *Aleurodicus dispersus* (Hemiptera: Aleyrodidae). Bulletin of Entomological Research, 2014, 104: 622-630.

Zheng YH^#^, **Zheng LX^#^**, Liao YL, Wu WJ. Sexual dimorphism in antennal morphology and sensilla ultrastructure of a pupal endoparasitoid *Tetrastichus howardi* (Olliff) (Hymenoptera: Eulophidae). Microscopy Research and Technique, 2016, 79: 374-384.

2. The full journal name should be shown in Reference 3.

**Response:** Done. Thank you very much.

**Responds to Reviewer 1 from the file (Plos one Review 26-01-2020):**

1. The manuscript has been greatly improved, and researcher can benefit from this research on the antennae ultrastructure of ACP. However, there is no more figures to show the pores which are hypothesized where the semiochemicals can enter the antennae. It will be difficult to support the claim that some sensilla are involved (or function) in “olfactory behavior and host identification”. I strong suggested that the authors should add more figures to show the pores on the sensilla surface with SEM or TEM. There is another paper on Micron which showed that they observed the multiparous structure with the same pest <https://www.sciencedirect.com/science/article/pii/S0968432808001182>. Thus I suggest the authors should make a more interesting story to publish their findings.

**Response:** Thank you very much for your suggestion. Yes, this reference is also very valuable and helpful to our MS. Our SEM photographs of adult ACP (see S1 File) are also perfect quality, but we don’t get the good quality TEM photographs both nymphal and adult antennal sensilla. We are very sorry that we could not provide any more perfect photographs of SEM or TEM. Thank you very much again for your suggestion, it is very nice and valuable. Here, we hope our photos may meet the requirement of PLoS One.

Special thanks to you for your comments and suggestions.

**Responds to Reviewer 2 (from the decision letter)**

1. I am not convinced by the authors’ justification for not wanting to revise the results section. As it stands, the very first thing that the readers come across in the results section is the name (or some abbreviations) of sensilla (TH1, TH2, ST, etc). Instead, readers should first be introduced to these sensilla and what their characteristics are FOLLOWED BY information regarding their presence/absence.

**Response:** Done. Thank you very much for your comments again. Yes, as you see, it is much concise in the results section after revising according to your suggestion.

2. As I mentioned before, I am fully aware of the constraints the authors might have to performing TEM and or electrophysiology and that was why I suggested it to be submitted to, e.g., Micron as it publishes findings solely on SEM. The previous works the authors referenced, such as the one published in Micron, have a combination of SEM and TEM. Likewise, the works published in other journals include molecular work or amino acid alignment and not only SEM.

**Response:** Thank you very much for your suggestions again. We also agree with you. It will be really well to improve our MS adding TEM and electrophysiology studies. The reason of why we don’t add the TEM study is stated above. Also, we could not add the nymphal electrophysiological study due to the difficulty in connecting the nymphal antennae. We are very sorry, but your suggestions and comments are very valuable and helpful for our future researches.

Special thanks to you for your comments and suggestions.

**Responds to Reviewer 3 (from the decision letter)**

1. The manuscript entitled “Morphological characterization of nymphal antennae and antennal sensilla of *Diaphorina citri* Kuwayama (Hemiptera: Liviidae)’ reports general aspects of antenna/sensilla morphology of *Diaphorina citri*. The revision 2 was significantly improved, however major points need to be carefully corrected before its publication in Plos One.
In my opinion, a final revision round is desirable in order to be sure that the paper is clear to be published.

**Response:** Thank you very much for your kind comments and suggestions. We learned it carefully and done one to one response to the comments.

**Responds to Reviewer 3 (file “PONE-D-23769_R2”):**

1. Line 43: It is plural, since is associated to 3 bacteria.

**Response:** Done. Thank you very much.

2. Line 111: these sensilla refers to table 2? Cite it to clarify information.

**Response:** Thank you very much. According to the other reviewer’s suggestion, we rewrote part of the results and deleted the sentence.

3. Line 117: 35.96 longer: These information was not presented in Table 1. Suggestion: Include a new column in this table adding the antenna growth (+SE) for each instar (2-5 instar) (instar 2 size - instar 1 size = ?)

**Response:** Done. Thank you very much for your kind suggestion.

4. Line 119: I found this information in Table 2. Please cite it.

**Response:** Done. Thank you very much. According to the other reviewer’s suggestion, we rewrote part of the results and deleted the sentence.

5. Line 125: 1.5 times longer. Suggestion: Standardization, xxx um longer (as expressed for topic (second instar). Use the information that you will provide in the new column (Table 1) (instar 3 size - instar 2 size = ?) antenna growth. Dont forget to include SE in table and text.

**Response:** Done. Thank you very much.

6. Line 127: Are you referring to Table 2? Cite it here in order to clarify the information.

**Response:** Done. Thank you very much. According to the other reviewer’s suggestion, we rewrote part of the results and deleted the sentence.

7. Line 132: Information missed: The authors presents the antenna growth for instar 2 and 3. But what is the growth in 4 instar (4 instar size - 3 instar size = growth)

**Response:** Done. Thank you very much.

8. Line 139: For standardization: add antenna growth in this topic. (instar 5 - instar 4 = ?

**Response:** Done. Thank you very much.

9. Line 141: For standardization: add antenna growth in this topic. (instar 5- instar 4 = ?).

**Response:** Done. Thank you very much for the reviews of 2-9, we have rewritten this part of results according to the other reviewer’s suggestion. Also, we have added some information according to your suggestions of 2-9.

10. Line 140: Please check your Figure. I can’t find Fig 1 F. Figure legend is wrong (A-C, ok) (D-H, wrong). I think (D-E).

**Response:** Yes, you are right. Thank you very much.

11. Line 156: Fig 2 A refers to a 3rd instar nymph, but you assume in the sentence that is related to 5th instar. In this way, I added a text as a solution to solve this issue.

**Response:** Done. Thank you very much for your kind suggestion.

12. Line 167: Check my suggestion, since: Table 2 supports that you found ST for all nymphal stages! And Fig 3 is related only for a third instar nymph.

**Response:** Thank you very much for your kind suggestion, but I think that Table 2 just shows the abundance and distribution of ST, here we showing morphology of ST in this sentence, Table 2 may be cited in the last sentence. What do you think?

13. Line 174: Same issue here: Table 2 indicates that you found PSO for all instars and Fig 4 refers to fifth instar nymph.

**Response:** Thank you very much for your suggestions. The reviews of 10-13 may be the same issue. It is not so good to present all photographs of all types of antennal sensilla of each nymphal stage of *D. citri*. For the same type of antennal sensilla, one of the stages of nymphal *D. citri* was chosen to perform the morphology and structure in figure, even if they can be found in each nymphal stage. The SEM quality of antennal sensilla in the first- and second-instar nymphs is a little worse than the others, so they were not presented in figures. The photographs we chose should be the good quality, and we think it is best to include each nymphal stage of *D. citri*.

14. Lines 226-231: Highlighted in yellow and red: Results replicated.

**Response:** Thank you very much, and we have deleted.

15. Line 286: First citation: add (Order: Family).

**Response:** Thank you very much. It has been cited in lines 240-241 in Reference 32, not first citation.

16. Table 1: Add a column Growth um and add analyses.

**Response:** Done. Thank you very much for your kind suggestion, it is also valuable and helpful for our MS and other researches.

17. Tables: use only horizontal lines…Since it is a scientific table.

**Response:** Thank you very much for your suggestion. I agree with you, but it is the format requirement of the table in PLoS ONE. In addition, we unload the usual scientific table in the file of “Tables”. If they need, they can also choose to use.

18. Table 2: format this colmn. (SB3).

**Response:** Done. Thank you very much.

**Responds to Reviewer 3 (file “Reviewer comments R2”):**

**Response:** Thank you very much for your kind and valuable suggestions. I read the comments of file “Reviewer comments R2” carefully and find that they are the replicated suggestions and comments with the file “PONE-D-23769_R2”. So, you can find the responds above. Special thanks to you for your comments and suggestions.

We tried our best to improve the manuscript and made some changes in the manuscript. We appreciate for Editors/Reviewers’ warm work earnestly, and hope that the correction will meet with approval.

Once again, thank you very much for your comments and suggestions.
