# Supplementary material for: Morphological characterization of antennae and antennal sensilla of Diaphorina citri Kuwayama (Hemiptera: Liviidae) nymphs
Source: PLoS One. 2020 Jun 3;15(6):e0234030. doi: 10.1371/journal.pone.0234030 (PMC7269239; doi:10.1371/journal.pone.0234030)
Supplement: Attachment — Submitted filename: review.docx [file pone.0234030.s015.docx]

The manuscript has been improved greatly both in writing and figure quality, and I think is suitable to be accepted. However, some more detailed information should be clarified.

1. The sensilla trichoidea 1, 2,... It means the subtype or just different sensilla on different location?

2. The sensila basiconica usually refers to the sensilla with the "cone" shape. The Fig. 3 and Fig.6 A is very similar. Why one is sensill basiconica and one is sensilla trichodiae? Based on reference 19, the SB in Fig.6 A named sensilla trichoidea will be suitalbe.

3 How to minumum the difference caused by the angle difference when the (nymph)antenne were mounted on to the holder? It is too diffcult to keep the nymph on the double stape with posture (to keep the sensilla to be scaned with same view) under a optical microscope. The length of the sensilla will be greatly different if scaned with different view.

4. With the Nova 85 Nano 430, it is posiable to find the pores on the sesilla trichoidae without gold coat. The sensilla on nymph antennae are very differnt with the sensilla on the adult antennae. However, in Ref. 19, they used the adult antennae; The antenne are from the nymph. I think the reader can get more useful information if the results about the sensilla from both nymph and adult are presented here.

In Line 49, "For most insects, the antennae are peripheral sensory." It is not suitalbe. In the paper Zoomorphology. 2017; 136(3): 327–347., peripheral refers the senilla on the antennae not the antennae.

In Line 55, the "spawning" usually used when discribe the aquatic animal. Rewrrtie this sentence please.

In line 226, "the number of the antennal sensiall was small" Compared with which insect or the insect?

The figure lengend shuold be polised again to help the reader obtain the information quikly(refer to referece paer 19 and 22). Figure legend is needed in Fig 3. ,Fig.4, Fig.7. There are only figure title with these fugures.
