# Supplementary material for: Morphological characterization of antennae and antennal sensilla of Diaphorina citri Kuwayama (Hemiptera: Liviidae) nymphs
Source: PLoS One. 2020 Jun 3;15(6):e0234030. doi: 10.1371/journal.pone.0234030 (PMC7269239; doi:10.1371/journal.pone.0234030)
Supplement: Attachment — Submitted filename: Reviewer comments R2.pdf [file pone.0234030.s012.pdf]

Reviewer comments

The manuscript entitled “Morphological characterization of nymphal antennae and antennal sensilla of *Diaphorina citri* Kuwayama (Hemiptera: Liviidae)’ reports general aspects of antenna/sensilla morphology of *Diaphorina citri*. The revision 2 was significantly improved, however major points need to be carefully corrected before its publication in PlosOne.

In my opinion, a final revision round is desirable in order to be sure that the paper is clear to be published.

Bellow, I will describe major points that should be addressed in order to clarify the manuscript content. However, detailed comments are noted in the pdf manuscript file.

Title suggestion: ‘Morphological characterization of antennae and antennal sensilla of *Diaphorina citri* Kuwayama (Hemiptera: Livvidae) nymphs.

Abstract: minor suggestions

Introduction: Minor suggestions

Results:

The entire topic: I struggled to understand/follow the presence and absence of structures for each nymphal stage. I encourage the authors to use Table and Figures citation in order to help the reader to understand the text without interruptions. (Suggestions attached – pdf file).

Standardization (antennae growth): I encourage the authors to add antennae growth in table 1 (and a statistical analysis for the new dataset). Add this information for second/third/forth and fifth instar topics and use same unit (sometimes µm, sometimes folds, sometimes information is missed) (check my comments in pdf file).

Discussion: I understand that the first sentence is introductory. But lines 223-231 is replicated. I identified this issue in the first review-round.

Tables: I encourage the authors to use only horizontal lines.
